# The Fucoxanthin Chlorophyll *a/c*-Binding Protein in *Tisochrysis lutea*: Influence of Nitrogen and Light on Fucoxanthin and Chlorophyll *a/c*-Binding Protein Gene Expression and Fucoxanthin Synthesis

**DOI:** 10.3389/fpls.2022.830069

**Published:** 2022-02-17

**Authors:** Anne Pajot, Johann Lavaud, Gregory Carrier, Matthieu Garnier, Bruno Saint-Jean, Noémie Rabilloud, Caroline Baroukh, Jean-Baptiste Bérard, Olivier Bernard, Luc Marchal, Elodie Nicolau

**Affiliations:** ^1^IFREMER, Physiology and Biotechnology of Algae Laboratory, Nantes, France; ^2^LEMAR-Laboratoire des Sciences de l’Environnement Marin, UMR 6539, CNRS/Univ Brest/Ifremer/IRD, Institut Universitaire Européen de la Mer, Technopôle Brest-Iroise, Plouzané, France; ^3^Université Côte d’Azur, Biocore, INRIA, CNRS, Sorbonne Université (LOV, UMR 7093), Sophia-Antipolis, France; ^4^Univ Nantes, GEPEA, Nantes, France

**Keywords:** carotenoids, fucoxanthin and chlorophyll *a/c*-binding proteins, Lhcf, Lhcr, Lhcx, *Tisochrysis lutea*, turbidostat culture, chemostat culture

## Abstract

We observed differences in *lhc* classification in Chromista. We proposed a classification of the *lhcf* family with two groups specific to haptophytes, one specific to diatoms, and one specific to seaweeds. Identification and characterization of the Fucoxanthin and Chlorophyll *a/c*-binding Protein (FCP) of the haptophyte microalgae *Tisochrysis lutea* were performed by similarity analysis. The FCP family contains 52 *lhc* genes in *T. lutea*. FCP pigment binding site candidates were characterized on Lhcf protein monomers of *T. lutea*, which possesses at least nine chlorophylls and five fucoxanthin molecules, on average, per monomer. The expression of *T. lutea lhc* genes was assessed during turbidostat and chemostat experiments, one with constant light (CL) and changing nitrogen phases, the second with a 12 h:12 h sinusoidal photoperiod and changing nitrogen phases. RNA-seq analysis revealed a dynamic decrease in the expression of *lhc* genes with nitrogen depletion. We observed that *T. lutea lhcx2* was only expressed at night, suggesting that its role is to protect \cells from return of light after prolonged darkness exposure.

## Introduction

Marine planktonic photosynthetic organisms are responsible for approximately 50% of Earth’s primary production and fuel the global ocean biological carbon pump (Field et al., 1998). Among these producers, 50% are represented by Chromista (Cavalier-Smith, 2004), including haptophytes, which are abundant primary producers. Haptophytes are ubiquitous, and they play an important role in ocean carbon fluxes (Jordan and Chamberlain, 1997; Green and Jordan, 2000; Liu et al., 2009; Edvardsen et al., 2016). They are of great interest for aquaculture and human health applications (Custódio et al., 2014; Matos et al., 2019; Mohamadnia et al., 2019; Leal et al., 2020). Along with other Chromista, haptophytes contain the most abundant photosynthetic carotenoid pigment in oceans, fucoxanthin (Fx), which shows numerous pharmacological properties including antioxidant, anticancer, and anti-diabetes activities (Arora and Philippidis, 2021).

In Chromista, light-harvesting complexes (LHCs) are composed of pigments and fucoxanthin chlorophyll *a/c*-binding proteins (FCPs). FCPs belong to the superfamily of transmembrane LHC proteins currently well-described in diatoms (Büchel, 2020b). FCPs bind photosynthetic pigments such as Fx, chlorophylls *a* and *c* (Chl *a/c*), and diadinoxanthin (Ddx), which can be de-epoxidized in diatoxanthin (Dtx) for photoprotection purposes (Lavaud et al., 2002, 2004; Kuczynska et al., 2015). In particular, the spatial organization of FCPs and their carotenoids confer to Chromista a very strong absorption capacity in the blue-green spectral range of visible light, which are the most available radiations in the water column (Wang et al., 2019a,b).

Studies on FCP protein sequences characterized three main families (Bhaya and Grossman, 1993; Apt et al., 1994, 1995), and two families whose function is unknown, Lhcz and Lhcq in diatoms, with Lhcq being commonly found in red algal PSI (Nagao et al., 2020; Kumazawa et al., 2021). Lhcf proteins are dominant, and they are attached to PSII (Nagao et al., 2019a; Pi et al., 2019). Lhcr proteins are attached to PSI (Nagao et al., 2020; Xu et al., 2020). Both Lhcf and Lhcr proteins mostly bind photosynthetic pigments such as Fx and Chl *a* and *c*. Finally, Lhcx proteins play a role in photoprotection (Bailleul et al., 2010; Taddei et al., 2016; Lepetit et al., 2017; Buck et al., 2019). It is assumed that Lhcx proteins bind both Ddx and Dtx in diatoms (Beer et al., 2006; Zhu and Green, 2010; Lepetit et al., 2013). FCP genes encoding for the three families are referred to as *lhcf*, *lhcr*, and *lhcx* (i.e., *lhc* genes).

Studies on FCP structure and *lhc* genes have been mostly carried out on diatoms, for which the photosynthetic and photoprotective mechanisms are now well-described (Goss and Lepetit, 2015; Jensen et al., 2017). In *Chaetocerosgracilis*, two FCP supercomplexes were characterized and are related to both photosystems, PSII-FCPII and PSI-FCPI. By cryo-electron microscopy (cryo-EM) analyses, it was found that PSI-FCPI presents different binding patterns of pigments (Nagao et al., 2020; Xu et al., 2020) compared to PSII-FCPII (Nagao et al., 2019a; Pi et al., 2019). In the model diatom *Phaeodactylum tricornutum*, FCPs are composed of 17 Lhcf proteins, 14 Lhcr proteins, and 4 Lhcx proteins (Büchel, 2020b). In an in-depth description of the light-harvesting antenna of *P. tricornutum* by crystallography analysis (Wang et al., 2019a), 9 binding sites for Chl *a* and *c* have been identified along with 7 binding sites for Fx, among 17 Lhcf sequences. Furthermore, a study on these binding sites demonstrated that Chl *a* and Fx molecules are closely related (i.e., each Fx is related to 1 Chl *a*) (Wang et al., 2019a). This close spatial proximity allows for efficient energy transfer and dissipation (Papagiannakis et al., 2005; Nagao et al., 2013; Akimoto et al., 2014; Gelzinis et al., 2015).

Fucoxanthin molecules shift absorption properties according to their amino acid binding sites on Lhcf monomers (Wang et al., 2019a). There exists three types of Fx molecules named according to their absorption properties: “Fx-blue” strictly absorbs in the blue range of visible light spectrum (λmax = 463 nm), “Fx-green” absorbs in a wider blue/green light range (λmax = 492 nm), and “Fx-red” additionally absorbs in an even wider blue/green light called “red-shifted” range (λmax = 500–550 nm) (Premvardhan et al., 2009). This specific shift in absorption properties explains the capacity of Fx to harvest photons over a wide light spectrum (Wang et al., 2019a), allowing Chromista to perform photosynthesis under a broad range of light conditions, including different habitats (water column, shallow sediments, sea-ice, etc.) and different depths (Di Valentin et al., 2012; Gelzinis et al., 2015; Büchel, 2020a).

Contrary to diatoms, FCPs of haptophytes have not been much described, except for *Emiliania huxleyi*. *E. huxleyi* FCPs are composed of 66 proteins (encoded by 75 genes), all named Lhcf and sub-divided into five groups: Lhcf group1, Lhcf group2, Lhcf (red), Lhcz-like, and LI818-like (Lefebvre et al., 2010; McKew et al., 2013). They are determined according to the classification of the LHC superfamily of Koziol et al. (2007) and have not been updated since. In Koziol et al. (2007) the LI818/LI818-like group is related to photoprotection, the Lhcr/Lhcc group is related to the LHC of red algae and cryptomonads, and the Lhcf group is related to FCPs; however, the specific function of the Lhcz group is still unknown. Because haptophytes are direct descendants of the cryptomonad lineage (Harper et al., 2005; Patron et al., 2007), the Lhcf (red) group of *E. huxleyi* was named after the Lhcr/Lhcc group.

In Chromista, light intensity and nitrogen availability are of a major influence on the photosynthetic process. Indeed, the concentration of pigments is changing with light wavelength and intensity (Marchetti et al., 2013; Nagao et al., 2019b; Yang and Wei, 2020; Duarte et al., 2021). It is known that low light increases photosynthetic pigment content (Brown et al., 1999; Anning et al., 2000; Mohamadnia et al., 2019; Gao et al., 2020a) and *lhcf* expression (Lefebvre et al., 2010) while high light increases quenching of excess energy with photoprotective pigments (Lavaud et al., 2002; Demmig-Adams et al., 2014), increases *lhcx* expression (Blommaert et al., 2020), and decreases *lhcf* expression (Lefebvre et al., 2010). Furthermore, it was shown in the haptophyte *Isochrysis galbana* that the carotenoid content decreased with nitrogen (N) deprivation (Zarrinmehr et al., 2019), and that, in a reverse manner, in the diatom *Odontella aurita*, an initial high concentration of nitrate boosted the accumulation of Fx in chloroplast membranes, and even more when coupled with N supplement (Xia et al., 2018). Several studies showed increase in pigment content with increase in N availability (Geider et al., 1998; Wever et al., 2008; Dahmen-Ben Moussa et al., 2017; McClure et al., 2018; Zarrinmehr et al., 2019). In batch culturing mode, however, as an increase in N availability generates an increase in biomass, light availability decreases over time as a culture develops. With less access to light, photosynthetic pigment content is increased and the expression of photosynthesis-related genes such as *lhc* genes is impacted. Therefore, the direct impact of N availability is hard to assess in the batch culturing mode. However, in continuous culturing mode with very limited variations in biomass, it is possible to measure pigment content per cell without the shelf-shading effect.

We selected *Tisochrysis lutea*, whose genome was sequenced (Berthelier et al., 2018), with the aim to update and complete *lhc* genes and FCP protein classification in haptophytes. This species contains high amounts of fucoxanthin and other carotenoids (Mohamadnia et al., 2019), whose biological activities are of great value in the field of aquaculture (i.e., bivalve hatcheries) (Delaunay et al., 1993; Rico-Villa et al., 2006) and biotechnologies (i.e., nutrition, pharmacology, etc.) (Delbrut et al., 2018; Cezare-Gomes et al., 2019). Our objectives were to identify for the first time *T. lutea lhc* genes, and classify them into the three families: *lhcf*, *lhcr* and *lhcx*. Then, we relied on a crystallographic study performed with *P. tricornutum* (Wang et al., 2019a) to determine some of chlorophylls and fucoxanthin binding site candidates on FCPs of *T. lutea*. We further analyzed the influence of N availability and light intensity on co-regulating the transcript levels of *lhc* genes. In parallel, we investigated the synthesis of carotenoids associated to these genes in *T. lutea*, Fx, Ddx, and Dtx.

## Materials and Methods

### Homologies of Fucoxanthin and Chlorophyll *a/c*-Binding Protein Sequences in Chromista

Before studying specifically *Tisochrysis lutea*, we collected FCP protein sequences of diverse microalgae from the literature (Supplementary Table 1). A similarity analysis of well-described 35 FCP sequences of *Phaeodactylum tricornutum*, taken as a reference, to all protein sequences available in Chromista was performed. Sequences with an *e*-value < 10^–40^ were available for eleven diatom strains (*Fragilariopsis solaris, Fragilariopsis cylindrus, Pseudo-Nitzschia multistriata, Nitzschia inconspicua, Thalassiosira pseudonana, Thalassiosira oceanica, Cyclotella cryptica, Cylindrotheca fusiformis, Skeletonema costatum, Chaetoceros gracilis*, and *Chaetoceros tenuissimus*), six haptophyte strains (*Emiliania huxleyi, Chrysotila carterae, Chrysochromulina tobinii, Isochrysis galbana, Pavlova lutheri*, and *Diacronema lutheri*), four ochrophytes (*Aureococcus anophagefferens, Nannochloropsis gaditana, Pelagomonas calceolata*, and *Heterosigma akashiwo*), four dinoflagellates (*Heterocapsa triquetra, Symbiodinium microadriaticum, Polarella glacialis*, and *Durinskia baltica*), three brown seaweeds (*Ectocarpus silicosus, Saccharina latissima*, and *Saccharina japonica*), and one alveolate (*Vitrella brassicaformis*).

### Identification of Lhcf, Lhcr, and Lhcx Proteins in *Tisochrysis lutea* Proteome

Lhcf, Lhcr, and Lhcx were identified by homolog search on the proteome of *T. lutea* generated *in silico* from sequenced genomes (Berthelier et al., 2018). We used as a query the FCP sequences of three diatoms, *Phaeodactylum tricornutum*, *Fragilariopsis* c*ylindrus*, and *Thalassiosira pseudonana* (Supplementary Table 2). A similarity analysis was performed with the Basic Local Alignment Search Tool (BLASTP). All targets in the *T. lutea* proteome were selected. We specified the three Lhc families of *T. lutea* by correspondence with best similarity matches with diatom sequences (Supplementary Tables 3–5).

With the Lhcf family representing the majority of FCP sequences, we additionally built a cladogram with the Lhcf sequences of *T. lutea*, of the four diatoms *Phaeodactylum tricornutum*, *Fragilariopsis* c*ylindrus*, *Thalassiosira pseudonana*, and *Chaetocerosgracilis*, of *Saccharina japonica* and *Saccharina latissima* (brown seaweeds), and of *Emiliania huxleyi* (haptophyte) (Supplementary Table 6). Using the software GENEIOUS^®^, we aligned 134 sequences with Clustal Omega and built a cladogram by neighbor-joining using the genetic distance model Jules-Cantor with 100 permutations. Bootstrap scores of the main nodes are displayed on the cladogram.

With the same method, we also built a cladogram representing the FCP sequences in *T. lutea* and another representing the Lhcr sequences of *T. lutea* and *E. huxleyi* in order to identify the subgroup Lhcz.

### Binding Sites of Chl *a*, Chl *c*, and Fx in *Tisochrysis lutea*

In a crystallographic study, binding sites of Chl *a*, Chl *c*, and Fx on Lhcf sequences of *P. tricornutum* were found (Wang et al., 2019a). We aligned Lhcf proteins of *T. lutea* with those of *P. tricornutum*. Then, we identified amino acid binding sites in *T. lutea* by comparing with those in *P. tricornutum*. Binding sites are of two different kinds. They are either central ligands or H-bond ligands, as described in *P. tricornutum*. Furthermore, majority of pigments possess several binding sites (up to four) and are not necessarily present in all Lhcf sequences of a species. Therefore, for each amino acid binding site, the percentage of presence in all Lhcf sequences was calculated.

### Culture Conditions

All the experiments were performed with *T. lutea* strain CCAP 927/14. Inocula were maintained in Walne’s medium (Walne, 1966) at 150 μmolm^–2^s^–1^. Both experiments were conducted in a continuous 8-L flat panel photobioreactor (1,000 mm × 400 mm × 250 mm).

#### Constant Light Experiment

To assess the effects of N availability on pigment content and s levels of *lhc* genes, we used and completed the data of a continuous culture in a chemostat performed by Garnier et al. (2016). A chemostat is a continuous culture with a controlled chemical environment and for which the dilution rate (ratio of inflow rate over reactor volume) is constant. *T. lutea* was grown for 85 days with a 0.5-day^–1^ dilution rate and illuminated with constant light (CL) (150 μmol m^–2^s^–1^). The dilution rate was periodically checked by weighing the output culture. Modified Walne’s medium containing 125/125 μM N:P ratio was used to induce limitation in N. The reactor was continuously aerated by bubbling and maintained at 27°C and pH of 7.3. Three nitrate (NO_3_) spikes consisting the injection of 3.5 mmol of NaNO_3_ in 10 L of culture were made on days 20, 43, and 83. The experiment was, therefore, run in consecutive triplicates. NO_3_ spikes during cultivation aim to subject the algae to different N regimes monitored with N quota (qN), i.e., intracellular N/C ratio analysis: balanced limitation phase (steady phase, qN = 0.09 molN molC^–1^), dynamic repletion phase (more dissolved N available in the culture medium, qN = 0.13molN molC^–1^), and dynamic depletion phase (less dissolved N available in the culture medium, 0.013 > qN > 0.09).

#### Dynamic Light Experiment

We performed a second experiment in turbidostat to understand the impact of both diel cycle and N availability on pigment content and transcript levels of *lhc* genes expression. A turbidostat is also a continuous culture, but dilution is varied in order to maintain cell density at a constant level (i.e., turbidity). *T. lutea* was grown at 23°C for 15 days with Walne’s medium (Walne, 1966). The light regime was sinusoid photoperiods of 12 h with maximal irradiance of 900 μmol m^–2^ s^–1^ at 12:00 h and darkness from 18:00 h to 00:00 h. Two NO_3_ spikes of 3.5 mmol NaNO_3_ were injected on days 1 and 8. The experiment was carried out two times under similar conditions (sequential duplicates). The culture was monitored in N repletion and N depletion phases (no dissolved N available in the culture medium) by sampling every 2 h. For transcriptomic analysis, the culture was harvested during the selected day at 8:00, 12:00, and 00:00 h for both N phases.

### Intracellular Nitrogen Concentration and Cell Concentration

The number of cells per unit of culture medium and intracellular carbon (C) and N were measured once a day for the CL experiment and every 2 h for the Dynamic Light (DL) experiment. Intracellular C and N were measured on an elemental analyzer (Flash, 2000; Thermo Fisher). Cell counting was performed using a Malassez hemocytometer.

### Pigment Extraction and High Performance Liquid Chromatography Analysis

For both experiments, we measured FCP pigment content: chlorophyll *a* (only in the DL experiment), Fx, Ddx, and Dtx. For the CL experiment, samples were centrifuged, and wet pellets were stored at −80°C. Each pellet with a known number of cells (250–50,010^6^ cells) was dispersed in 5 ml of 95% acetone, vortexed, and then mixed for 3–9 min at 30 Hz frequency in a mixer-mill (Retsch MM-400) with0.74g of 1-mm glass beads. Acetone extract was filtered with a0.2-μm polytetrafluoroethylene (PTFE) filter before immediate high performance liquid chromatography (HPLC) analysis. For the DL experiment, 15.1^6^cells were harvested in a0.2-μm fiberglass filter, and then immersed in 2 ml 95% acetone overnight. Acetone extracts were filtered with a 0.2-μm fiberglass filter prior to injection in HPLC.

The filtered acetone extracts were analyzed by HPLC-UV-DAD (series 1200 HPLC-UV-DAD; Agilent Technologies) using an Eclipse XDB-C8 reverse phase column (150 mm × 4.6 mm, 3.5 μm particle size; Agilent Technologies) following the method described by Van Heukelem and Thomas (2001). Briefly, solvent A was 70:30 MeOH: H_2_O 28 mM ammonium acetate, and solvent B was pure MeOH. Gradient elution was the same as described in Van Heukelem and Thomas (2001). Quantification was carried out by external calibration against pigment standard [Chl *a* for the DL experiment, and Dtx, Ddx and Fx for both experiments, provided by DHI (Denmark)].

### RNA Sequencing

A total of 50 ml of algal culture was sampled for transcriptomic analysis. Total RNA was extracted and sequenced as described by Carrier et al. (2014). A library of RNA-seq was built for each sample. Poly(A) mRNA was isolated from total RNA using oligo (dT) magnetic beads [MicroPoly(A)Purist™ Kit; Ambion]. Two cDNA libraries were built according to the instructions of the manufacturer and sequenced with an Illumina HiSeq 3000 sequencer and with the GenoTool platform. We obtained an average of 2.6.1^7^ and 3,2.1^7^ reads per sample in the CL and DL experiments, respectively.

Raw reads of each sample were filtered using TrimGalore to remove known Illumina adapter sequences. Low-quality reads were excluded using a quality score threshold of 30 and a minimal length of 75 or 150 bases. The quality of reads was assessed using FastQC. Then, sequenced reads for each sample were aligned using Bowtie 2 in the QuasR package in R (Quantify and Annotate Short Reads in R). Each gene within the alignment was counted using the Rsubread package in R with the function feature Counts, based on *T. lutea* reference genome (Carrier et al., 2014). Gene counts were obtained for each sample and normalized with DESeq2.

### Statistics

In order to analyze the influence of N status (qN) and light intensity on *lhc* gene transcript level in *T. lutea*, a differential expression (DE) analysis of *lhc* genes was performed on R using the DESeq2 tool. The global influence of qN and light was determined with the fold change and adjusted *p*-value (< 0.05) of the DESeq2 comparison of all transcripts in each culture condition. The resulting mean average plot (MA-plot) allows for visual identification of a significant difference in expression of all transcripts (Supplementary Figures 5–10).

The fold change and adjusted *p*-value of the corresponding 52 transcripts in the resulting table of the DESeq2 comparison were selected to observe if their expression was significantly influenced by qN and light intensity. In addition, an ANOVA was performed on the 52 transcript counts between each condition.

## Results

### Homologous Fucoxanthin and Chlorophyll *a/c*-Binding Protein Sequences in Chromista

Our comparative analysis of sequences available in Chromista revealed that among all the sequences similar to those in *P. tricornutum*, the majority is classified as unknown protein or as hypothetical light-harvesting proteins. In two diatoms (*Fragilariopsis cylindrus* and *Thalassiosira pseudonana*) and one haptophyte (*Emiliania huxleyi*), sequences are classified as LHC proteins (i.e., FCPs). These results lead us to study more specifically the FCP proteins of the FCP complex in *T. lutea* coded by the *lhcf*, *lhcr*, and *lhcx* genes.

### Lhcf, Lhcr, and Lhcx Annotation in the Proteome of *Tisochrysis lutea*

**FIGURE 1:**
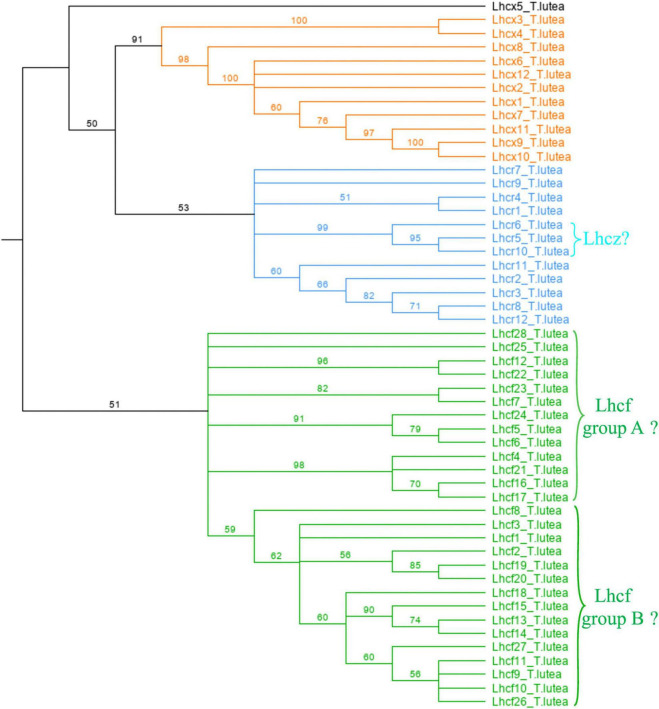
Cladogram of the 52 Lhcf sequences of *Tisochrysis lutea*. Orange: Lhcx. Blue: Lhcr. Green: Lhcf.

Our analysis revealed that 52 FCP proteins constitute the FCP in *T. lutea* (Figure 1 and Supplementary Table 7). All of them matched to either the Lhcf, Lhcr, or Lhcx -family of diatom FCPs. According to similarity scores obtained with *P. tricornutum*, *F. cylindrus*, and *T. pseudonana* (Supplementary Tables 3–5), among the sequences of *T. lutea*, 28 were Lhcf, 12 were Lhcr, and 12 were Lhcx (Figure 1). In *T. lutea*, Lhcf23 was been classified as Lhcr after one single match with low score in BLAST results, but the cladogram (Figure 1) clustered this sequence into the Lhcf family. It is likely that the algorithm did not display all lowest scores in the similarity search BLAST. Knowing it is classified as an Lhcf sequence with the cladogram, a new similarity search was performed between this sequence and the Lhcf sequences of all the three diatoms. We observed a very few matches of Lhcf23 with Lhcf diatom sequences, with scores in the same range compared to the previous match with the Lhcr sequence of *F. cylindrus*. We conclude, according to the BLAST results only, that the probability of Lhcf23 belonging to either the Lhcr or Lhcf family was the same, but according to the cladogram, it was decided to be classified as an Lhcf sequence. Furthermore, it was not possible to classify the Lhcx5 sequence with certitude, because although the BLAST results were slightly higher than the Lhcx of the three diatoms (Supplementary Tables 3–5), it did not appear as pertaining to the Lhcx family on the cladogram (Figure 1).

**FIGURE 2:**
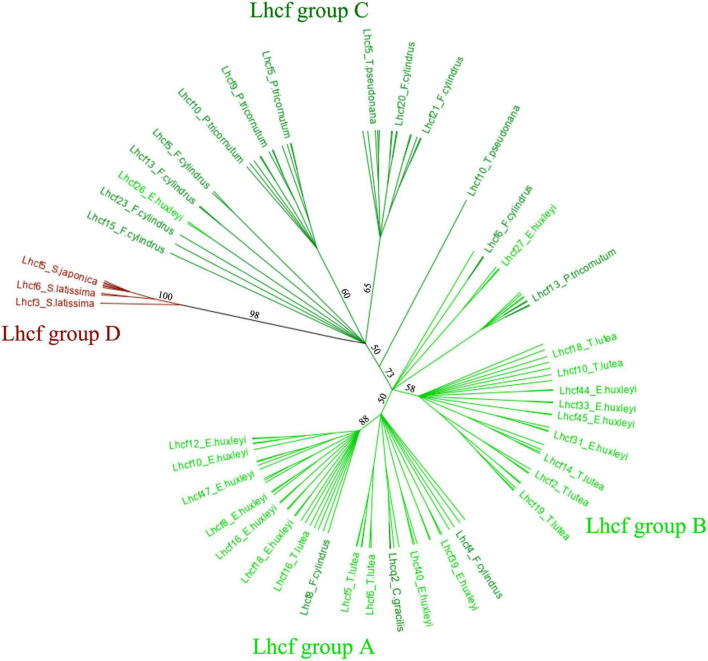
Cladogram of the 134 Lhcf sequences of eight species, *Phaeodactylum tricornutum, Fragiliaropsis cylindrus, Thalassiosira pseudonana, Chaetocerosgracilis, Saccharina japonica, Saccharina latissima, Emiliania huxleyi*, and *Tisochrysis lutea*. Light green: haptophyte Lhcf. Dark green: diatom Lhcf. Dark red: seaweed Lhcf.

To further analyze the Lhcf proteins, major sequences of the FCP, we built a cladogram showing four main well-supported clades of Lhcf in haptophytes, diatoms, and brown seaweeds (Figure 2). Consequently, complementary to sequence classification in diatoms (Gundermann et al., 2013), we propose a new classification of FCPs for haptophytes (Table 1). Lhcf group A sequences are mainly found in haptophytes, Lhcf group B sequences are exclusively found in haptophytes, Lhcf group C sequences are exclusively found in diatoms, and Lhcf group D sequences are exclusively found in brown seaweeds. Based on this cladogram, we observed that the Lhcf groups I and II of *E. huxleyi* described in McKew et al. (2013) correspond to the Lhcf groups A and B, respectively. Furthermore, we aligned all the FCP sequences of *E. huxleyi* and found two other distinct groups: all sequences of the Lhcf (red) and LI818-like groups in *E. huxleyi* (McKew et al., 2013) correspond the Lhcr and Lhcx groups (Supplementary Table 6 and Supplementary Figure 1), respectively. It appeared that one subgroup of the Lhcr family corresponds to the Lhcz-like family described in McKew et al. (2013) By building a cladogram between the Lhcr of *E. huxleyi* and those of *T. lutea* (Supplementary Figure 2), we observed two Lhcr subgroups, one of which was composed of Lhcz-like of *E. huxleyi* along with three Lhcr proteins of *T. lutea* (Lhcr5, Lhcr6, and Lhcr10), which might, therefore, be Lhcz sequences. We propose a new annotation of the 75 *E. huxley ilhc* genes into the three *lhcf*, *lhcr*, and *lhcx* families (Supplementary Table 6), and we hypothesize the existence of a fourth family being that of Lhcz, both in *E. huxleyi* and *T. lutea*.

**TABLE 1:** Proposal for a new classification of Lhcf.

*Lhcf* groups	Haptophytes	Diatoms	Brown seaweeds
*Lhcf* group A	+++	+	−
*Lhcf* group B	+++	−	−
*Lhcf* group C	−	+++	−
*Lhcf* group D	−	−	+++

### Binding Sites of Chlorophylls and Fucoxanthin in Lhcf Proteins in *Tisochrysis lutea*

**FIGURE 3:**
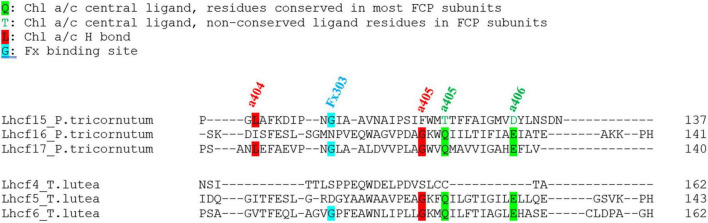
Example of a part of the alignment of three Lhcf sequences of *P. tricornutum* and three Lhcf sequences of *T. lutea* with binding sites of three Chl *a* (a404, a405, and a406) and one Fx (Fx303).

All Lhcf sequences of *P. tricornutum* and *T. lutea* were aligned, and pigment binding site candidates were identified in *T. lutea* (Figure 3 and Supplementary Figure 3).

Tables 2, 3 present the different types of ligands for Chl *a*, Chl *c*, and Fx in Lhcf sequences of *P. tricornutum* compared to those of *T. lutea* in same amino acid positions. A majority of pigments possess several binding sites (e.g., Chl a404, Fx302).

**TABLE 2:** Binding sites of Chl *a* and *c* amino acid (in capital letters) in *Tisochrysis lutea* according to those identified in *Phaeodactylum tricornutum* (Wang et al., 2019a).

		Central ligand				H bond	
Chl identified in *P. tricornutum*		Conserved	%_among Lhcf_	Not conserved	%_among Lhcf_		%_among Lhcf_
Chl a401	*P. tricornutum*	P	35%			N	77%
						Q	65%
	*T. lutea*	P	61%			N	43%
						Q	0%
Chl a402	*P. tricornutum*	E	100%			F	59%
	*T. lutea*	E	89%			F	29%
Chl c403	*P. tricornutum*	H	100%			R	71%
	*T. lutea*	H	89%			R	50%
Chl a404	*P. tricornutum*	Q	47%	T or V	53%	L	24%
	*T. lutea*	Q	14%	T or V	43%	L	29%
Chl a406	*P. tricornutum*	E	82%	D	18%	K	100%
	*T. lutea*	E	75%	D	18%	K	89%
Chl a405	*P. tricornutum*	Q	88%	A or T	12%	G	94%
	*T. lutea*	Q	89%	A or T	0%	G	71%
Chl a407	*P. tricornutum*	E	100%			F	65%
	*T. lutea*	E	93%			F	14%
Chl c408	*P. tricornutum*	Q	65%	N or H	35%	K	65%
	*T. lutea*	Q	0%	N or H	93%	K	68%
Chl a409	*P. tricornutum*	H	88%	E or S	12%		
	*T. lutea*	H	61%	E or S	4%		

*Percentages represent the presence of amino acid binding sites among all the Lhcf sequences of each species. The central ligand of Chl a and c is or is not conserved (shifted or new amino acid position) compared to Chl a and b binding sites of the LHCII of plants.*

**TABLE 3:** Binding sites of Fx amino acid (in capital letters) in *T. lutea* according to those identified in *P. tricornutum* (Wang et al., 2019a).

		Central ligand	
**Fx identified in *P. tricornutum***			**%_*among Lhcf*_**
Fx 301	*P. tricornutum*	L	77%
	*T. lutea*	L	50%
	*P. tricornutum*	R	100%
		T	24%
Fx 302		Y	25%
		M	71%
	*T. lutea*	R	93%
		T	0%
		Y	54%
		M	0%
	*P. tricornutum*	F	59%
Fx 303		G	94%
	*T. lutea*	F	29%
		G	68%
Fx 304	*P. tricornutum*	K	65%
	*T. lutea*	K	68%
Fx 305	*P. tricornutum*	L	24%
	*T. lutea*	L	18%

*Percentages represent the presence of binding sites among all the Lhcf sequences of each species.*

All Chl *a-* and *c-*conserved central ligands in *P. tricornutum* (excepted for c408) corresponded to homologous domains in *T. lutea*. All non-conserved central ligands (except for a405) were located in *T. lutea.* All H-bond sites (excepted for a401) were located in *T. lutea*. *T. lutea* conserved the same Fx binding sites compared with *P. tricornutum* (Table 3), except for 2 binding sites of Fx302. Among the 7 Fx of *P. tricornutum*, the comparison with T. lutea could only be done upon 5 which are Fx301, 302, 303, 304, 305. Among these five, four Fx binding sites in *P. tricornutum* corresponded completely to homologous amino acid domains in *T. lutea*. However, the Fx302 in *P. tricornutum* had four binding sites, R, T, Y, and M but only R and Y were found in *T. lutea*. More precisely, the Y binding site in *P. tricornutum* corresponded to a Y on a 3 aa shifted position in *T. lutea*. This position corresponded to the T binding site of Fx302 in *P. tricornutum* (Supplementary Figure 2), highlighting the relevance of this protein section in binding an Fx molecule.

According to these results, 9 binding sites of Chl *a* and *c*, and 5 binding sites of Fx (for multiple sites, at least one is found) are found in *T. lutea* and described in Supplementary Figure 3. Another observation is that except for the Chl a404 central ligand and the Chl a407 H bond ligand, the percentages of the presence of binding sites among all *T. lutea* Lhcf sequences were very close to those in *P. tricornutum*.

### Effects of Nitrogen Availability and Light Intensity on the Expression of *Lhc* Genes

#### Constant Light Experiment: N Quota and Biomass

**FIGURE 4:**
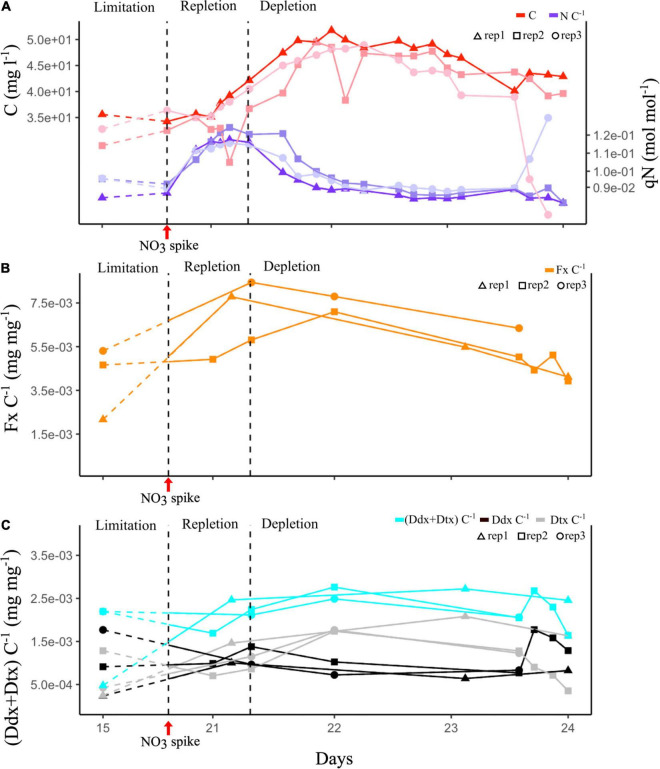
Constant light (CL) experiment. Dashed curve corresponds to the first 5 days of culture under N limitation conditions. **(A)** Evolution of cell biomass expressed as total intracellular C and of qN expressed as intracellular N:C ratio of three culture replicates. **(B)** Evolution of the Fx:C ratio of three culture replicates. **(C)** Evolution of the photoprotective pigment ratios: (Ddx + Dtx):C, Ddx:C, and Dtx:C of the three culture replicates. Vertical black dashed lines mark the transition between limitation and repletion, and repletion and depletion in N.

After NO_3_ spike, qN (i.e., N quota, intracellular N/C ratio analysis performed to determine the N regime of the cultures) immediately increased, cells entered the repletion phase for 12 h. The biomass started to increase 10 h after the NO_3_ spike. qN decreased within 24 h after reaching its maximum (0.13 mol N mol C^–1^), and it is the depletion phase. The biomass increased for 24 h before stabilizing for another 24 h, and finally it decreased. Eventually, cells reached a new steady phase with constant qN and biomass (Figure 4A).

#### Constant Light Experiment: Pigments

Figures 4B,C s an increase in Fx, Ddx, and Dtx content in cells (expressed as Fx:C, Ddx:C, and Dtx:C ratio) concurrently with qN (Figure 4A) during the repletion phase. Fx and Ddx contents were tripled and were at maximum when qN was maximum, and then slightly decreased during the depletion phase. In contrast, Dtx content was doubled during depletion (Figure 4C). The Ddx + Dtx pool was up to five times higher during the dynamic phases of the experiment repletion and depletion phases (2.5e-03 mg mg^–1^) than during the limitation phase (5.0.e-04 mg mg^–1^).

#### Constant Light Experiment: Expression of *Lhc* Genes

**FIGURE 5:**
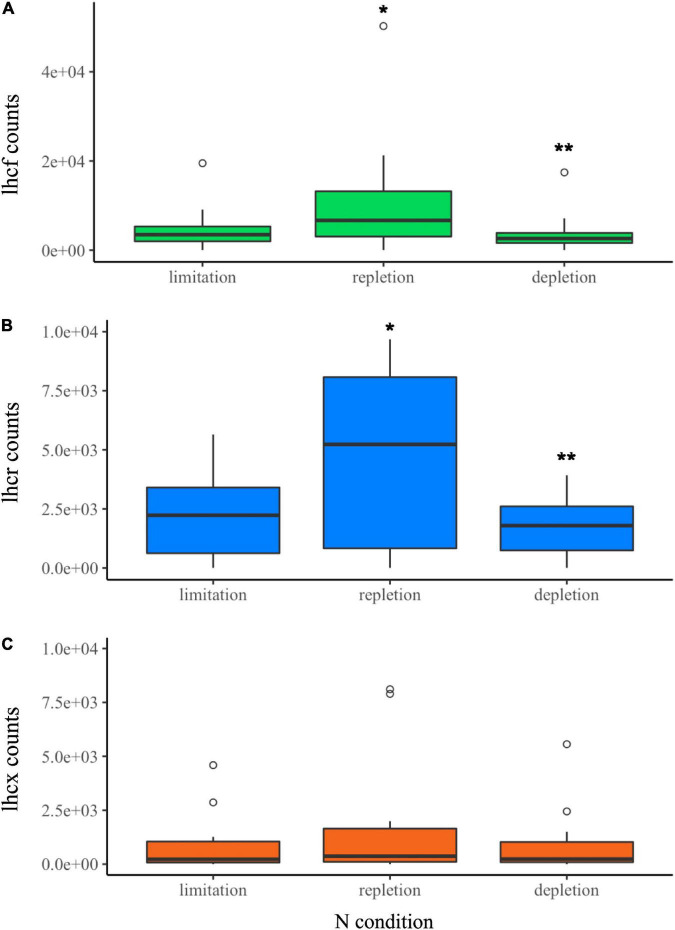
CL experiment. Normalized transcript counts of **(A)** *lhcf* (green), **(B)** *lhcr* (blue), **(C)** *lhcx* (orange) genes according to N status (limitation, repletion, and depletion). Analysis of variance (ANOVA) test: *p-value < 0.05, **p-value < 0.01.

The evolution of FCP gene counts has been studied as a function of N availability in the same cultures. Transcriptomics analyses were performed on two replicates. We observed an increase in all *lhcf*, *lhcr* and *lhcx* transcript counts during the repletion phase compared to limitation, and a decrease in depletion phase compared to repletion (Figure 5 and Supplementary Tables 8–10). The global analysis of variance did not show significant differences in *lhcx* counts among all the N phases. However, specific analysis of genes revealed that two *lhcx* genes were regulated with significant differences. Along with one *lhcf* outlier (*lhcf13*), *lhcx2*, and *lhcx5* gene counts were between 3 and 20 times higher than the median value of other *lhcf* and *lhcx* genes in each N condition (Figure 5). No expression was recorded for *lhcr9.*

The repletion phase was significantly correlated (DE *p*-value < 0.05) with an increase of the expression of several *lhc* genes. 11 *lhcf* over 28, 2 *lhcr* over 12 and 4 *lhcx* over 12 were 1.5 to 4 times more expressed than during the limitation phase (Supplementary Table 17). During the depletion phase, the expression was significantly lower than repletion, between 3 and 5.5 times for 22 *lhcf* genes over 28, 8 *lhcr* genes over 12, and 3 *lhcx* genes over 12 (Supplementary Table 17).

Looking at the CL experiment, it turned out that in parallel with the lower concentration of the pigments measured, the depletion in N corresponded to the lowest expression of the *lhc* genes.

#### Dynamic Light Experiment: N Quota and Biomass

**FIGURE 6:**
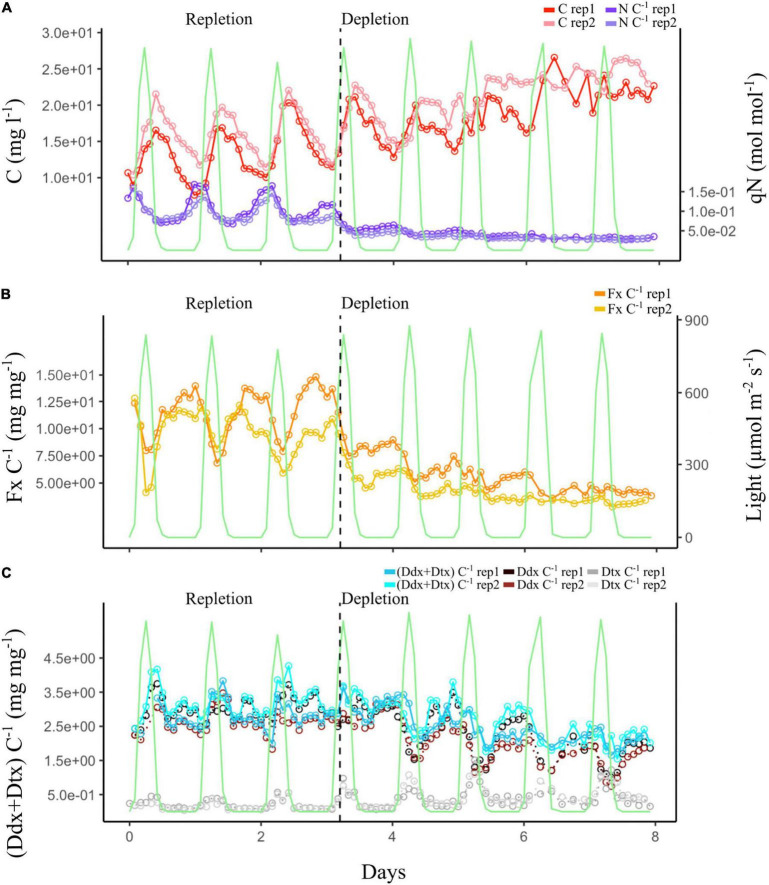
Dynamic light (DL) experiment. The green curve represents photosynthetically active radiation (PAR) during the day:night cycle. **(A)** Evolution of the cell biomass expressed as total intracellular C and of the qN expressed as intracellular N:C ratio of the two culture replicates. **(B)** Evolution of the Fx:C ratio of the two culture replicates. **(C)** Evolution of the photoprotective pigment ratios: (Ddx + Dtx):C, Ddx:C, and Dtx:C of the two culture replicates. Vertical black dashed line marks the transition between the repletion and depletion phases in N.

In the DL experiment, the biomass was kept constant between 1.0 1^6^ and 1.5 1^6^ cells ml^–1^ (Figure 6A). qN and Fx content (Fx:C) changed according to N bioavailability in the culture, following a pattern similar to the one in the CL experiment. During the repletion phase, qN followed the light signal (day:night cycles), reaching a minimum and a maximum value, respectively, at 00:00 and 12:00 h (Figure 6B). During depletion, qN was not significantly impacted by light variation and decreased for 24 h before it stabilized at its minimum value. During the repletion phase, the biomass evolved in correlation with the day:night cycles. When the light intensity reached 10 μmol photons m^–2^ s^–1^ around 06:00 h, the algal biomass increased until the light intensity was back to 10 μmol photons m^–2^ s^–1^ at 18:00 h, and it further decreased during the night. However, during the depletion phase, a difference was observed. Biomass correlation with the day:night cycles was less distinct, and the difference in biomass concentration between day and night was reduced.

#### Dynamic Light Experiment: Pigments

Whatever the N phase of the cultures, Fx content (Figure 6B) and Chl *a* content (Supplementary Figure 4) increased during the night and decreased during the day. During N depletion, however, the influence of the day:night cycles on Fx, Chl *a*, and Ddx (Figure 6C) was minimized, as their content were two to three times lower than during repletion. On the contrary, during N repletion, the influence of the day:night cycles on Dtx content was barely noticeable, as it was two times lower than during depletion. On the contrary, Dtx content increased during the day and drastically decreased during the night all along the experiment (Figure 6C). It was doubled in depletion compared to in repletion. However, the Ddx + Dtx pool decreased in depletion, mainly influenced by Ddx content.

Overall, these results corroborated those of CL experiment, apart from the Ddx + Dtx pool, which decreased during the N depletion phase.

#### Dynamic Light Experiment: Expression of *Lhc* Genes

**FIGURE 7:**
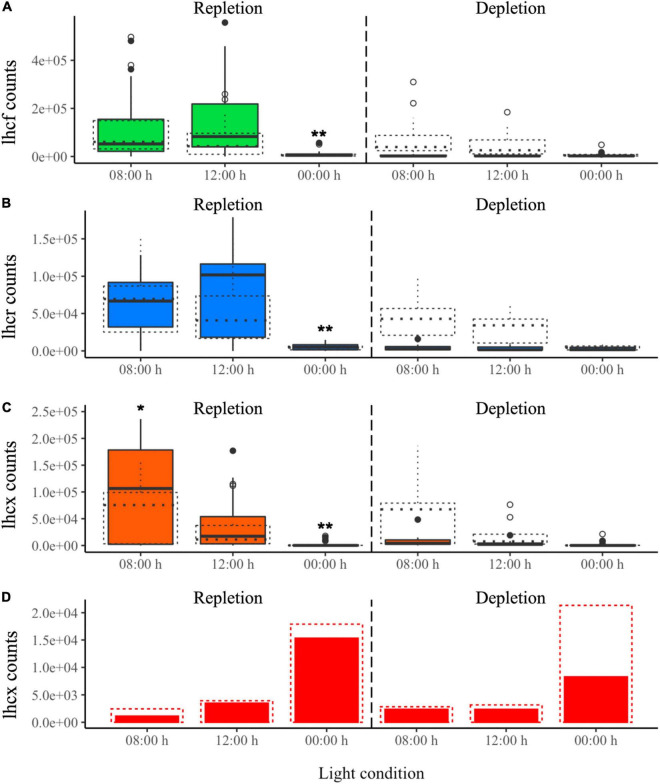
DL experiment. Normalized transcript counts of **(A)** *lhcf* (green), **(B)** *lhcr* (blue), **(C)** *lhcx* (orange) genes and **(D)** *lhcx2* (red) gene according to N status (repletion and depletion) and day:night cycles. ANOVA test: *p-value < 0.05, **p-value < 0.01. The experiment was realized in duplicate and represented by solid (replicate 1) and dotted lines (replicate 2). Vertical black dashed line marks the transition between the repletion and depletion phases in N.

In the DL experiment, *lhc* expression varied according to both N bioavailability and light intensity (Figure 7). Similar to the CL experiment, gene counts were lower in the N depletion than in the repletion phase. It is especially highlighted with gene counts of replicate 1. Also, genes were more expressed in the repletion phase compared to the CL experiment. All the gene counts are available in supporting information (Supplementary Tables 11–16).

Most of the genes were upregulated during the day at 8:00 and 12:00 h and downregulated at night (00:00 h) (Figures 7A–C). Specifically, *lhcx* genes were significantly upregulated at 8:00 h rather than at 12:00 h, except for *lhcx2*, which was the only gene expressed exclusively during the night (Figure 7D and Supplementary Table 18). Also, *lhcf21* and *lhcr6* were the only *lhcf* and *lhcr* genes to be significantly upregulated at 8:00 h vs. 12:00 h (Supplementary Table 19).

The DE analysis reveals that the percentages of upregulation in the repletion phase compared to depletion were similar to those in the CL experiment. However, these results were for replicate 1 only. Indeed, in replicate 2, no *lhc* gene was significantly downregulated in depletion compared to repletion. We also confirmed that the great majority of the *lhcf*, *lhcr* and *lhcx* genes was significantly upregulated during the day (Table 4). Another observation was the high percentage of *lhcx* significantly upregulated at 8:00 h compared to 12:00 h (25%, *lhcx3*, *lhcx 11*, and *lhcx12*).

**TABLE 4:** Percentages of *lhcf*, *lhcr*, or *lhcx* genes upregulated at 8:00 and 12:00 h in the dynamic light (DL) experiment.

	Up-regulation of *lhc* genes		
	At 8:00 h vs. 12:00 h	At 8:00 h vs. 00:00 h	At 12:00 h vs. 00:00 h
*lhcf*	3.6%	82%	68%
*lhcr*	8.3%	82%	92%
*lhcx*	25%	75%	75%

## Discussion

### Fucoxanthin and Chlorophyll *a/c*-Binding Protein Sequences in Chromista Species

Only a few annotated FCP sequences were available for haptophytes compared to the data available in literature for other groups of Chromista. The known classification in diatoms supported the annotation of the 52 FCP sequences in the haptophyte *Tisochrysis lutea*. Our results also emphasized the diversification of the Lhcf family: we propose the Lhcf group A mainly to be composed of haptophytes sequences, the Lhcf group B to be specific to haptophytes, the Lhcf group C to be specific to diatoms, and the Lhcf group D to be specific to brown seaweeds. These groups may indicate that the evolutionary distance between diatoms and haptophytes is also reflected in their light-harvesting complexes (Büchel, 2020a). As the novel group named Lhcq was proposed in diatoms (Nagao et al., 2020; Kumazawa et al., 2021), it is not excluded that some *T. lutea* sequences pertain to this group. However, there is not enough information, so far, to confirm it. Furthermore, our results suggested that three Lhcr proteins (Lhcr6, Lhcr10, and Lhcr11), in *T. lutea* belong to the *Lhcz* group, defined as a sister clade of Lhcr in Kumazawa et al. (2021). Overall, the *lhc* genes in *T. lutea* are 54% *lhcf*, 23% *lhcr* (including *lhcz*), and 23% *lhcx*, while in *E. huxleyi* they are, respectively, 70, 9, and 21%. While the proportion of photoprotection-related Lhcx was similar between both species, *T. lutea* overall FCP composition is enriched in PSI-related Lhcr, while it is enriched in PSII-related Lhcf in *E. huxleyi*. In parallel, we know that the amount of light harvested by PSII and PSI is determined by the effective absorption cross-sections, σ_PSII_ and σ_PSI_, respectively. In two strains of *E. huxleyi*, the ratio of σ_PSII_:σ_PSI_ was found to be ∼0.4–0.5 (Suggett et al., 2007), while in *Isochrysis galbana*, a species close to *T. lutea*, it was found to be ∼0.25–0.35 (Dubinsky et al., 1986). As σ_PSII_:σ_*PSI*_ is higher in *E. huxleyi*, this could mean that light harvesting is more effective in the PSII in *E. huxleyi* than with *I. galbana*, which is more effective in PSI. Supposing that the σ_PSII_:σ_PSI_ ratio in *T. lutea* is similar to that in *I. galbana*, this observation is consistent with the FCP composition of both species.

Possibly, this distinction could be linked to a different adaptation to the *in situ* light climate with *T. lutea* originating from an environment with strong light variations and requires stronger balance between PSII and PSI photochemistry. In comparison, *E. huxleyi* is found in coastal, temperate continental, and cold regions with less harsh light intensity growing conditions (Brown and Yoder, 1993; Perrin, 2017) but still requires great ability to tolerate different light intensities (Cortés et al., 2001).

### Binding Sites of Chlorophylls and Fucoxanthin in Lhcf Proteins in *Tisochrysis lutea*

In the Lhcf proteins of *P. tricornutum*, the most red-shifted, Fx306 and Fx307, were directly bound to Chl *c* (Wang et al., 2019a). Therefore, even if these two candidate sites were found on the Lhcf of *T. lutea*, it is not possible to certify that these Chls effectively bind Fx molecules. This could only be observed with a crystallographic or a cryo-EM study, as it was done on *P. tricornutum* (Wang et al., 2019a) and *C. gracilis* (Kumazawa et al., 2021). Amino acid homologies between *P. tricornutum* and *T. lutea* FCP sequences strongly suggested that *T. lutea* possesses at least 9 Chl *a* or *c* binding-sites among the majority of Lhcf monomers. Most of the Fx binding sites identified in *P. tricornutum* were also found in *T. lutea*, although none of the Lhcf proteins showed all the Fx binding sites identified in *P. tricornutum*. This result suggests the presence of Fx binding sites specific to haptophytes as compared to diatoms. Among the 28*T. lutea* Lhcf proteins, three (Lhcf7, Lhcf11, and Lhcf13) showed a few of the identified Chl and Fx binding site candidates. It suggests that they rather have an important structural role in light-harvesting spatial organization. In particular, Lhcf13 was the most expressed in our experiments (Supplementary Tables 8, 11, 12). These results are to be carefully considered, because transcripts could be regulated before translation into proteins (Sonenberg and Hinnebusch, 2009). Over all, and although the exact oligomeric spatial organization of the light-harvesting system of *T. lutea* remains unknown, sequence comparison with *P. tricornutum* strongly suggested that the light-harvesting capacity of haptophytes is similar to that of diatoms.

### *Lhcf* and *Lhcr* Expression and Fucoxanthin Synthesis

#### Influence of Nitrogen

In *P. tricornutum*, the FCP and general photosynthesis pathway are downregulated during N stress (Longworth et al., 2016). In *I. galbana*, a close strain of *T. lutea*, it was suggested that the FCP is a constant fraction of the total qN of about 6.8% (Falkowski et al., 1989). Both of our experiments showed that qN decreased by up to 1.6 times in N limitation and depletion compared to N repletion. It was, therefore, consistent to observe, from N limitation to N repletion phase, an increase in the expression of the photosynthetic genes *lhcf* and *lhcr*, an increase in Fx content, and a distinct decrease from repletion to depletion. In parallel, photosynthetic pigment content decreased during depletion. These results might indicate the correlation of the binding of Fx and Ddx on Lhcf and Lhcr. Furthermore, they demonstrate the positive influence of increase in qN on Fx and Ddx content and on the expression of the majority of *lhc* genes, significantly for 34 *lhc* genes including *lhcx*. The DL experiment showed that Chl *a* content varied similarly to the Fx content (Supplementary Figure 3). The positive linear correlation between Chl *a* and Fx in *T. lutea* has already been reported in a study (Gao et al., 2020b). As chlorophylls contain N, contrary to carotenoids, they are positively influenced by N supplementation, which can explain the indirect dependence of Fx on N concentration in the culture. Furthermore, Chl *a* binds to Lhcf and Lhcr proteins. Therefore, there is a correlation between the N needed for Chl *a* synthesis and the Lhcf and Lhcr proteins needed for Chl *a* binding.

#### Influence of Light

Experiments with a photoperiodic cycle as our DL experiment can reveal the dynamic shift between photosynthesis and photoprotection. In our experiments, we observed that *lhcf* and *lhcr* genes were upregulated during the day, and were almost not expressed at night, contrary to photosynthetic pigments. On one hand, and as expected, Fx and Ddx content increased with low light intensity to perform photosynthesis even with a few photons. Furthermore, several studies hypothesized that Ddx is a precursor of Fx synthesis (Dambek et al., 2012; Dautermann et al., 2020), enhancing the fact that both pigments evolve simultaneously. On the other hand, the fact that *lhcf* and *lhcr* genes were not upregulated during the night was unexpected. We supposed that cells synthesized the whole FCP complex during the day, allowing for the binding of photoprotective pigments while preparing the need for photosynthetic pigments when the light decreases at night. This shift might explain a trade-off between the need for photoprotection during the day and the light-harvesting process mainly active at the beginning of the night. When comparing both experiments, *lhc* genes were more expressed in repletion with a day:night cycle than in repletion with constant light, which indicates that light intensity is a more impacting factor than N for FCP expression.

Finally, the qN varying according to NO_3_ concentration in the culture medium, along with light intensity, is partly responsible for the balance between the photosynthetic and photoprotective activities of *T. lutea*.

### *Lhcx* Expression, Diadinoxanthin, and Diatoxanthin Synthesis

The photoperiodic cycle of the DL experiment allowed to distinguish the photoprotection dynamic between the *lhcx* and the Ddx/Dtx content. At 8:00 h, after 2 h of illumination, cells expressed more *lhcx* than at 12:00 h. In parallel, we observed that Dtx content was higher during the day, corroborating its role in photoprotection. These results were in line with what was found in diatoms with increase, under high light, in Dtx content in *Cyclotella meneghiniana* (Beer et al., 2006), in both Dtx content and LI818-like proteins (also referred as Lhcx) in *T. pseudonana* (Zhu and Green, 2010), in the Ddx + Dtx pool and three *lhcx* in *P. tricornutum* (Lepetit et al., 2013), and in the Ddx + Dtx pool and synthesis of Lhcx2 and Lhcx3 in *P. tricornutum* (Blommaert et al., 2017; Lepetit et al., 2017). Furthermore, a tryptophan residue located closely to the Ddx/Dtx binding site of Lhcx was characterized as involved in energy-dependent fluorescence quenching (qE) in *P. tricornutum*, a photoprotection mechanism (Buck et al., 2021). Dtx results from the de-epoxidation of Ddx; it was, therefore, consistent to observe that Dtx content was reversely symmetrical to Ddx content in both of our experiments. However, even under maximum light intensity, Ddx was not entirely de-epoxidized in Dtx, highlighting its double role as a photosynthetic and a photoprotective pigment (Lacour et al., 2020). Overall, the Ddx + Dtx pool increased with light intensity in repletion as what was found in *Cyclotella meneghiniana* when applying high light (Gundermann and Büchel, 2008), supporting the main photoprotective role of these pigments. Finally, *T. lutea* cells seemed to activate their photoprotection mechanisms to endorse light onset without risking the deterioration of cellular mechanisms.

Similarly, in *C. meneghiniana*, proteins encoded by an *lhcx* named *fcp6/7* were more present under HL conditions (Oeltjen et al., 2002; Beer et al., 2006; Gundermann and Büchel, 2008). In addition, *lhcx2* in *P. tricornutum* was strongly upregulated when exposed to a constant 48-h HL, whereas *lhcx3*, *lhcr6*, and *lhcr8* were strongly upregulated after0.5h of HL but slightly decreased during the last 47.5 h of HL (Nymark et al., 2009). It suggested a photoprotective role followed by acclimation to HL for *lhcx3* and for *lhcr6* and *lhcr8*. According to our BLAST results, the corresponding Lhcr6 and Lhcr8 proteins in *P. tricornutum* had the highest similarity scores with Lhcr5 and Lhcr6 in *T. lutea*, respectively. The corresponding *lhcr6* gene in *T. lutea* was the only *lhcr* significantly upregulated at 8:00 h vs. 12:00 h, along with *lhcf21*. Other *lhcr* were not upregulated in HL either in *T. lutea* or in *P. tricornutum* (Nymark et al., 2009). The Lhcr6 protein in *T. lutea*, in addition to Lhcf21 and Lhcx, could therefore participate in binding of photoprotective pigments or play a structural role in FCP conformation for photoprotection under HL, like Lhcx1 in *T. pseudonana* (Zhu and Green, 2010; Ghazaryan et al., 2016). Furthermore, Lhcr6 and Lhcr10 could also be classified as Lhcz (Supplementary Table 7), which might highlight the potential photoprotective function of Lhcz. Indeed, although *lhcr10* was not significantly upregulated at 8:00 h, its *p*-value of0.0512 was close to the0.05 threshold.

In *T. lutea*, *lhcx2* was the only gene upregulated at night. It is known that the Lhcx4 protein in *P. tricornutum* is also more synthesized at night (Lepetit et al., 2013; Nymark et al., 2013; Taddei et al., 2016; Buck et al., 2019). Furthermore, in the transcriptomics analysis, *lhcx4* was barely expressed under HL (Nymark et al., 2009), but there was no clear explanation about its function yet. A hypothesis is that Lhcx4 could protect from the return of light after prolonged darkness exposure. We can suppose that the Lhcx2 protein in *T. lutea* plays the same role.

## Conclusion

We characterized the FCP of *Tisochrysis lutea* for the first time in three ways. Initially, 52 *lhc* genes were annotated. We classified the genes into 3 families, *lhcf*, *lhcr*, and *lhcx*, as for diatoms. We also identified the Lhcz subgroup within the Lhcr clade of *T. lutea*. Furthermore, we did not exclude the existence of a novel group found in diatoms named Lhcq, whose function is unknown so far. Then, we evaluated the light-harvesting capacities of *T. lutea* by locating pigment-binding domains in Lhcf protein monomers, which are likely to be homologous to those of *P. tricornutum*. Finally, we investigated the expression of *lhc* genes. The study on dynamic photoperiodic cycle and dynamic N intake within the culture of *T. lutea* demonstrated a very sensitive dynamics of both photosynthetic and photoprotective pigments, along with clear difference in *lhc* gene expression.

## Data Availability Statement

The original contributions presented in the study are publicly available. This data can be found here: National Center for Biotechnology Information (NCBI) BioProject database under accession number PRJNA787725.

## Author Contributions

AP wrote the article, analyzed the pigment data and raw RNA-seq transcripts, and interpreted all the data of this study. EN and JL contributed greatly to the writing, ordering, and correction of Introduction, Results, and Discussion, and to the design of the graphics. EN analyzed the majority of the pigment data. GC, BS-J, J-BB, CB, and MG designed, planned, realized, and analyzed the experiments. OB designed and analyzed the DL experiments and contributed to its funding. NR contributed to the creation of the R process for RNA-seq analysis. LM as thesis director of AP, contributed in supervising the writing of the article. All authors contributed to the article and approved the submitted version.

## Conflict of Interest

The authors declare that the research was conducted in the absence of any commercial or financial relationships that could be construed as a potential conflict of interest.

## Publisher’s Note

All claims expressed in this article are solely those of the authors and do not necessarily represent those of their affiliated organizations, or those of the publisher, the editors and the reviewers. Any product that may be evaluated in this article, or claim that may be made by its manufacturer, is not guaranteed or endorsed by the publisher.

## References

[B1] Akimoto S. Teshigahara A. Yokono M. Mimuro M. Nagao R. Tomo T. (2014). Excitation relaxation dynamics and energy transfer in fucoxanthin–chlorophyll a/c-protein complexes, probed by time-resolved fluorescence. *Biochim. Biophys. Acta* 1837 1514–1521. 10.1016/j.bbabio.2014.02.002 24530875

[B2] Anning T. MacIntyre H. L. Pratt S. M. Sammes P. J. Gibb S. Geider R. J. (2000). Photoacclimation in the marine diatom *Skeletonemacostatum*. *Limnol. Oceanogr.* 45 1807–1817. 10.4319/lo.2000.45.8.1807

[B3] Apt K. E. Bhaya D. Grossman A. R. (1994). Characterization of genes encoding the light-harvesting proteins in diatoms: biogenesis of the fucoxanthin chlorophylla/c protein complex. *J. Appl. Phycol.* 6 225–230. 10.1007/BF02186075

[B4] Apt K. E. Clendennen S. K. Powers D. A. Grossman A. R. (1995). The gene family encoding the fucoxanthin chlorophyll proteins from the brown alga *Macrocystispyrifera*. *Mol. Gen. Genet.* 246 455–464. 10.1007/BF00290449 7891659

[B5] Arora N. Philippidis G. P. (2021). “Fucoxanthin Production from Diatoms: current Advances and Challenges,” in *Algae: multifarious Applications for a Sustainable World*, eds Mandotra S. K. Upadhyay A. K. Ahluwalia A. S. (Singapore: Springer), 227–242. 10.1007/978-981-15-7518-1_10

[B6] Bailleul B. Rogato A. de Martino A. Coesel S. Cardol P. Bowler C. (2010). An atypical member of the light-harvesting complex stress-related protein family modulates diatom responses to light. *Proc. Natl. Acad. Sci. U. S. A.* 107 18214–18219. 10.1073/pnas.1007703107 20921421 PMC2964204

[B7] Beer A. Gundermann K. Beckmann J. Büchel C. (2006). Subunit Composition and Pigmentation of Fucoxanthin-Chlorophyll Proteins in Diatoms: Evidence for a Subunit Involved in Diadinoxanthin and Diatoxanthin Binding. *Biochemistry* 45 13046–13053. 10.1021/bi061249h 17059221

[B8] Berthelier J. Casse N. Daccord N. Jamilloux V. Saint-Jean B. Carrier G. (2018). *Annotation of the genome assembly (version 2) of the microalga Tisochrysis lutea.* Brest: SEANOE. 10.17882/52231 PMC5963040 29783941

[B9] Bhaya D. Grossman A. R. (1993). Characterization of gene clusters encoding the fucoxanthin chlorophyll proteins of the diatom *Phaeodactylum tricornutum*. *Nucleic Acids Res.* 21 4458–4466. 10.1093/nar/21.19.4458 8233779 PMC311176

[B10] Blommaert L. Huysman M. J. J. Vyverman W. Lavaud J. Sabbe K. (2017). Contrasting NPQ dynamics and xanthophyll cycling in a motile and a non-motile intertidal benthic diatom. *Limnol. Oceanogr.* 62 1466–1479. 10.1002/lno.10511

[B11] Blommaert L. Vancaester E. Huysman M. J. J. Osuna-Cruz C. M. D’hondt S. Lavaud J. (2020). Light Regulation of LHCX Genes in the Benthic Diatom *Seminavisrobusta*. *Front. Mar. Sci.* 7:192. 10.3389/fmars.2020.00192

[B12] Brown B. E. Ambarsari I. Warner M. E. Fitt W. K. Dunne R. P. Gibb S. W. (1999). Diurnal changes in photochemical efficiency and xanthophyll concentrations in shallow water reef corals: evidence for photoinhibition and photoprotection. *Coral Reefs* 18 99–105. 10.1007/s003380050163

[B13] Brown C. W. Yoder J. A. (1993). Blooms of *Emiliania huxleyi* (*Prymnesiophyceae*) in surface waters of the Nova Scotian Shelf and the Grand Bank. *J. Plankton Res.* 15 1429–1438. 10.1093/plankt/15.12.1429

[B14] Büchel C. (2020b). “Light-Harvesting Complexes of Diatoms: fucoxanthin-Chlorophyll Proteins,” in *Photosynthesis in Algae: biochemical and Physiological Mechanisms Advances in Photosynthesis and Respiration*, eds Larkum A. W. D. Grossman A. R. Raven J. A. (Cham: Springer International Publishing), 441–457. 10.1007/978-3-030-33397-3_16

[B15] Büchel C. (2020a). Light harvesting complexes in chlorophyll c-containing algae. *Biochim. Biophys. Acta* 1861:148027. 10.1016/j.bbabio.2019.05.003 31153887

[B16] Buck J. M. Kroth P. G. Lepetit B. (2021). Identification of sequence motifs in Lhcx proteins that confer qE-based photoprotection in the diatom *Phaeodactylum tricornutum*. *Plant J.* 108 1721–1734. 10.1111/tpj.15539 34651379

[B17] Buck J. M. Sherman J. Bártulos C. R. Serif M. Halder M. Henkel J. (2019). Lhcx proteins provide photoprotection via thermal dissipation of absorbed light in the diatom *Phaeodactylum tricornutum*. *Nat. Commun.* 10:4167. 10.1038/s41467-019-12043-6 31519883 PMC6744471

[B18] Carrier G. Garnier M. Le Cunff L. Bougaran G. Probert I. De Vargas C. (2014). Comparative Transcriptome of Wild Type and Selected Strains of the Microalgae *Tisochrysis lutea* Provides Insights into the Genetic Basis, Lipid Metabolism and the Life Cycle. *PLoS One* 9:e86889. 10.1371/journal.pone.0086889 24489800 PMC3906074

[B19] Cavalier-Smith T. (2004). “Chromalveolate diversity and cell megaevolution: interplay of membranes, genomes and cytoskeleton,” in *Organelles, Genomes and Eukaryote Phylogeny: an Evolutionary Synthesis in the Age of Genomics*, eds Hirt R. P. Horner D. S. (London: CRC Press), 71–103.

[B20] Cezare-Gomes E. A. Mejia-da-Silva L. C. Pérez-Mora L. S. Matsudo M. C. Ferreira-Camargo L. S. Singh A. K. (2019). Potential of Microalgae Carotenoids for Industrial Application. *Appl. Biochem. Biotechnol.* 188 602–634. 10.1007/s12010-018-02945-4 30613862

[B21] Cortés M. Y. Bollmann J. Thierstein H. R. (2001). Coccolithophore ecology at the HOT station ALOHA, Hawaii. *Deep Sea Res. II Top. Stud. Oceanogr.* 48 1957–1981. 10.1016/S0967-0645(00)00165-X

[B22] Custódio L. Soares F. Pereira H. Barreira L. Vizetto-Duarte C. Rodrigues M. J. (2014). Fatty acid composition and biological activities of *Isochrysis galbana* T-ISO, *Tetraselmis sp.* and *Scenedesmus sp.*: possible application in the pharmaceutical and functional food industries. *J. Appl. Phycol.* 26 151–161. 10.1007/s10811-013-0098-0

[B23] Dahmen-Ben Moussa I. Chtourou H. Karray F. Sayadi S. Dhouib A. (2017). Nitrogen or phosphorus repletion strategies for enhancing lipid or carotenoid production from *Tetraselmis marina*. *Bioresour. Technol.* 238 325–332. 10.1016/j.biortech.2017.04.008 28456040

[B24] Dambek M. Eilers U. Breitenbach J. Steiger S. Büchel C. Sandmann G. (2012). Biosynthesis of fucoxanthin and diadinoxanthin and function of initial pathway genes in *Phaeodactylum tricornutum*. *J. Exp. Bot.* 63 5607–5612. 10.1093/jxb/ers211 22888128 PMC3444273

[B25] Dautermann O. Lyska D. Andersen-Ranberg J. Becker M. Fröhlich-Nowoisky J. Gartmann H. (2020). An algal enzyme required for biosynthesis of the most abundant marine carotenoids. *Sci. Adv.* 6:eaaw9183. 10.1126/sciadv.aaw9183 32181334 PMC7056318

[B26] Delaunay F. Marty Y. Moal J. Samain J.-F. (1993). The effect of monospecific algal diets on growth and fatty acid composition of *Pecten maximus* (L.) larvae. *J. Exp. Mar. Biol. Ecol.* 173 163–179. 10.1016/0022-0981(93)90051-O

[B27] Delbrut A. Albina P. Lapierre T. Pradelles R. Dubreucq E. (2018). Fucoxanthin and Polyunsaturated Fatty Acids Co-Extraction by a Green Process. *Molecules* 23:874. 10.3390/molecules23040874 29641444 PMC6017215

[B28] Demmig-Adams B. Garab G. Adams W. W. Govindjee (eds) (2014). *Non-Photochemical Quenching and Energy Dissipation in Plants, Algae and Cyanobacteria.* Dordrecht: Springer Netherlands, 10.1007/978-94-017-9032-1

[B29] Di Valentin M. Büchel C. Giacometti G. M. Carbonera D. (2012). Chlorophyll triplet quenching by fucoxanthin in the fucoxanthin–chlorophyll protein from the diatom *Cyclotella meneghiniana*. *Biochem. Biophys. Res. Commun.* 427 637–641. 10.1016/j.bbrc.2012.09.113 23026044

[B30] Duarte B. Feijão E. Goessling J. W. Caçador I. Matos A. R. (2021). Pigment and Fatty Acid Production under Different Light Qualities in the Diatom *Phaeodactylum tricornutum*. *Appl. Sci.* 11:2550. 10.3390/app11062550

[B31] Dubinsky Z. Falkowski P. G. Wyman K. (1986). Light Harvesting and Utilization by Phytoplankton. *Plant Cell Physiol.* 27 1335–1349. 10.1093/oxfordjournals.pcp.a077232

[B32] Edvardsen B. Egge E. S. Vaulot D. (2016). Diversity and distribution of haptophytes revealed by environmental sequencing and metabarcoding – a review. *Perspect. Phycol.* 3 77–91. 10.1127/pip/2016/0052

[B33] Falkowski P. G. Sukenik A. Herzig R. (1989). Nitrogen limitation in *Isochrysis Galbana* (haptophyceae). II. relative abundance of chloroplast proteins. *J. Phycol*. 25, 471–478. 10.1111/j.1529-8817.1989.tb00252.x

[B34] Field C. B. Behrenfeld M. J. Randerson J. T. Falkowski P. (1998). Primary Production of the Biosphere: integrating Terrestrial and Oceanic Components. *Science* 281 237–240. 10.1126/science.281.5374.237 9657713

[B35] Gao F. Teles I. Wijffels R. H. Barbosa M. J. (2020a). Process optimization of fucoxanthin production with *Tisochrysis lutea*. *Bioresour. Technol.* 315:123894. 10.1016/j.biortech.2020.123894 32736321

[B36] Gao F. Teles L. Ferrer-Ledo N. Wijffels R. H. Barbosa M. J. (2020b). Production and high throughput quantification of fucoxanthin and lipids in *Tisochrysis lutea* using single-cell fluorescence. *Bioresour. Technol.* 318:124104. 10.1016/j.biortech.2020.124104 32942095

[B37] Garnier M. Bougaran G. Pavlovic M. Berard J.-B. Carrier G. Charrier A. (2016). Use of a lipid rich strain reveals mechanisms of nitrogen limitation and carbon partitioning in the haptophyte *Tisochrysis lutea*. *Algal Res.* 20 229–248. 10.1016/j.algal.2016.10.017

[B38] Geider R. Macintyre H. Graziano L. McKay R. M. (1998). Responses of the photosynthetic apparatus of *Dunaliellatertiolecta*(*Chlorophyceae*) to nitrogen and phosphorus limitation. *Eur. J. Phycol.* 33 315–332. 10.1080/09670269810001736813

[B39] Gelzinis A. Butkus V. Songaila E. Augulis R. Gall A. Büchel C. (2015). Mapping energy transfer channels in fucoxanthin–chlorophyll protein complex. *Biochim. Biophys. Acta* 1847 241–247. 10.1016/j.bbabio.2014.11.004 25445318

[B40] Ghazaryan A. Akhtar P. Garab G. Lambrev P. H. Büchel C. (2016). Involvement of the Lhcx protein Fcp6 of the diatom *Cyclotella meneghiniana*in the macro-organisation and structural flexibility of thylakoid membranes. *Biochim. Biophys. Acta* 1857 1373–1379. 10.1016/j.bbabio.2016.04.288 27155390

[B41] Goss R. Lepetit B. (2015). Biodiversity of NPQ. *J. Plant Physiol.* 172 13–32. 10.1016/j.jplph.2014.03.004 24854581

[B42] Green J. C. Jordan R. W. (2000). “Order Prymnesiida,” in *Illustrated Guide to the Protozoa*, eds Lee J. J. Leedale G. F. Bradbury P. (Lawrence, KA: Society of Protozoologists), 1268–1301.

[B43] Gundermann K. Büchel C. (2008). The fluorescence yield of the trimeric fucoxanthin–chlorophyll–protein FCPa in the diatom *Cyclotella meneghiniana*is dependent on the amount of bound diatoxanthin. *Photosynth. Res.* 95 229–235. 10.1007/s11120-007-9262-1 17912602

[B44] Gundermann K. Schmidt M. Weisheit W. Mittag M. Büchel C. (2013). Identification of several sub-populations in the pool of light harvesting proteins in the pennate diatom *Phaeodactylum tricornutum*. *Biochim. Biophys. Acta* 1827 303–310. 10.1016/j.bbabio.2012.10.017 23142526

[B45] Harper J. T. Waanders E. Keeling P. J. (2005). On the monophyly of chromalveolates using a six-protein phylogeny of eukaryotes. *Int. J. Syst. Evol. Microbiol.* 55 487–496. 10.1099/ijs.0.63216-0 15653923

[B46] Jensen E. Clément R. Maberly S. C. Gontero B. (2017). Regulation of the Calvin–Benson–Bassham cycle in the enigmatic diatoms: biochemical and evolutionary variations on an original theme. *Philos. Trans. R. Soc. B Biol. Sci.* 372:20160401. 10.1098/rstb.2016.0401 28717027 PMC5516110

[B47] Jordan R. W. Chamberlain A. H. L. (1997). Biodiversity among haptophyte algae. *Biodivers. Conserv.* 6 131–152. 10.1023/A:1018383817777

[B48] Koziol A. G. Borza T. Ishida K.-I. Keeling P. Lee R. W. Durnford D. G. (2007). Tracing the Evolution of the Light-Harvesting Antennae in Chlorophyll a/b-Containing Organisms. *Plant Physiol.* 143 1802–1816. 10.1104/pp.106.092536 17307901 PMC1851817

[B49] Kuczynska P. Jemiola-Rzeminska M. Strzalka K. (2015). Photosynthetic Pigments in Diatoms. *Mar. Drugs* 13 5847–5881. 10.3390/md13095847 26389924 PMC4584358

[B50] Kumazawa M. Nishide H. Nagao R. Inoue-Kashino N. Shen J.-R. Nakano T. (2021). Molecular phylogeny of fucoxanthin-chlorophyll a/c proteins from Chaetocerosgracilis and Lhcq/Lhcf diversity. *Physiol. Plant.* [Epub Online ahead of print]. 10.1111/ppl.13598 34792189

[B51] Lacour T. Babin M. Lavaud J. (2020). Diversity in Xanthophyll Cycle Pigments Content and Related Nonphotochemical Quenching (NPQ) Among Microalgae: implications for Growth Strategy and Ecology. *J. Phycol.* 56 245–263. 10.1111/jpy.12944 31674660

[B52] Lavaud J. Rousseau B. Etienne A.-L. (2004). General Features of Photoprotection by Energy Dissipation in Planktonic Diatoms (*bacillariophyceae*)1. *J. Phycol.* 40 130–137. 10.1046/j.1529-8817.2004.03026.x

[B53] Lavaud J. Rousseau B. van Gorkom H. J. Etienne A.-L. (2002). Influence of the Diadinoxanthin Pool Size on Photoprotection in the Marine Planktonic Diatom *Phaeodactylum tricornutum*. *Plant Physiol.* 129 1398–1406. 10.1104/pp.002014 12114593 PMC166533

[B54] Leal E. de Beyer L. O’Connor W. Dove M. Ralph P. J. Pernice M. (2020). Production optimisation of *Tisochrysis lutea* as a live feed for juvenile Sydney rock oysters, *Saccostrea glomerata*, using large-scale photobioreactors. *Aquaculture* 533:736077. 10.1016/j.aquaculture.2020.736077

[B55] Lefebvre S. C. Harris G. Webster R. Leonardos N. Geider R. J. Raines C. A. (2010). Characterization and expression analysis of the Lhcf gene family in *Emiliania huxleyi* (*haptophyta*) reveals differential responses to light and CO2. *J. Phycol.* 46 123–134. 10.1111/j.1529-8817.2009.00793.x

[B56] Lepetit B. Gélin G. Lepetit M. Sturm S. Vugrinec S. Rogato A. (2017). The diatom *Phaeodactylum tricornutum* adjusts nonphotochemical fluorescence quenching capacity in response to dynamic light via fine-tuned Lhcx and xanthophyll cycle pigment synthesis. *New Phytol.* 214 205–218. 10.1111/nph.14337 27870063

[B57] Lepetit B. Sturm S. Rogato A. Gruber A. Sachse M. Falciatore A. (2013). High Light Acclimation in the Secondary Plastids Containing Diatom *Phaeodactylum tricornutum* is Triggered by the Redox State of the Plastoquinone Pool. *Plant Physiol.* 161 853–865. 10.1104/pp.112.207811 23209128 PMC3561024

[B58] Liu H. Probert I. Uitz J. Claustre H. Aris-Brosou S. Frada M. (2009). Extreme diversity in noncalcifying haptophytes explains a major pigment paradox in open oceans. *Proc. Natl. Acad. Sci. U. S. A.* 106 12803–12808. 10.1073/pnas.0905841106 19622724 PMC2722306

[B59] Longworth J. Wu D. Huete-Ortega M. Wright P. C. Vaidyanathan S. (2016). Proteome response of *Phaeodactylum tricornutum*, during lipid accumulation induced by nitrogen depletion. *Algal Res.* 18, 213–224. 10.1016/j.algal.2016.06.015 27812494 PMC5070409

[B60] Marchetti J. Bougaran G. Jauffrais T. Lefebvre S. Rouxel C. Saint-Jean B. (2013). Effects of blue light on the biochemical composition and photosynthetic activity of *Isochrysis sp.*(T-iso). *J. Appl. Phycol.* 25 109–119. 10.1007/s10811-012-9844-y

[B61] Matos J. Cardoso C. Gomes A. Campos A. M. Falé P. Afonso C. (2019). Bioprospection of *Isochrysis galbana* and its potential as a nutraceutical. *Food Funct.* 10:7333. 10.1039/c9fo01364d 31646314

[B62] McClure D. D. Luiz A. Gerber B. Barton G. W. Kavanagh J. M. (2018). An investigation into the effect of culture conditions on fucoxanthin production using the marine microalgae *Phaeodactylum tricornutum*. *Algal Res.* 29 41–48. 10.1016/j.algal.2017.11.015

[B63] McKew B. A. Lefebvre S. C. Achterberg E. P. Metodieva G. Raines C. A. Metodiev M. V. (2013). Plasticity in the proteome of *Emiliania huxleyi* CCMP 1516 to extremes of light is highly targeted. *New Phytol.* 200 61–73. 10.1111/nph.12352 23750769

[B64] Mohamadnia S. Tavakoli O. Faramarzi M. A. Shamsollahi Z. (2019). Production of fucoxanthin by the microalga *Tisochrysis lutea*: a review of recent developments. *Aquaculture* 516:734637. 10.1016/j.aquaculture.2019.734637

[B65] Nagao R. Kato K. Ifuku K. Suzuki T. Kumazawa M. Uchiyama I. (2020). Structural basis for assembly and function of a diatom photosystem I-light-harvesting supercomplex. *Nat. Commun.* 11:2481. 10.1038/s41467-020-16324-3 32424145 PMC7235021

[B66] Nagao R. Kato K. Suzuki T. Ifuku K. Uchiyama I. Kashino Y. (2019a). Structural basis for energy harvesting and dissipation in a diatom PSII–FCPII supercomplex. *Nat. Plants* 5 890–901. 10.1038/s41477-019-0477-x 31358960

[B67] Nagao R. Ueno Y. Yokono M. Shen J.-R. Akimoto S. (2019b). Effects of excess light energy on excitation-energy dynamics in a pennate diatom *Phaeodactylum tricornutum*. *Photosynth. Res.* 141 355–365. 10.1007/s11120-019-00639-4 30993504

[B68] Nagao R. Yokono M. Akimoto S. Tomo T. (2013). High Excitation Energy Quenching in Fucoxanthin Chlorophyll a/c-Binding Protein Complexes from the Diatom Chaetocerosgracilis. *J. Phys. Chem. B* 117 6888–6895. 10.1021/jp403923q 23688343

[B69] Nymark M. Valle K. C. Brembu T. Hancke K. Winge P. Andresen K. (2009). An Integrated Analysis of Molecular Acclimation to High Light in the Marine Diatom *Phaeodactylum tricornutum*. *PLoS One* 4:e7743. 10.1371/journal.pone.0007743 19888450 PMC2766053

[B70] Nymark M. Valle K. C. Hancke K. Winge P. Andresen K. Johnsen G. (2013). Molecular and Photosynthetic Responses to Prolonged Darkness and Subsequent Acclimation to Re-Illumination in the Diatom *Phaeodactylum tricornutum*. *PLoS One* 8:e58722. 10.1371/journal.pone.0058722 23520530 PMC3592843

[B71] Oeltjen A. Krumbein W. E. Rhiel E. (2002). Investigations on Transcript Sizes, Steady State mRNA Concentrations and Diurnal Expression of Genes Encoding Fucoxanthin Chlorophyll a/c Light Harvesting Polypeptides in the Centric Diatom *Cyclotella cryptica*. *Plant Biol.* 4 250–257. 10.1055/s-2002-25737

[B72] Papagiannakis E. Van Stokkum I. H. M. Fey H. Büchel C. Van Grondelle R. (2005). Spectroscopic Characterization of the Excitation Energy Transfer in the Fucoxanthin–Chlorophyll Protein of Diatoms. *Photosynth. Res.* 86 241–250. 10.1007/s11120-005-1003-8 16172942

[B73] Patron N. J. Inagaki Y. Keeling P. J. (2007). Multiple Gene Phylogenies Support the Monophyly of Cryptomonad and Haptophyte Host Lineages. *Curr. Biol.* 17 887–891. 10.1016/j.cub.2007.03.069 17462896

[B74] Perrin L. (2017). *Physiologie du coccolithophoridé Emiliania huxleyi en co-limitation de nutriments et de lumière.* Ph.D. thesis. Paris: Université Pierre et Marie Curie

[B75] Pi X. Zhao S. Wang W. Liu D. Xu C. Han G. (2019). The pigment-protein network of a diatom photosystem II–light-harvesting antenna supercomplex. *Science* 365:eaax4406. 10.1126/science.aax4406 31371578

[B76] Premvardhan L. Bordes L. Beer A. Büchel C. Robert B. (2009). Carotenoid Structures and Environments in Trimeric and Oligomeric Fucoxanthin Chlorophyll a/c2 Proteins from Resonance Raman Spectroscopy. *J. Phys. Chem. B* 113 12565–12574. 10.1021/jp903029g 19697894

[B77] Rico-Villa B. Le Coz J. R. Mingant C. Robert R. (2006). Influence of phytoplankton diet mixtures on microalgae consumption, larval development and settlement of the Pacific oyster *Crassostrea gigas* (Thunberg). *Aquaculture* 256 377–388. 10.1016/j.aquaculture.2006.02.015

[B78] Sonenberg N. Hinnebusch A. G. (2009). Regulation of translation initiation in eukaryotes: mechanisms and biological targets. *Cell* 136 731–745. 10.1016/j.cell.2009.01.042 19239892 PMC3610329

[B79] Suggett D. J. Le Floc’H E. Harris G. N. Leonardos N. Geider R. J. (2007). Different strategies of photoacclimation by two strains of *Emiliania huxleyi* (*Haptophyta*). *J. Phycol.* 43 1209–1222. 10.1111/j.1529-8817.2007.00406.x

[B80] Taddei L. Stella G. R. Rogato A. Bailleul B. Fortunato A. E. Annunziata R. (2016). Multisignal control of expression of the LHCX protein family in the marine diatom *Phaeodactylum tricornutum*. *J. Exp. Bot.* 67 3939–3951. 10.1093/jxb/erw198 27225826 PMC4915529

[B81] Van Heukelem L. Thomas C. S. (2001). Computer-assisted high-performance liquid chromatography method development with applications to the isolation and analysis of phytoplankton pigments. *J. Chromatogr. A* 910 31–49. 10.1016/S0378-4347(00)00603-4 11263574

[B82] Walne P. R. (1966). *Experiments in the Large-scale Culture of the Larvae of Ostrea Edulis L.* Richmond, London: H.M. Stationery Office.

[B83] Wang W. Yu L.-J. Xu C. Tomizaki T. Zhao S. Umena Y. (2019a). Structural basis for blue-green light harvesting and energy dissipation in diatoms. *Science* 363:eaav0365. 10.1126/science.aav0365 30733387

[B84] Wang W. Zhao S. Pi X. Kuang T. Sui S.-F. Shen J.-R. (2019b). Structural features of the diatom photosystem II–light-harvesting antenna complex. *FEBS J.* 287 2191–2200. 10.1111/febs.15183 31854056

[B85] Wever A. D. Muylaert K. Langlet D. Alleman L. Descy J.-P. André L. (2008). Differential response of phytoplankton to additions of nitrogen, phosphorus and iron in Lake Tanganyika. *Freshw. Biol.* 53 264–277. 10.1111/j.1365-2427.2007.01890.x

[B86] Xia S. Gao B. Fu J. Xiong J. Zhang C. (2018). Production of fucoxanthin, chrysolaminarin, and eicosapentaenoic acid by *Odontellaaurita* under different nitrogen supply regimes. *J. Biosci. Bioeng.* 126 723–729. 10.1016/j.jbiosc.2018.06.002 29958771

[B87] Xu C. Pi X. Huang Y. Han G. Chen X. Qin X. (2020). Structural basis for energy transfer in a huge diatom PSI-FCPI supercomplex. *Nat. Commun.* 11:5081. 10.1038/s41467-020-18867-x 33033236 PMC7545214

[B88] Yang R. Wei D. (2020). Improving Fucoxanthin Production in Mixotrophic Culture of Marine Diatom *Phaeodactylum tricornutum* by LED Light Shift and Nitrogen Supplementation. *Front. Bioeng. Biotechnol.* 8:820. 10.3389/fbioe.2020.00820 32760713 PMC7373720

[B89] Zarrinmehr M. J. Farhadian O. Heyrati F. P. Keramat J. Koutra E. Kornaros M. (2019). Effect of nitrogen concentration on the growth rate and biochemical composition of the microalga, *Isochrysis galbana*. *Egypt. J. Aquat. Res.* 46 153–158. 10.1016/j.ejar.2019.11.003

[B90] Zhu S.-H. Green B. R. (2010). Photoprotection in the diatom *Thalassiosira pseudonana*: role of LI818-like proteins in response to high light stress. *Biochim. Biophys. Acta* 1797 1449–1457. 10.1016/j.bbabio.2010.04.003 20388491

