# Supplementary material for: The Fucoxanthin Chlorophyll a/c-Binding Protein in Tisochrysis lutea: Influence of Nitrogen and Light on Fucoxanthin and Chlorophyll a/c-Binding Protein Gene Expression and Fucoxanthin Synthesis
Source: Front Plant Sci. 2022 Feb 17;13:830069. doi: 10.3389/fpls.2022.830069 (PMC8891753; doi:10.3389/fpls.2022.830069)

***Frontiers in Plant Science-Plant Biotechnology* Supplementary material**

The following supplementary material is available for this article:

Fig. S1 Cladogram of the FCP sequences of *E. huxleyi*

Fig. S2 Cladogram of the Lhcr sequences of *E. huxleyi* and *T. lutea*

Fig. S3 Binding sites of Chl *a*, Chl *c* and Fx in *T. lutea*

Fig. S4 Evolution of intracellular N, of Fx/C and of Chl *a*/C ratio in dynamic light experiment (day:night cycle and NO_3 variation)

Fig. S5 MA plot between N limitation and N repletion in constant light experiment (NO_3 variation)

Fig. S6 MA plot between N limitation and N depletion in constant light experiment

Fig. S7 MA plot between N repletion and N depletion in constant light experiment

Fig. S8 MA plot between 8:00 h and 12:00 h in dynamic light experiment

Fig. S9 MA plot between 8:00 h and 00:00 h in dynamic light experiment

Fig. S10 MA plot between 12:00 h and 00:00 h in dynamic light experiment

Table S1 Analysis of FCP sequences available in Chromista compared with the 35 sequences of *Phaeodactylum tricornutum*

Table S2 List of the FCP sequences of *P. tricornutum*, *F. cylindrus,* and *T. pseudonana* used for BLAST comparison

Table S3 BLAST comparison between the FCP of *T. lutea* and *P. tricornutum*

Table S4 BLAST comparison between the FCP of *T. lutea* and *F. cylindrus*

Table S5 BLAST comparison between the FCP of *T. lutea* and *T. pseudonana*

Table S6 List of the FCP sequences of four other species used for the construction of the Lhcf cladogram

Table S7 FCP sequences in *T. lutea*

Table S8 Expression rate of *lhcf* genes in constant light experiment

Table S9 Expression rate of *lhcr* genes in constant light experiment

Table S10 Expression rate of *lhcx* genes in constant light experiment

Table S11 Expression rate of *lhcf* genes in dynamic light experiment, replicate 1

Table S12 Expression rate of *lhcf* genes in dynamic light experiment, replicate 2

Table S13 Expression rate of *lhcr* genes in dynamic light experiment, replicate 1

Table S14 Expression rate of *lhcr* genes in dynamic light experiment, replicate 2

Table S15 Expression rate of *lhcx* genes in dynamic light experiment, replicate 1

Table S16 Expression rate of *lhcx* genes in dynamic light experiment, replicate 2

Table S17 p-value of the differential expression of FCP genes in constant light experiment

Table S18 p-value of the differential expression of FCP genes in dynamic light experiment (8:00 h and 12:00 h VS 00:00 h)

Table S19 p-value of the differential expression of FCP genes in dynamic light experiment (8:00 h VS 12:00 h)

Table S1 Comparative analysis of the 35 FCP sequences of Phaeodactylum tricornutum to all protein sequences available in Chromista.

	**Comparison of the 35 FCP sequences of *Phaeodactylum tricornutum***		
	**Species**	**Identity**	**Alignment cover**
With diatoms	*Fistulifera solaris, Fragiliaropsis cylindrus, pseudo-Nitzschia multistriata, Thalassosira pseudonana, Thalassosira oceanica, Cyclotella cryptica, Cylindrotheca fusiformis, Skeletonema costatum, Chaetoceros tenuissimus, Chaetoceros gracilis, Nitzschia inconspicua*	62.96%	70-98%
With haptophytes	*Emiliania huxleyi, Chrysochromulina tobinii, Isochrysis galbana, Pavlova lutheri, Chrysotila carterae, Diacronema lutheri*	46.88%	30-60%
With ocrophytes	*Aureococcus anophagefferens, Nannochloropsis gaditana, Heterosigma akashiwo, Pelagomonas calceolata*	46.56%	<50%
With dinoflagellates	*Heterocapsa triquetra, Symbiodinium microadriaticum, Durinskia baltica, Polarella glacialis*	55.25%	<50%
With brown seaweeds	*Ectocarpus silicosus, Saccharina latissima, Saccharina japonica*	45.06%	<50%
With alveolate	*Vitrella brassicaformis*	47%	>88%

Table S2 FCP protein sequences of *P. tricornutum*, *T. pseudonana* and *F. cylindrus* used for BLAST comparison and FCP identification in *T. lutea*

FCP sequences of diatoms species used for BLAST (UniprotKB)					
Organism	Strain	Entry	Protein family	Protein name	Length
Phaeodactylum tricornutum	CCAP 1055/1	B7FRW5	Protein fucoxanthin chlorophyll a/c protein	Lhcf1 PHATRDRAFT_18049	196
Phaeodactylum tricornutum	CCAP 1055/1	B7FRW4	Protein fucoxanthin chlorophyll a/c protein	Lhcf2 PHATRDRAFT_25172	198
Phaeodactylum tricornutum	CCAP 1055/1	B7FRW2	Protein fucoxanthin chlorophyl a/c protein	Lhcf4 Lhcf3 PHATRDRAFT_25168 PHATRDRAFT_50705	198
Phaeodactylum tricornutum	CCAP 1055/1	B7GBK7	Protein fucoxanthin chlorophyll a/c protein	Lhcf5 PHATRDRAFT_30648	197
Phaeodactylum tricornutum	CCAP 1055/1	B7G5S7	Protein fucoxanthin chlorophyll a/c protein	Lhcf6 Lhcf7 PHATRDRAFT_29266 PHATRDRAFT_30643	204
Phaeodactylum tricornutum	CCAP 1055/1	B7G6Y1	Protein fucoxanthin chlorophyll a/c protein	Lhcf8 PHATRDRAFT_22395	200
Phaeodactylum tricornutum	CCAP 1055/1	B7G955	Protein fucoxanthin chlorophyll a/c protein	Lhcf9 PHATRDRAFT_30031	205
Phaeodactylum tricornutum	CCAP 1055/1	B7G5B6	Protein fucoxanthin chlorophyll a/c protein	Lhcf10 PHATRDRAFT_22006	199
Phaeodactylum tricornutum	CCAP 1055/1	B7GBK6	Protein fucoxanthin chlorophyll a/c protein	Lhcf11 PHATRDRAFT_51230	197
Phaeodactylum tricornutum	CCAP 1055/1	B7GCA2	Protein fucoxanthin chlorophyll protein	Lhcf12 PHATRDRAFT_16302	193

Phaeodactylum tricornutum	CCAP 1055/1	B7G871	Protein fucoxanthin chlorophyll a/c protein	Lhcf13 PHATRDRAFT_22680	197
Phaeodactylum tricornutum	CCAP 1055/1	B5Y5L4	Fucoxanthin chlorophyll a/c protein, lhcf type	Lhcf14 PHATR_25893	195
Phaeodactylum tricornutum	CCAP 1055/1	B7G8Q1	Protein fucoxanthin chlorophyll a/c protein	Lhcf15 PHATRDRAFT_48882	203
Phaeodactylum tricornutum	CCAP 1055/1	B7FV42	Protein fucoxanthin chlorophyll a/c protein	Lhcf16 PHATRDRAFT_34536	206
Phaeodactylum tricornutum	CCAP 1055/1	B7GC99	Protein fucoxanthin chlorophyll a/c protein	Lhcf17 PHATRDRAFT_56310	206
Phaeodactylum tricornutum	CCAP 1055/1	B7FUM6	Protein fucoxanthin chlorophyll a/c protein	Lhcr1 PHATRDRAFT_44601	200
Phaeodactylum tricornutum	CCAP 1055/1	B7G9B9	Protein fucoxanthin chlorophyll a/c protein	Lhcr2 PHATRDRAFT_22956	216
Phaeodactylum tricornutum	CCAP 1055/1	B7FSP4	Protein fucoxanthin chlorophyll a/c protein	Lhcr3 PHATRDRAFT_50725	199
Phaeodactylum tricornutum	CCAP 1055/1	B7FQE1	Protein fucoxanthin chlorophyll a/c protein	Lhcr4 PHATRDRAFT_17766	215
Phaeodactylum tricornutum	CCAP 1055/1	B7G6N5	Protein fucoxanthin chlorophyll a/c protein (Fragment)	Lhcr5 PHATRDRAFT_29472	246
Phaeodactylum tricornutum	CCAP 1055/1	B7G4U8	Protein fucoxanthin chlorophyll a/c protein	Lhcr6 PHATRDRAFT_56319	260
Phaeodactylum tricornutum	CCAP 1055/1	B7FSI1	Protein fucoxanthin chlorophyll a/c protein	Lhcr7 PHATRDRAFT_43522	270
Phaeodactylum tricornutum	CCAP 1055/1	B7FQS0	Protein fucoxanthin chlorophyll a/c protein	Lhcr8 PHATRDRAFT_32294	217
Phaeodactylum tricornutum	CCAP 1055/1	B5Y4K0	Fucoxanthin chlorophyll a/c protein, lhcr type	Lhcr9 PHATR_43860	279
Phaeodactylum tricornutum	CCAP 1055/1	B7GCV9	Protein fucoxanthin chlorophyll a/c protein	Lhcr10 PHATRDRAFT_50086	232

Phaeodactylum tricornutum	CCAP 1055/1	B7GAS4	Protein fucoxanthin chl a/c protein	Lhcr11 PHATRDRAFT_23257	246
Phaeodactylum tricornutum	CCAP 1055/1	B7FQE0	Protein fucoxanthin chlorophyll a/c protein	Lhcr12 PHATRDRAFT_54027	202
Phaeodactylum tricornutum	CCAP 1055/1	B7G502	Protein fucoxanthin chlorophyll a/c protein	Lhcr13 PHATRDRAFT_38121	206
Phaeodactylum tricornutum	CCAP 1055/1	B7G503	Protein fucoxanthin chlorophyll a/c protein	Lhcr14 PHATRDRAFT_47813	198
Phaeodactylum tricornutum	CCAP 1055/1	B7FYL0	Protein fucoxanthin chlorophyll a/c protein	Lhcx1 PHATRDRAFT_27278	209
Phaeodactylum tricornutum	CCAP 1055/1	B7FR60	Protein fucoxanthin chlorophyll a/c protein	Lhcx2 PHATRDRAFT_54065	203
Phaeodactylum tricornutum	CCAP 1055/1	B7FVF9	Protein fucoxanthin chlorophyll a/c protein	Lhcx3 PHATRDRAFT_44733	210
Phaeodactylum tricornutum	CCAP 1055/1	B7G6N3	Protein fucoxanthin chlorophyll a/c protein	Lhcx4 PHATRDRAFT_38720	212
Thalassiosira pseudonana		B8CFW3	Fucoxanthin chlorophyll a/c protein 1 (Fragment)	Lhcf1 THAPSDRAFT_38583	190
Thalassiosira pseudonana		B8CEV8	Fucoxanthin chlorophyll a/c protein 2 (Fragment)	Lhcf2 THAPSDRAFT_260392	198
Thalassiosira pseudonana		B8CFG5	Fucoxanthin chlorophyll a/c protein 4	Lhcf4 THAPSDRAFT_38667	204
Thalassiosira pseudonana		B8CEV5	Fucoxanthin chlorophyll a/c protein 5	Lhcf5 THAPSDRAFT_42962	200
Thalassiosira pseudonana		B8BX92	Fucoxanthin chlorophyll a/c protein 6	Lhcf6 THAPSDRAFT_33018	201
Thalassiosira pseudonana		B8BX93	Fucoxanthin chlorophyll a/c light-harvesting protein, major type	Lhcf7 THAPSDRAFT_21667	194
Thalassiosira pseudonana		B8C261	Fucoxanthin chlorophyll a/c protein 8	Lhcf8 THAPSDRAFT_5174	257

Thalassiosira pseudonana	B8BS67	Fucoxanthin chlorophyll a/c protein 8	Lhcf9 THAPSDRAFT_268127	210
Thalassiosira pseudonana	B5YM25	Fucoxanthin chl a/c light-harvesting protein, major type	Lhcf10 THAPS_25402	201
Thalassiosira pseudonana	B8BVI1	Fucoxanthin-chlorophyll a-c binding protein, plastid	Lhcf11 THAPSDRAFT_270241	196
Thalassiosira pseudonana	B8C8Q0	Fucoxanthin chl a/c light-harvesting protein, lhcr type	Lhcr1 THAPSDRAFT_24080	204
Thalassiosira pseudonana	B8C2K6	Fucoxanthin chl a/c light-harvesting protein	Lhcr3 THAPSDRAFT_26246	198
Thalassiosira pseudonana	B8C0K3	Fucoxanthin chl a/c light-harvesting protein	Lhcr4 THAPSDRAFT_4882	203
Thalassiosira pseudonana	B5YM80	Fucoxanthin chlorophyll a/c light-harvesting protein	Lhcr5 THAPS_25433	252
Thalassiosira pseudonana	B8BYV4	Fucoxanthin chl a/c light-harvesting protein	Lhcr7 THAPSDRAFT_3816	214
Thalassiosira pseudonana	B8LDJ7	Fucoxanthin chl a/c light-harvesting protein	Lhcr8 THAPSDRAFT_11272	222
Thalassiosira pseudonana	B8C7L8	Fucoxanthin chl a/c light-harvesting protein	Lhcr9 THAPSDRAFT_23889	256
Thalassiosira pseudonana	B8C2Y4	Fucoxanthin chlorophyll a/c light-harvesting protein	Lhcr10 THAPSDRAFT_22747	220
Thalassiosira pseudonana	B8BU33	Fucoxanthin chlorophyll a/c light-harvesting protein	Lhcr11 THAPSDRAFT_2342	252
Thalassiosira pseudonana	B8BU32	Fucoxanthin chlorophyll a/c protein	Lhcr12 THAPSDRAFT_2341	254
Thalassiosira pseudonana	B8C0K4	Fucoxanthin chlorophyll a/c light-harvesting protein, lhcr type	Lhcr14 THAPSDRAFT_4883	203
Thalassiosira pseudonana	B8CGG0	Fucoxanthin chlorophyll a/c protein, LI818 clade	lhcx1 THAPSDRAFT_264921	209

Thalassiosira pseudonana		B8CGG2	Fucoxanthin chlorophyll a/c protein, LI818 clade	Lhcx2 THAPSDRAFT_38879	209
Thalassiosira pseudonana		B8C364	Fucoxanthin chlorophyll a/c protein, LI818 clade	Lhcx4 THAPSDRAFT_270228	231
Thalassiosira pseudonana		B8BSG2	Fucoxanthin chlorophyll a/c protein, LI818 clade	lhcx5 THAPSDRAFT_31128	236
Thalassiosira pseudonana		B8CGG1	Fucoxanthin chlorophyll a/c protein, LI818 clade	Lhcx6 THAPSDRAFT_12097	255
Thalassiosira pseudonana		B5YLU3	Fucoxanthin chlorophyll a/c protein-LI818 clade	Lhcx6_1 THAPS_30385	199
Fragilariopsis cylindrus	CCMP 1102	A0A1E7FWN6	Chlorophyll a/b-binding protein	LHCF1 FRACYDRAFT_267324	253
Fragilariopsis cylindrus	CCMP 1102	A0A1E7EP91	Chlorophyll a/b-binding protein	LHCF2 FRACYDRAFT_197251	275
Fragilariopsis cylindrus	CCMP 1102	A0A1E7FC96	Chlorophyll a/b-binding protein	LHCF3 FRACYDRAFT_170453	250
Fragilariopsis cylindrus	CCMP 1102	A0A1E7EX55	Chlorophyll a/b-binding protein	LHCF4 FRACYDRAFT_271208	216
Fragilariopsis cylindrus	CCMP 1102	A0A1E7F9G5	Chloroa_b-bind-domain-containing protein	LHCF5 FRACYDRAFT_170761	204
Fragilariopsis cylindrus	CCMP 1102	A0A1E7FWS4	Fucoxanthin chlorophyll a/c binding protein	LHCF6 FRACYDRAFT_267329	206
Fragilariopsis cylindrus	CCMP 1102	A0A1E7EV37	Fucoxanthin chlorophyll a/c-binding protein	LHCF7 FRACYDRAFT_271332	207
Fragilariopsis cylindrus	CCMP 1102	A0A1E7ETP3	Chlorophyll a/b-binding protein	LHCF8 FRACYDRAFT_271435	235

Fragilariopsis cylindrus	CCMP 1102	A0A1E7ETG6	Fucoxanthin chlorophyll a/c protein	LHCF9 FRACYDRAFT_271557	208
Fragilariopsis cylindrus	CCMP 1102	A0A1E7ETL9	Fucoxanthin chlorophyll a/c protein, lhcf type	LHCF10 FRACYDRAFT_271561	197
Fragilariopsis cylindrus	CCMP 1102	A0A1E7FR61	Chlorophyll a/b-binding protein	LHCF11 FRACYDRAFT_205888	203
Fragilariopsis cylindrus	CCMP 1102	A0A1E7EPC2	Fucoxanthin chlorophyl a/c protein	LHCF12 FRACYDRAFT_271931	203
Fragilariopsis cylindrus	CCMP 1102	A0A1E7FMF5	Fucoxanthin chlorophyll protein	LHCF13 FRACYDRAFT_207327	208
Fragilariopsis cylindrus	CCMP 1102	A0A1E7FT71	Fucoxanthin chlorophyl a/c protein	LHCF14 FRACYDRAFT_267837	206
Fragilariopsis cylindrus	CCMP 1102	A0A1E7ENI1	Fucoxanthin chlorophyll a/c protein	LHCF15 FRACYDRAFT_174589	222
Fragilariopsis cylindrus	CCMP 1102	A0A1E7F634	Fucoxanthin-chlorophyll a-c binding protein	LHCF16 FRACYDRAFT_269868	199
Fragilariopsis cylindrus	CCMP 1102	A0A1E7FR49	Fucoxanthin-chlorophyll a-c binding protein	LHCF17 FRACYDRAFT_267702	203
Fragilariopsis cylindrus	CCMP 1102	A0A1E7FHF3	Fucoxanthin-chlorophyll a-c binding protein	LHCF18 FRACYDRAFT_268624	203
Fragilariopsis cylindrus	CCMP 1102	A0A1E7FGF8	Fucoxanthin chlorophyll a/c protein	LHCF19 FRACYDRAFT_268626	199

Fragilariopsis cylindrus	CCMP 1102	A0A1E7F865	Fucoxanthin chlorophyll a/c protein	LHCF20 FRACYDRAFT_269543	210
Fragilariopsis cylindrus	CCMP 1102	A0A1E7F823	Fucoxanthin-chlorophyll a-c binding protein	LHCF21 FRACYDRAFT_269567	203
Fragilariopsis cylindrus	CCMP 1102	A0A1E7FZY1	Fucoxanthin chlorophyll a/c protein	LHCF22 FRACYDRAFT_267576	210
Fragilariopsis cylindrus	CCMP 1102	A0A1E7EQD0	Fucoxanthin chlorophyll a/c protein (Fragment)	LHCF23 FRACYDRAFT_143190	189
Fragilariopsis cylindrus	CCMP 1102	A0A1E7ELA3	Fucoxanthin chlorophyll a/c protein	LHCR1 FRACYDRAFT_278371	306
Fragilariopsis cylindrus	CCMP 1102	A0A1E7F7J1	Chlorophyll a/b-binding protein	LHCR2 FRACYDRAFT_210115	223
Fragilariopsis cylindrus	CCMP 1102	A0A1E7FCV6	Fucoxanthin chlorophyll a/c protein	LHCR3 FRACYDRAFT_275731	336
Fragilariopsis cylindrus	CCMP 1102	A0A1E7EUA9	Fucoxanthin chl a/c protein	LHCR4 FRACYDRAFT_271559	255
Fragilariopsis cylindrus	CCMP 1102	A0A1E7F2J7	Fucoxanthin chlorophyll a/c protein	LHCR5 FRACYDRAFT_270606	248
Fragilariopsis cylindrus	CCMP 1102	A0A1E7F6J6	Chlorophyll a/b-binding protein	LHCR6 FRACYDRAFT_209926	200
Fragilariopsis cylindrus	CCMP 1102	A0A1E7FU55	Fucoxanthin chlorophyll a/c protein	LHCR7 FRACYDRAFT_177731	323

Fragilariopsis cylindrus	CCMP 1102	A0A1E7FLB3	Fucoxanthin chlorophyll a/c protein	LHCR8 FRACYDRAFT_274939	199
Fragilariopsis cylindrus	CCMP 1102	A0A1E7EZF1	Fucoxanthin chlorophyll a/c protein	LHCR9 FRACYDRAFT_270940	310
Fragilariopsis cylindrus	CCMP 1102	A0A1E7FEE6	Chlorophyll a/b-binding protein	LHCR10 FRACYDRAFT_261294	206
Fragilariopsis cylindrus	CCMP 1102	A0A1E7F6C5	Fucoxanthin chlorophyll a/c protein	LHCR11 FRACYDRAFT_269925	213
Fragilariopsis cylindrus	CCMP 1102	A0A1E7F6E2	Fucoxanthin chlorophyll a/c protein	LHCR12 FRACYDRAFT_269918	200
Fragilariopsis cylindrus	CCMP 1102	A0A1E7FEF2	Fucoxanthin chlorophyll a/c protein	LHCR13 FRACYDRAFT_208340	223
Fragilariopsis cylindrus	CCMP 1102	A0A1E7FJ44	Fucoxanthin chlorophyll a/c protein	LHCR14 FRACYDRAFT_260998	414
Fragilariopsis cylindrus	CCMP 1102	A0A1E7FCY5	Light-harvesting protein	LHCR15 FRACYDRAFT_240107	275
Fragilariopsis cylindrus	CCMP 1102	A0A1E7EMR9	Chloroa_b-bind-domain-containing protein	Lhcx1 FRACYDRAFT_272042	224
Fragilariopsis cylindrus	CCMP 1102	A0A1E7F884	Chloroa_b-bind-domain-containing protein	Lhcx4 FRACYDRAFT_271659	243
Fragilariopsis cylindrus	CCMP 1102	A0A1E7F771	Chlorophyll a/b-binding protein	Lhcx3 FRACYDRAFT_209887	209

Fragilariopsis cylindrus	CCMP 1102	A0A1E7ERH1	Fucoxanthin chlorophyll a/c binding protein	Lhcx5 FRACYDRAFT_271962	226
Fragilariopsis cylindrus	CCMP 1102	A0A1E7ENQ4	Chloroa_b-bind-domain-containing protein	Lhcx6 FRACYDRAFT_188478	229
Fragilariopsis cylindrus	CCMP 1102	A0A1E7F6H3	Fucoxanthin chlorophyll a/c binding protein	Lhcx7 FRACYDRAFT_267951	227
Fragilariopsis cylindrus	CCMP 1102	A0A1E7FTV2	Chloroa_b-bind-domain-containing protein	Lhcx8 FRACYDRAFT_244998	267
Fragilariopsis cylindrus	CCMP 1102	A0A1E7F110	Fucoxanthin chlorophyll a/c binding protein	Lhcx9 FRACYDRAFT_192081	245
Fragilariopsis cylindrus	CCMP 1102	A0A1E7F1E5	Fucoxanthin chlorophyll a/c binding protein	Lhcx10 FRACYDRAFT_271005	246
Fragilariopsis cylindrus	CCMP 1102	A0A1E7EYG1	Chloroa_b-bind-domain-containing protein	Lhcx11 FRACYDRAFT_269313	240
Fragilariopsis cylindrus	CCMP 1102	A0A1E7FAZ1	Fucoxanthin chlorophyll a/c binding protein	Lhcx2 FRACYDRAFT_218498	220

Table S3 BLAST comparison between FCP proteins in *P. tricornutum* and the whole proteome of *T. lutea*. Green: Lhcf sequences. Blue: Lhcr sequences. Orange: Lhcx sequences. Yellow: maximum average of bitscores from Lhcf, Lhcr or Lhcx family for each *T. lutea* sequence. Underlined scores in green, blue and orange are the highest scores of each comparison line.

(Excel file submitted separately)

Table S4 BLAST comparison between FCP proteins in *F. cylindrus* and the whole proteome of *T. lutea*. Green: Lhcf sequences. Blue: Lhcr sequences. Orange: Lhcx sequences. Yellow: maximum average of bitscores from Lhcf, Lhcr or Lhcx family for each *T. lutea* sequence. Underlined scores in green, blue and orange are the highest scores of each comparison line.

(Excel file submitted separately)

Table S5 BLAST comparison between FCP proteins in *T. pseudonana* and the whole proteome of *T. lutea*. Green: Lhcf sequences. Blue: Lhcr sequences. Orange: Lhcx sequences. Yellow: maximum average of bitscores from Lhcf, Lhcr or Lhcx family for each *T. lutea* sequence. Underlined scores in green, blue and orange are the highest scores of each comparison line.

(Excel file submitted separately)

Table S6 FCP protein sequences of *E. huxleyi*, *S. japonica*, *S. latissima, C. gracilis* used for building the cladogram of the Lhcf family

FCP sequences of other algae species used for PhyML (UniprotKB)							
**Organism**	**Strain**	**Entry**	**Protein family**	**Protein names**	**Length**	**Annotation in McKew *et al.* 2013**	**New annotation proposed**
Emiliania huxleyi		R1ESZ0	Light harvesting protein	Lhcf1 EMIHUDRAFT_457448	231	Lhcf(red)	Lhcr1
Emiliania huxleyi		R1ED82	Light harvesting protein	Lhcf2 EHUX00137_LOCUS22769 EHUX00138_7441 EMIHUDRAFT_413829	198	Lhcf(red)	Lhcr2
Emiliania huxleyi		R1D2Y4	Light harvesting protein	Lhcf3 EMIHUDRAFT_439022	208	Lhcf(red)	Lhcr3
Emiliania huxleyi		R1DWS2	Light harvesting protein	Lhcf4_2 Lhcf4_1 EMIHUDRAFT_438393 EMIHUDRAFT_445064	239	Lhcf(red)	Lhcr4
Emiliania huxleyi		R1FED1	Light harvesting protein	Lhcf5 EMIHUDRAFT_441453	201	Lhcf(red)	Lhcr5
Emiliania huxleyi		R1BYL8	Light harvesting protein	Lhcf6_1 EMIHUDRAFT_211477 EMIHUDRAFT_447013	213	Lhcf group II	Lhcf1
Emiliania huxleyi		R1DY94	Light harvesting protein	Lhcf7 EMIHUDRAFT_419663	198	Lhcf group I	Lhcf2 group A
Emiliania huxleyi		R1DSS8	Light harvesting protein (Fragment)	Lhcf8 EMIHUDRAFT_46861	178	Lhcf group I	Lhcf3
Emiliania huxleyi		R1DXP9	Light harvesting protein	Lhcf9_1 EMIHUDRAFT_260243	197	Lhcf group I	Lhcf4 group A
Emiliania huxleyi		R1FED0	Chloroplast light harvesting protein	Lhcf9_2 EMIHUDRAFT_231654	120	Lhcf group I	Lhcf5 group A

Emiliania huxleyi	R1DCN2	Light harvesting protein	Lhcf10_2 Lhcf10_1 EMIHUDRAFT_433058 EMIHUDRAFT_434108	210	Lhcf group I	Lhcf6 group A
Emiliania huxleyi	R1CYJ0	Light harvesting protein	Lhcf12_1 EMIHUDRAFT_249526	260	Lhcf group I	Lhcf7 group A
Emiliania huxleyi	R1DRG2	Light harvesting protein	Lhcf12_2 EMIHUDRAFT_200525	260	Lhcf group I	Lhcf8 group A
Emiliania huxleyi	R1C0A3	Light harvesting protein	Lhcf13 EMIHUDRAFT_66220	249	Lhcf group I	Lhcf9 group A
Emiliania huxleyi	R1EIA6	Light harvesting protein	Lhcf14 EHUX00154_24932 EMIHUDRAFT_432006	255	Lhcf group I	Lhcf10 group A
Emiliania huxleyi	R1CMT6	Light harvesting protein	Lhcf15_1 EMIHUDRAFT_415729	253	Lhcf group I	Lhcf11 group A
Emiliania huxleyi	R1B352	Light harvesting protein	Lhcf15_2 EMIHUDRAFT_446702	220	Lhcf group I	Lhcf12 group A
Emiliania huxleyi	R1EB65	Light harvesting protein	Lhcf16_1 EMIHUDRAFT_435472	257	Lhcf group I	Lhcf13 group A
Emiliania huxleyi	R1B4H5	Light harvesting protein	Lhcf16_2 EMIHUDRAFT_372663	262	Lhcf group I	Lhcf14 group A
Emiliania huxleyi	R1FP66	Light harvesting protein	Lhcf17 EMIHUDRAFT_363095	244	Lhcf group I	Lhcf15 group A
Emiliania huxleyi	R1CV07	Light harvesting protein	Lhcf18 Lhcf56 EMIHUDRAFT_353537 EMIHUDRAFT_443305	254	Lhcf group I	Lhcf16 group A
Emiliania huxleyi	R1DNR0	Light harvesting protein	Lhcf19_1 EMIHUDRAFT_422150	245	Lhcf group I	Lhcf17 group A
Emiliania huxleyi	R1FYQ0	Light harvesting protein	Lhcf19_2 EMIHUDRAFT_77826	213	Lhcf group I	Lhcf18 group A
Emiliania huxleyi	R1DTF1	Light harvesting protein	Lhcf20_1 EMIHUDRAFT_356694	281	Lhcf group I	Lhcf19 group A

Emiliania huxleyi	R1DKB4	Light harvesting protein (Fragment)	Lhcf20_2 EMIHUDRAFT_362770	281	Lhcf group I	Lhcf20 group A
Emiliania huxleyi	R1CGG7	Light harvesting protein	Lhcf21_1 Lhcf21_2 EMIHUDRAFT_67603 EMIHUDRAFT_74777	260	Lhcf group I	Lhcf21 group A
Emiliania huxleyi	R1C922	Light harvesting protein	Lhcf22 EMIHUDRAFT_65742	261	Lhcf group I	Lhcf22 group A
Emiliania huxleyi	R1EY91	Light harvesting protein	Lhcf23 EMIHUDRAFT_64682	265	Lhcf group I	Lhcf23 group A
Emiliania huxleyi	R1DAV6	Light harvesting protein	Lhcf24 EMIHUDRAFT_438462	268	Lhcf group I	Lhcf24 group A
Emiliania huxleyi	R1DCB2	Light harvesting protein	Lhcf25_1 EMIHUDRAFT_441864	201	Lhcf group II	Lhcf25
Emiliania huxleyi	R1F3M2	Light harvesting protein	Lhcf25_2 EMIHUDRAFT_442651	201	Lhcf group II	Lhcf26
Emiliania huxleyi	R1B3D6	Light harvesting protein (Fragment)	Lhcf26_1 EHUX00137_LOCUS42418 EHUX00138_35162 EMIHUDRAFT_360421	204	Lhcf group II	Lhcf27 group B
Emiliania huxleyi	R1DT91	Lught harvesting protein	Lhcf26_2 EMIHUDRAFT_431505	173	Lhcf group II	Lhcf28
Emiliania huxleyi	R1EGM0	Light harvesting protein	Lhcf27 EMIHUDRAFT_435222	242	Lhcf group II	Lhcf29 group B
Emiliania huxleyi	R1FJF4	Light harvesting protein	Lhcf28 EMIHUDRAFT_70030	181	Lhcf group II	Lhcf30 group B
Emiliania huxleyi	R1D667	Light harvesting protein	Lhcf29 EHUX00154_13092 EMIHUDRAFT_417035	198	Lhcf group II	Lhcf31 group B
Emiliania huxleyi	R1BVJ8	Light harvesting protein	Lhcf30_1 EMIHUDRAFT_437015	228	Lhcf group II	Lhcf32 group B

Emiliania huxleyi	R1DNW2	Light harvesting protein (Fragment)	Lhcf30_2 EMIHUDRAFT_420179	132	Lhcf group II	Lhcf33 group B
Emiliania huxleyi	R1F723	Light harvesting protein	Lhcf31 Lhcf64 EMIHUDRAFT_434417 EMIHUDRAFT_446310	212	Lhcf group II	Lhcf34 group B
Emiliania huxleyi	R1CP00	Light harvesting protein	Lhcf32 EMIHUDRAFT_443878	221	Lhcf group II	Lhcf35 group B
Emiliania huxleyi	R1BH65	Light harvesting protein	Lhcf33 EHUX00137_LOCUS43769 EMIHUDRAFT_358662	206	Lhcf group II	Lhcf36 group B
Emiliania huxleyi	R1DK54	Light harvesting protein	Lhcf34_1 EMIHUDRAFT_467343	234	LI818-like	Lhcx1
Emiliania huxleyi	R1DA28	Light harvesting protein (Fragment)	Lhcf34_2 EMIHUDRAFT_45662	171	LI818-like	Lhcx2
Emiliania huxleyi	R1CTY2	Light harvesting protein	Lhcf35 EMIHUDRAFT_422697	229	LI818-like	Lhcx3
Emiliania huxleyi	R1CQ26	Light-harvesting family	Lhcf36 EMIHUDRAFT_443721	232	LI818-like	Lhcx4
Emiliania huxleyi	R1FXU0	Light harvesting protein	Lhcf36_2 EMIHUDRAFT_69651	182	LI818-like	Lhcx5
Emiliania huxleyi	R1BGZ8	Light harvesting protein	Lhcf37 EHUX00137_LOCUS16712 EHUX00138_9872 EHUX00154_24926 EMIHUDRAFT_217340	191	LI818-like	Lhcx6
Emiliania huxleyi	R1EFV4	Light harvesting protein	Lhcf38 EMIHUDRAFT_430242	191	LI818-like	Lhcx7
Emiliania huxleyi	R1CYU0	Light harvesting protein (Fragment)	Lhcf39_1 Lhcf39_2 EMIHUDRAFT_45591 EMIHUDRAFT_45605	173	LI818-like	Lhcx8

Emiliania huxleyi	R1EIV2	Light harvesting protein (Light-harvesting family)	Lhcf40 Lhcf51 Lhcf52 EMIHUDRAFT_417267 EMIHUDRAFT_419203 EMIHUDRAFT_419207 EMIHUDRAFT_428685	235	LI818-like	Lhcx9
Emiliania huxleyi	R1BA06	Light harvesting protein	Lhcf42 EMIHUDRAFT_460117	221	LI818-like	Lhcx10
Emiliania huxleyi	R1DG90	Light harvesting protein	Lhcf43 EHUX00137_LOCUS39159 EMIHUDRAFT_416733	221	LI818-like	Lhcx11
Emiliania huxleyi	R1FE18	Light harvesting protein	Lhcf44 EMIHUDRAFT_442232	222	Lhcz-like	Lhcr6 (Lhcz)
Emiliania huxleyi	R1DSW2	Light harvesting protein	Lhcf45_2 Lhcf45_1 EHUX00137_LOCUS46436 EHUX00154_72799 EMIHUDRAFT_203047 EMIHUDRAFT_224646	249	Lhcz-like	Lhcr7 (Lhcz)
Emiliania huxleyi	R1DB89	Light harvesting protein	Lhcf46_1 Lhcf46_2 EHUX00154_2922 EMIHUDRAFT_419743 EMIHUDRAFT_434226	299	Lhcz-like	Lhcr8 (Lhcz)
Emiliania huxleyi	R1DLJ5	Light harvesting protein	Lhcf47 EMIHUDRAFT_451739	233	Lhcz-like	Lhcr9 (Lhcz)
Emiliania huxleyi	R1DG54	Light harvesting protein	Lhcf48 EMIHUDRAFT_356951	241	Lhcz-like	Lhcr10 (Lhcz)
Emiliania huxleyi	R1F997	Light harvesting protein	Lhcf53 Lhcf49 EMIHUDRAFT_358243 EMIHUDRAFT_364696	305	Lhcz-like	Lhcr11 (Lhcz)
Emiliania huxleyi	R1DWM4	Light harvesting protein	Lhcf54 EMIHUDRAFT_76288	140	Lhcz-like	Lhcr12 (Lhcz)

Emiliania huxleyi	R1E324	Light harvesting protein	Lhcf55 EMIHUDRAFT_461003	160	Lhcf(red)	Lhcr13
Emiliania huxleyi	R1CGF0	Light harvesting protein	Lhcf57 EMIHUDRAFT_240836	411	Lhcf group I	Lhcf37 group A
Emiliania huxleyi	R1DMQ2	Light harvesting protein	Lhcf58 EMIHUDRAFT_362550	211	Lhcf group I	Lhcf38
Emiliania huxleyi	R1BTM8	Light harvesting protein	Lhcf59 Lhcf11 EHUX00154_11279 EMIHUDRAFT_432202 EMIHUDRAFT_459601	224	Lhcf group I	Lhcf39 group A
Emiliania huxleyi	R1DXB5	Light harvesting protein (Fragment)	Lhcf60 EMIHUDRAFT_75517	113	Lhcf group I	Lhcf40 group A
Emiliania huxleyi	R1FI70	Light harvesting protein (Fragment)	Lhcf61 EMIHUDRAFT_46509	165	Lhcf group II	Lhcf41 group B
Emiliania huxleyi	R1F987	Light harvesting protein	Lhcf62 EHUX00138_16999 EMIHUDRAFT_433847	277	Lhcf group II	Lhcf42 group B
Emiliania huxleyi	R1DZZ7	Light harvesting protein	Lhcf63 EMIHUDRAFT_312801	290	Lhcf group II	Lhcf43 group B
Emiliania huxleyi	R1F0Q3	Light harvesting protein (Fragment)	Lhcf65 EMIHUDRAFT_366130	190	Lhcf group II	Lhcf44 group B
Emiliania huxleyi	R1C0U8	Light harvesting protein	Lhcf67 Lhcf66 EMIHUDRAFT_353073 EMIHUDRAFT_355940 EMIHUDRAFT_370423	190	Lhcf group II	Lhcf45 group B
Emiliania huxleyi	R1D681	Light harvesting protein	Lhcf68 EMIHUDRAFT_312310	183	Lhcf group II	Lhcf46 group B
Emiliania huxleyi	R1DDZ6	Light harvesting protein	Lhcf69 EMIHUDRAFT_455747	213	Lhcf(red)	Lhcr14
Emiliania huxleyi	R1E922	Light harvesting protein	Lhcf71 EMIHUDRAFT_632166	229	Lhcf(red)	Lhcr15

Emiliania huxleyi	R1EGS8	Light harvesting protein	Lhcf73 EMIHUDRAFT_237298	165	LI818-like	Lhcx12
Emiliania huxleyi	R1CL78	Light harvesting protein	Lhcf74 EMIHUDRAFT_254989	219	Lhcf group I	Lhcf47 group A
Emiliania huxleyi	R1EFU9	Light harvesting protein	Lhcf75 Lhcf41 EHUX00138_9869 EHUX00138_9870 EMIHUDRAFT_436450 EMIHUDRAFT_444842	221	LI818-like	Lhcx13
Saccharina japonica	Q2XU52	Chloroplast light harvesting protein lhcf6	lhcf6	218		
Saccharina latissima	Q94612	Fucoxanthin chlorophyll a/c binding protein (Fragment)	Lhcf1	198		
Saccharina latissima	Q9FEP6	Light harvesting protein lhcf2	lhcf2	218		
Saccharina latissima	Q9FEP5	Light harvesting protein lhcf3 (Fragment)	lhcf3	197		
Saccharina latissima	Q9FEP4	Light harvesting protein lhcf4	lhcf4	218		
Saccharina latissima	Q9FEP3	Light harvesting protein lhcf5	lhcf5	218		
Saccharina latissima	Q9FEP2	Light harvesting protein lhcf6	lhcf6	218		
Saccharina latissima	Q9FEP1	Light harvesting protein lhcf7	lhcf7	218		
Chaetoceros gracilis	A0A6J4 B1C2	Fucoxanthin chlorophyll a/c protein 12	Lhcq2	204		
Chaetoceros gracilis	A0A6J4 B125	Fucoxanthin chlorophyll a/c protein 16	Lhcq6	206		

Table S7 Identification of the Lhcf, Lhcr, Lhcx and putative Lhcz protein sequences in *T. lutea*

**FCP proteins in *T. lutea***							
TISO_00499	**Lhcx1**	TISO_02238	**Lhcr1**		TISO_07452	**Lhcf1**	Group B
TISO_06854	**Lhcx2**	TISO_02239	**Lhcr2**		TISO_07587	**Lhcf2**	Group B
TISO_07276	**Lhcx3**	TISO_07183	**Lhcr3**		TISO_08095	**Lhcf3**	Group B
TISO_11242	**Lhcx4**	TISO_07308	**Lhcr4**		TISO_08517	**Lhcf4**	Group A
TISO_22079	**Lhcx5?**	TISO_09963	**Lhcr5**	**Lhcz?**	TISO_11770	**Lhcf5**	Group A
TISO_23378	**Lhcx6**	TISO_23096	**Lhcr6**	**Lhcz?**	TISO_12017	**Lhcf6**	Group A
TISO_23447	**Lhcx7**	TISO_23827	**Lhcr7**		TISO_12558	**Lhcf7**	Group A
TISO_23500	**Lhcx8**	TISO_24841	**Lhcr8**		TISO_14333	**Lhcf8**	Group B
TISO_23568	**Lhcx9**	TISO_28748	**Lhcr9**		TISO_14677	**Lhcf9**	Group B
TISO_23574	**Lhcx10**	TISO_29671	**Lhcr10**	**Lhcz?**	TISO_16944	**Lhcf10**	Group B
TISO_24321	**Lhcx11**	TISO_32585	**Lhcr11**		TISO_16947	**Lhcf11**	Group B
TISO_26583	**Lhcx12**	TISO_33089	**Lhcr12**		TISO_17799	**Lhcf12**	Group A
					TISO_18058	**Lhcf13**	Group B
					TISO_18150	**Lhcf14**	Group B
					TISO_18151	**Lhcf15**	Group B
					TISO_19125	**Lhcf16**	Group A
					TISO_19286	**Lhcf17**	Group A
					TISO_20897	**Lhcf18**	Group B
					TISO_22153	**Lhcf19**	Group B
					TISO_22154	**Lhcf20**	Group B
					TISO_22383	**Lhcf21**	Group A
					TISO_24608	**Lhcf22**	Group A
					TISO_25321	**Lhcf23**	Group A
					TISO_26704	**Lhcf24**	Group A
					TISO_28817	**Lhcf25**	Group A
					TISO_36599	**Lhcf26**	Group B
					TISO_36600	**Lhcf27**	Group B
					TISO_36835	**Lhcf28**	Group A

Fig. S1 Cladogram of the FCP protein sequences of *E. huxkeyi*. Green: Lhcf groups A and B. Blue: Lhcr. Orange: Lhcx.

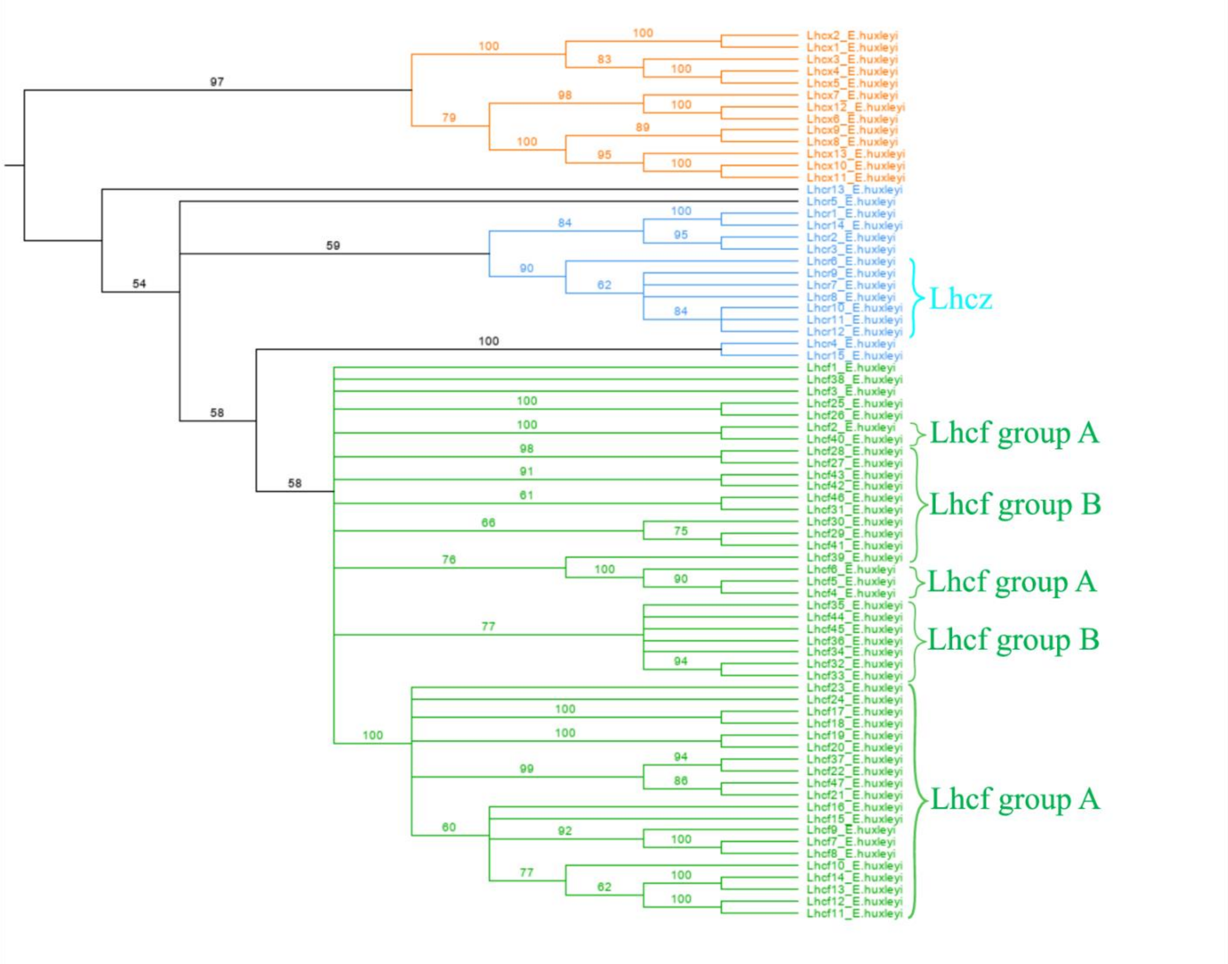

Fig. S2 Cladogram of the Lhcr sequences of *E. huxkeyi* and *T. lutea*. Dark blue: Lhcr. Light blue: putative Lhcz.

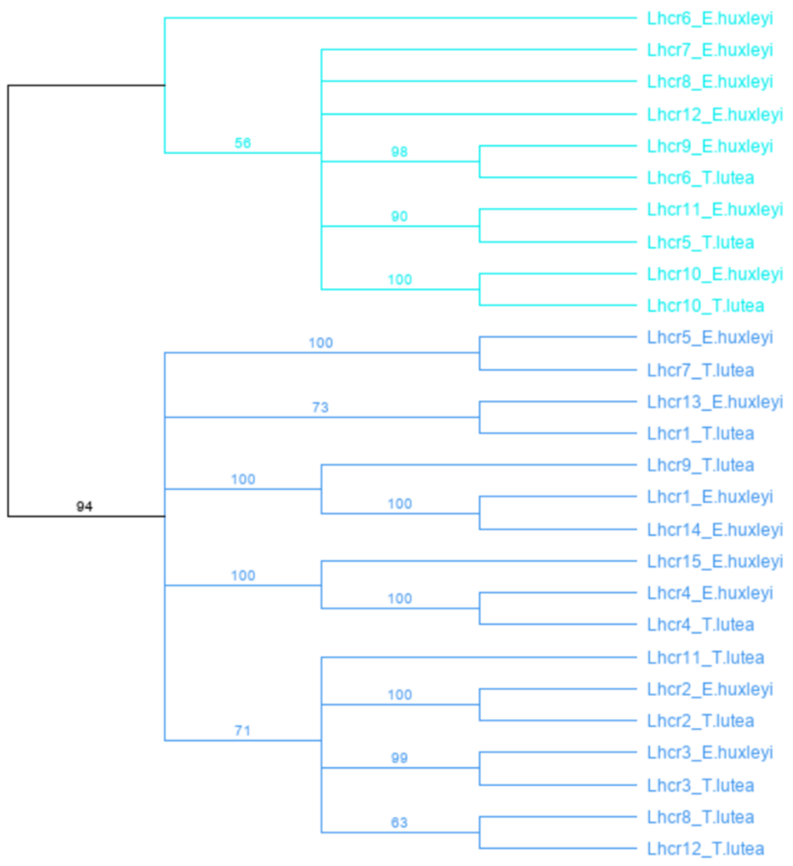

Fig. S3 Binding sites of Chl a, Chl c and Fx in *T. lutea* compared with *P. tricornutum* (Wang et al., 2019)

Q : Chl a/c central ligand, residues conserved in most FCP subunits
T : Chl a/c central ligand, non-conserved ligand residues in FCP subunits
F : Chl a/c H bond
L : Fx binding site (can be multiple)
Y : Putative Fx binding sites

a401 | Fx301 | Fx303 + a402 | c403 + Fx306 via c403

```
Lhcf1_P.tricornutum     ---------------GAQQPLGYWDPLGLVA-----DGD------QEKFDRLR------- 64
Lhcf2_P.tricornutum     ---------------GAQAPLGFYDPLGLVA-----DGD------QEKFDRLR------- 64
Lhcf3_P.tricornutum     ---------------GAQPPLGFFDPLGLVA-----DGD------QEKFDRLR------- 64
Lhcf4_P.tricornutum     ---------------GAQPPLGFFDPLGLVA-----DGD------QEKFDRLR------- 64
Lhcf5_P.tricornutum     ---------------GAQPPLGFFDPLGLVA-----DGD------QEKFDRLR------- 64
Lhcf6_P.tricornutum     ---------------GVTAPLGFYDPLGLVS-----DGD------EAKFKRLR------- 66
Lhcf7_P.tricornutum     ---------------GVTAPLGFYDPLGLVS-----DGD------EAKFKRLR------- 66
Lhcf8_P.tricornutum     ---------------GAQPPLGFFDPLGLVK-----DGD------QATFDRLR------- 65
Lhcf9_P.tricornutum     ---------------GATLPLGFWDPLGLVA-----DGN------QEKFDRLR------- 64
Lhcf10_P.tricornutum    ---------------GAQPPLGFYDPLGLLD-----GAS------ESKFERLR------- 64
Lhcf11_P.tricornutum    ---------------GAQPPLGFFDPLGLVA-----DGD------QEKFDRLR------- 64
Lhcf12_P.tricornutum    ---------------GVQKPLGFWDPLSVCD-----GAD------QARFDELR------- 50
Lhcf13_P.tricornutum    ---------------GVIEPTGFFDPLGLSK-----DID------QEKFDQYR------- 64
Lhcf14_P.tricornutum    ---------------GVIQPTGFFDPFGLSK-----GKD------MATFDQYR------- 63
Lhcf15_P.tricornutum    ---------------GAIDPVGFFDPAGLTS-----NID------EETFRRYR------- 67
Lhcf16_P.tricornutum    ---------------S-TAPLKRFDPLGLAQ-----VGS------EQTFAWFQ------- 65
Lhcf17_P.tricornutum    ---------------GVLPPIGFFDPLGFTT-----RGPYGSR--EENFRHYR------- 71

Lhcf4_T.lutea           ---------------KLNPVVGYFDPLKLGEQTFWDNTN------EETIGFLR------- 107
Lhcf5_T.lutea           ---------------P-ETGNKVFDPLGLAK-----IAS------PETLAWYR------- 64
Lhcf6_T.lutea           ---------------P-ETAGKVFDPLGLAS-----LGG------EETLAFFR------- 82
Lhcf7_T.lutea           ------------HISNLRPYFTVTDSSPLAEDEYLVEELLDRKGDGVSFKYLV------K 380
Lhcf16_T.lutea          ---------------SLNPNIGFWDPLGLAKRDFWDQGN------EATIGFLR------- 72
Lhcf17_T.lutea          ---------------KLNPAIGYYDPLGLATADFWSQGN------DATWGFLR------- 70
Lhcf21_T.lutea          ---------------GTTRPLGVWDPLGLMT-----KMP-------EKYRRFQ------- 81
Lhcf23_T.lutea          DCVHPPSSTQPPSLTGVLEPTGYFDPLNLAA-----DID------QEKFDQYR------- 87
Lhcf24_T.lutea          ---------------E-ETGNKIWDPLGISD-----AVS------DEAVMWFR------- 68
Lhcf28_T.lutea          ---------------V-EVSNKVWDPLKLSA-----KMD------EGNLNLVR------- 65
Lhcf1_T.lutea           ---------------GVTPPLGVYDPLNLLA-----EPVTYPR---RDYRRYV------- 63
Lhcf2_T.lutea           ---------------GVLPPIGRWDPLQIRE-----QGP-------ERYRRFV------- 113
Lhcf3_T.lutea           ---------------GVTPPLGLFDPLNLLS-----RGP-------EAYRRYQ------- 101
Lhcf8_T.lutea           ---------------GATGPLGYWDPLGFST-----KQP-------ERFARYR------- 63
Lhcf9_T.lutea           ---------------GVLPPLGVYDPLGLIE-----T---------RDMRRYE------- 63
Lhcf10_T.lutea          ---------------GVLPPLGVYDPLGLIE-----T---------RDMRRYE------- 341
Lhcf11_T.lutea          ---------------GVLPPLGVYDPLGLIE-----T---------RDMRRYEVRPRQH- 69
Lhcf12_T.lutea          ---------------GVIEPTGFFDPLGLSA-----KID------EETFAQYR------- 67
Lhcf13_T.lutea          ------------------------------------------------------------ 0
Lhcf14_T.lutea          ---------------GVLPPLGVYDPLGLIE-----T---------RNMYRYE------- 108
Lhcf15_T.lutea          ---------------GVLPPLGVYDPLGLIE-----T---------RNMYRYE------- 63
Lhcf18_T.lutea          ---------------GVLPPLGVWDPLGLIE-----K---------RDMRRYE------- 62
Lhcf19_T.lutea          ---------------GACKPLGFFDPLGFSK-----DAS------PASMAKFR------- 64
Lhcf20_T.lutea          ---------------GTTKPLGVYDPLGLMT-----TMP-------DKYRRFQ------- 46
Lhcf22_T.lutea          ---------------KANPVIGYWDPTPLASMEFWGQSQ------EATIGFLR------- 72
Lhcf25_T.lutea          ---------------GGTPEGKVFDPLGLGA-----DEA--------SL----------- 63
Lhcf26_T.lutea          ---------------GVLPPLGVYDPLGLIE-----T---------RDMRRYE------- 63
Lhcf27_T.lutea          ---------------GVLPPLGVYDPLGLIE-----T---------RDMRRYEVRAHRLL 50
```

a402 a406 c403 Fx302 a404 Fx302

```
Lhcf1_P.tricornutum     ----------------------------YVEIKHGRICMLAVAGYLTQE-AGIRLPGDID 95
Lhcf2_P.tricornutum     ----------------------------YVEIKHGRISMLAVAGYLAQE-AGWRLGGDIA 95
Lhcf3_P.tricornutum     ----------------------------YVEIKHGRISMLAVVGYLVQE-AGVRLPGTID 95
Lhcf4_P.tricornutum     ----------------------------YVEIKHGRISMLAVVGYLVQE-AGVRLPGTID 95
Lhcf5_P.tricornutum     ----------------------------YVEIKHGRISMLAVAGYLVQE-NGIRLPGDID 95
Lhcf6_P.tricornutum     ----------------------------FVELKHGRISMLAVVGYIATA-TGNRLPGTID 97
Lhcf7_P.tricornutum     ----------------------------FVELKHGRISMLAVVGYIATA-TGNRLPGTID 97
Lhcf8_P.tricornutum     ----------------------------YVEIKHGRIAMLAVAGYLTTE-AGYRLPGNID 96
Lhcf9_P.tricornutum     ----------------------------LVELKHGRISMLAVVGYLIQE-AGVRLPGNID 95
Lhcf10_P.tricornutum    ----------------------------YVELKHGRISMLAVVGYLVTE-AGIRLPGNID 95
Lhcf11_P.tricornutum    ----------------------------YVELKHGRISMLAVVGYLVQE-NGIRLPGDID 95
Lhcf12_P.tricornutum    ----------------------------YVEIKHGRVAMLAIVGHMVTS-AGVRWPGSI- 80
Lhcf13_P.tricornutum    ----------------------------TAELKHGRVAQLAVIGYV-VQ-EIYRFPFDIA 94
Lhcf14_P.tricornutum    ----------------------------TAELKHGRVAMLCVIGYV-VP-EFYRFPGDIA 93
Lhcf15_P.tricornutum    ----------------------------AGEIKNGRVAMLAVIGYI-VP-EFYRFPGELM 97
Lhcf16_P.tricornutum    ----------------------------AAELKHSRAAMLATTGFIVQA-AGIHFPGML- 95
Lhcf17_P.tricornutum    ----------------------------GVEVKHGRIAMAATLGTL-VQ-QNYRFEGFLS 101

Lhcf4_T.lutea           ----------------------------HCEIKHGRVAMAAFVGFIVQA-NGIHFPWAPF 138
Lhcf5_T.lutea           ----------------------------AAELKHSRVAMAAVTGWAWVSSGGPLFPGYLS 96
Lhcf6_T.lutea           ----------------------------HAELKHGRVAMAAIVGFQFHI-NHVHFPGYLS 113
Lhcf7_T.lutea           WRGYPRSEATWESRIELLRRCADLVESYDTTFDRRPVAMAAFVGWLVAV-SGVHFPGLCS 439
Lhcf16_T.lutea          ----------------------------HAEIKHGRVAMAGFVGYCVQA-NGIYWPWPLA 103
Lhcf17_T.lutea          ----------------------------HSEIKHGRVAMAAFVGFCVQS-NGIHFPFDFQ 101
Lhcf21_T.lutea          ----------------------------EMEIKHGRIAMLAVFHVLWTG-SGHKWDGYCS 112
Lhcf23_T.lutea          ----------------------------TAELKHGRVAQLAVLGYV-VP-EFYRFPGEIA 117
Lhcf24_T.lutea          ----------------------------HSELKHGRVAMLATVGYMIGA-AGITFPGE-- 97
Lhcf28_T.lutea          ----------------------------AAELKHCRVAMLATVGWAWTA-TGTHFEGMLS 96
Lhcf1_T.lutea           ----------------------------ELEIKHGRIAMLATLGVITTE-AGFRWPGYLS 94
Lhcf2_T.lutea           ----------------------------EMEIKHGRMAMAAFLGVITTY-SGMRWPGYLS 144
Lhcf3_T.lutea           ----------------------------EIEIKHGRLAMAATLGVITTE-AGIRLPGYLS 132
Lhcf8_T.lutea           ----------------------------AVELKHGRIAMAACTGYF-VQ-SVLRWPGYLS 93
Lhcf9_T.lutea           -----------------------------MEIKHGRAAMLGFLHVIAIH-AGIRFPGYLS 93
Lhcf10_T.lutea          ----------------------------IMEIKHGRAAMLGFLHVIAIH-AGIRFPGYLS 372
Lhcf11_T.lutea          ---GP-LCCMR------------------------------------LTQ-SGLRLPPAS 89
Lhcf12_T.lutea          ----------------------------TAELKHGRVAQLCVVGYV-VP-EFYRFPGEIA 97
Lhcf13_T.lutea          ------------------------------------------------------------ 0
Lhcf14_T.lutea          ----------------------------CMEIKHGRAAMLGFLHVVLIH-AGVRFPGYLS 139
Lhcf15_T.lutea          ----------------------------CMEIKHGRAAMLGFLHVVLIH-AGVRFPGYLS 94
Lhcf18_T.lutea          ----------------------------IMEIKHGRAAMLGFIHVIATH-AGIRLPGYLS 93
Lhcf19_T.lutea          ----------------------------EAELKHGRVAMLACAGMITAD-KFHPL----- 90
Lhcf20_T.lutea          ----------------------------EMEIKHGRIAMAAVLHVVLTE-AGVRWPGYLS 77
Lhcf22_T.lutea          ----------------------------HSEIKHGRVAMAAFVGFVLQS-NGVHFPWGLS 103
Lhcf25_T.lutea          ------------------------YRRRCVEIKHGRICMVAFLGMTVGP-NELIAPSHQL 98
Lhcf26_T.lutea          ----------------------------IMEIKHGRAAMLGFLHVIAIH-AGIRFPGYLS 94
Lhcf27_T.lutea          WQLGN-GDCLNPSRGHWIHAHASVVPRQIMEIKHGRAAMLGFLHVIAIH-AGIRFPGYLS 108
```

Fx302 a404 Fx303 a405 a405 a406 Fx302

```
Lhcf1_P.tricornutum      YS----GTSFESIP--NGFA-ALSAVPGAGIAQIIAFIGFLEIAVMKD------------ 136
Lhcf2_P.tricornutum      LD----GTKFADIP--NGFA-ALSAIPQAGLIQIIAFIGFLETSVMKD------------ 136
Lhcf3_P.tricornutum      YS----GKTFAEIP--NGFA-AFKEIPAGGLVQLLFFIGVLESSVMRD------------ 136
Lhcf4_P.tricornutum      YS----GKTFAEIP--NGFA-AFKEIPAGGLVQLLFFIGVLESSVMRD------------ 136
Lhcf5_P.tricornutum      YS----GTSFESIP--NGFA-ALTTISGAGIAQIVAFIGFLELAVMKD------------ 136
Lhcf6_P.tricornutum      FA----GTKFSDIP--AGFG-SLANIPAAGLCQILFFIGLLETSFMRD------------ 138
Lhcf7_P.tricornutum      FA----GTKFSDIP--AGFG-SLANIPAAGLCQILFFIGLLETSFMRD------------ 138
Lhcf8_P.tricornutum      YS----GLKFADVP--GGFK-ALDTINDAGVLQIVAFIGFLELAFMKE------------ 137
Lhcf9_P.tricornutum      LS----GTKFSDIP--NGYA-AIEAIPYAGKLQLLAFIGALEVFVMRD------------ 136
Lhcf10_P.tricornutum     YS----GTKFTDIP--GGFD-ALSAISKEGLGQIIGFIFFLEM-IMRP------------ 135
Lhcf11_P.tricornutum     YS----GTSFASIP--NGFA-ALSTISTAGIAQIVAFIGFLEIAVMKD------------ 136
Lhcf12_P.tricornutum     -G----GVSYSNIP--AGLA-AFERLPISGLAAIFVAIGFLEVVVMKD------------ 120
Lhcf13_P.tricornutum     P-----GLPCAEVP--NGVA-AIQAIPALGWAQIFFLVGSVDYYGYL------------- 133
Lhcf14_P.tricornutum     P-----GLKFADVP--NGVA-ALSAIPALGWAQIFFLVGAVDYWGVL------------- 132
Lhcf15_P.tricornutum     P-----GLAFKDIP--NGIA-AVNAIPSIFWMTTFFAIGMVDYLNSDN------------ 137
Lhcf16_P.tricornutum     -SK---DISFESL-SGMNPVEQWAGVPDAGKWQIILTIFIAEIATE-------AKK--PH 141
Lhcf17_P.tricornutum     PS---ANLEFAEVP--NGLA-ALDVVPLAGWVQMAVVIGAHEFLV--------------- 140

Lhcf4_T.lutea            NSI-----------TTLSPPEQWDELPDVSLCC-----------TA-------------- 162
Lhcf5_T.lutea            IDQ---GITFESL-G-RDGYAAWAAVPEAGKFQILGTIGILELLQE------GSVK--PH 143
Lhcf6_T.lutea            PSA---GVTFEQL-AGVGPFEAWNLIPLLGKMQILFTIAGLEHASE-----CLDPA--GH 162
Lhcf7_T.lutea            FSE---GVSFEDIS-KLTPLEQWAAVPALGKAQILLAIGIIEHNSEW--------KIKPH 487
Lhcf16_T.lutea           NPGQEDEITHAMISAAGSPPEQWDALPTSAKVQIILFLAIIEYCGE-------QGK--PH 154
Lhcf17_T.lutea           GGG----VSQAQYAAGLSPPEQWDALPFAAKAQIILFIGFLEWWSE-------FGG--QH 148
Lhcf21_T.lutea           YLS-FPPLKFEDIP--AGTWGSWAALPQAGWAQIVAVVAILDNSLF-------------- 155
Lhcf23_T.lutea           P-----GIKFADIP--NGVA-AIEAIPSLGWLQMIFLIGAVDYWGVL------------- 156
Lhcf24_T.lutea           IAK---GVTFASV-GASGPYAAWDATPTAGKLQILTIILALEWAAE-------TKK--PH 144
Lhcf28_T.lutea           TSQ---GISFADA-CAAGPLLGAAKVPAVGVWQIIAAIGALEVFWE-------NKY--PA 143
Lhcf1_T.lutea            KS---LDLKFADVP--GGCFDSYNAVPALGWLQIVGFVIFLELAFG-------------- 135
Lhcf2_T.lutea            TS---EGIKFTDIP--GGAISSWAALPTSAWLQIVIFISALEVYCL-------------- 185
Lhcf3_T.lutea            LS---QDLPYASQP--GTLDGAYFATPLAGWLQIVCFIAALDLAVFR------------- 174
Lhcf8_T.lutea            TS---AGVKFSDLP--NGIA-GFAKIPPLGLAQIFLFIGLMEAFTWPFY--QGG------ 139
Lhcf9_T.lutea            PS---LGVKFSDVP--AGCFASLEAVPTFGWLQIMFFTCMQETGASPFAE---------- 138
Lhcf10_T.lutea           PS---LGVKFSDVP--AGCFASLEAVPTFGWLQIMFFTCMQETGASPFAE---------- 417
Lhcf11_T.lutea           PP---ADVLMPHLS--FVAGSEADA----------SHHC--------------------- 113
Lhcf12_T.lutea           P-----GLKFADIP--NGVA-AIDAIPSLGWLQMIFLIGAVDYWGVL------------- 136
Lhcf13_T.lutea           ----------------------------------------METGYFLFEY---------- 10
Lhcf14_T.lutea           PS---AGLKFADVP--VGCFASLEALPTAGWLQIMLTTCMMETGYFLFEY---------- 184
Lhcf15_T.lutea           PS---AGLKFADVP--VGCFASLEALPTAGWLQIMLTTCMMETGYFLFEY---------- 139
Lhcf18_T.lutea           IA---NDLKFADMP--IGCFASLEAVPTAGWLQIMLTTCMVETGAGNLPEYPGPGTVLAG 148
Lhcf19_T.lutea           -----YDGKLSS-----NPLLAIAQVPKLGLLQILLFIGFMEVFGILN------------ 128
Lhcf20_T.lutea           FA---EDIKFSDMP--GGTWASWAALPNLAWAQIVLIVALLDNSVL-------------- 118
Lhcf22_T.lutea           LDG----TSFADIAAAGGPAAQWDALPTNSKLQILLVIGFLEFWGENSWALSQSGE--KH 157
Lhcf25_T.lutea           LSP-SLDLHFDDIP--GGIA-AIDAVPAAGWFQIIALIGLHELTIA-------------- 140
Lhcf26_T.lutea           PS---LGVKFSDVP--AGCFASLEAVPTFGWLQIMFFTCMQETGASPFAE---------- 139
Lhcf27_T.lutea           PS---LGVKFSDVP--AGCFASLEAVPTFGWLQIMFFTCMQETGASPFAE---------- 153
```

```
                                                 a407
Lhcf1_P.tricornutum        ------------ITG-GEFVGDFRN--NY-LDFGWDTFSE-------------------- 160
Lhcf2_P.tricornutum        ------------ITG-GEFVGDFRN--GY-IDFGWDSFDQ-------------------- 160
Lhcf3_P.tricornutum        ------------LTGEAEFVGDFRN--GA-IDFGWDTFDE-------------------- 161
Lhcf4_P.tricornutum        ------------LTGEAEFVGDFRN--GA-IDFGWDTFDE-------------------- 161
Lhcf5_P.tricornutum        ------------ITG-GEFVGDFRN--DF-IDFGWDSFDE-------------------- 160
Lhcf6_P.tricornutum        ------------WVG-GESVGDFRN--KY-IDFGWDSFSD-------------------- 162
Lhcf7_P.tricornutum        ------------WVG-GESVGDFRN--KY-IDFGWDSFSD-------------------- 162
Lhcf8_P.tricornutum        ------------VEGKSEFVGDFRN--GF-IDFGWDSFDD-------------------- 162
Lhcf9_P.tricornutum        ------------FVG-GEFPGDLRN--NY-IDFGWDSFDD-------------------- 160
Lhcf10_P.tricornutum       ------------IGGRGEFVGDFRN--DA-IDFGWDTFDE-------------------- 160
Lhcf11_P.tricornutum       ------------ITG-GEFPGDFRN--DY-IDFGWDSFDE-------------------- 160
Lhcf12_P.tricornutum       ------------SKGVAQYPGDLRN--GL-FQ--WSA-TP-------------------- 142
Lhcf13_P.tricornutum       --------------------GDF-D-------AGKPD-LDP------------------- 145
Lhcf14_P.tricornutum       --------------------GDF-E-------YGKPD-LDA------------------- 144
Lhcf15_P.tricornutum       -------------------LGYHEI-------APGPE-MDE------------------- 151
Lhcf16_P.tricornutum       YMMGGDLPTMVF------------------PPIDF-SKVDA------------------- 163
Lhcf17_P.tricornutum       ------------KERPGRKPGDFGT---G---YFGVALDDQS------------------ 164

Lhcf4_T.lutea              ----------TNYATPDKLPHPI--PLDLYDPFKFSKNASE------------------- 191
Lhcf5_T.lutea              YMAGGTPGKVP----------------LLWDPLGFTTKLSA------------------- 168
Lhcf6_T.lutea              YTKGGTPGNLKFL-------------KKFWDTPGFTDKLTE------------------- 190
Lhcf7_T.lutea              YMAAAANLVI-------------------------------------------------- 497
Lhcf16_T.lutea             YMRGGKPGAFPKLTENKGIPHPV--PLNLYDPFGWSANMDE------------------- 193
Lhcf17_T.lutea             YMRGGQPGKYPEFK-N--IPLHK--MPNLFDPLGLSKGLSA------------------- 184
Lhcf21_T.lutea             ------------AQDPNREPGDVVG--DR---IPWVRYEDP------------------- 179
Lhcf23_T.lutea             --------------------GDF-D-------IGKPD-LDP------------------- 168
Lhcf24_T.lutea             YMRGGVPGKIDQLP-FEGIPGLWAPKIKFWDPLNFMGALTE------------------- 184
Lhcf28_T.lutea             SECAG---NFG----------------VPWV------TSDP------------------- 159
Lhcf1_T.lutea              ------------ATDPSKEPGDIGG-------PSWVRYDDP------------------- 157
Lhcf2_T.lutea              ------------KQDPAKDPGDVIP--EG---WFWARYPDGYDVWLGDGSTKQIGEEELF 228
Lhcf3_T.lutea              ----------QDPNLPAGDVVQDLP-------INWVRYEDP------------------- 198
Lhcf8_T.lutea              ----------LGKVPEGKLPGDVAG-------DLWVRYSDP------------------- 163
Lhcf9_T.lutea              ------------PQTDDKEPGDIAG-------IPWVRYDDP------------------- 160
Lhcf10_T.lutea             ------------PQTDDKEPGDIAG-------IPWVRYDDP------------------- 439
Lhcf11_T.lutea             ------------------------------------------------------------ 113
Lhcf12_T.lutea             --------------------GNF-D-------AGKPE-LEP------------------- 148
Lhcf13_T.lutea             ----------QGYGALDKEPGDIGG-------EGWVRYDDP------------------- 34
Lhcf14_T.lutea             ----------QGYGALDKEPGDIGG-------EGWVRYDDP------------------- 208
Lhcf15_T.lutea             ----------QGYGALDKEPGDIGG-------EGWVRYDDP------------------- 163
Lhcf18_T.lutea             PMGLKKAGFFGEPQLDSKAPGDIGG-------PLWTRYDDP------------------- 182
Lhcf19_T.lutea             ------------SRRPDYKPGDFLG--SS----QW---DTT------------------- 148
Lhcf20_T.lutea             ------------AQDPAKAPGDVGP-------AFWVRYPDTP----------------GF 143
Lhcf22_T.lutea             YMRGGKPGFYPSLK-KGGIPHPV--PFDLFDPFGFSKNASP------------------- 195
Lhcf25_T.lutea             ------------KQDYTKEPGEIPT-------FLGFKPEDP------------------- 162
Lhcf26_T.lutea             ------------PQTDDKEPGDIAG-------IPWVRYDDP------------------- 161
Lhcf27_T.lutea             ------------PQTDDKEPGDIAG-------IPWVRYDDP------------------- 175
```

Fx304 + c408 +Fx307 via c; a407; a401; c408 + a401; a409; Fx305

```
Lhcf1_P.tricornutum     DKKLQKRAIELNQGRAAQMGILALMVHEQLGVS--ILP---------------------- 196
Lhcf2_P.tricornutum     ETKLRKRAIELNQGRAAQMGILALMVHEQLGVN--ILPGV-------------------- 198
Lhcf3_P.tricornutum     ETQFKKRAIELNQGRAAQMGILALMVHEQLGVS--LLPQ--------------------- 198
Lhcf4_P.tricornutum     ETQFKKRAIELNQGRAAQMGILALMVHEQLGVS--LLPQ--------------------- 198
Lhcf5_P.tricornutum     ETKMQKRAIELNQGRAAQMGILALMVHEQLGVS--LIPN--------------------- 197
Lhcf6_P.tricornutum     EEKARQYNVELNQGRAAQMGILALMVHEQLGNVDDILPFKLI------------------ 204
Lhcf7_P.tricornutum     EEKARQYNVELNQGRAAQMGILALMVHEQLGNVDDILPFKLI------------------ 204
Lhcf8_P.tricornutum     ATKMKKRAIELNQGRAAQMGILALMVHEKLGVS--ILPDL-------------------- 200
Lhcf9_P.tricornutum     ATKARKRTIELNQGRAAQMGILALMVHEQLGVS--IIPSTAASEY---------AF---- 205
Lhcf10_P.tricornutum    ETKLKKRAIELNQGRAAQMGILALMVHEQLGVP--IIPSLP------------------- 199
Lhcf11_P.tricornutum    ETQFKKRAIELNQGRAAQMGILALMVHEKLGVS--LIPN--------------------- 197
Lhcf12_P.tricornutum    EEQLEKRAIELNNGRAAQMGILALMVHEKLNNEPYMINSFLGYSS---------HFNENF 193
Lhcf13_P.tricornutum    AEMEKRQLNELQHGRLAMLAILELLRHDSQNLV---QPGFDGLNNLITG----LPFLYK- 197
Lhcf14_P.tricornutum    DTLAKRQTQELQNGRLAMLATLELLRHDSQNYV---SPGFDGLDNLITG----LPFLY-- 195
Lhcf15_P.tricornutum    ETMNTRRTNEVSNGRLAMLAFFELLRHDWQNTV---QPGFDGFDRLITG----LPFLYN- 203
Lhcf16_P.tricornutum    ATLKTKRSRELNNGRLAMIGIMSFISEYNIPGSVPVLSG---LDAF-------------- 206
Lhcf17_P.tricornutum    AKQLRLLNVEVSNGRLAMLGILGMFASEIIHGEALFETKIFS------------------ 206

Lhcf4_T.lutea           EKKAAGLLKEINNGRLAMLGIMGFLAEAKVPGSVPLLTG---LIKPYGG-EVMAPFYTTI 247
Lhcf5_T.lutea           DTLATKRTSELKNGRLAMIGVMSLVSAHFIPGSVPLLP---------------------- 206
Lhcf6_T.lutea           EQKATKRVSELKNGRLAMIGMASIISAMSIPGSVPLLNG---APALTGT-GFVLPFGDF- 245
Lhcf7_T.lutea           ------------------------------------------------------------ 497
Lhcf16_T.lutea          ATKERRLLMEINNGRLAMLGIFSFMSASKG--LIVPGLD---FIPPYAG-EYMGYFSASD 247
Lhcf17_T.lutea          EQRETKLCAEINNGRLAMLGVFGFYCAEKIPGSVPLL-S---FIKPYAG-EIMGPF---- 235
Lhcf21_T.lutea          EVKFFKLNAERNNGRAAMMGIIGMMTHESLTGNPIYPLPYEA------------------ 221
Lhcf23_T.lutea          EEMDRRKTCELQHGRLAMLAILELLRHDAQNFV---VPGFDGMDHLITG----LPFLYS- 220
Lhcf24_T.lutea          EQKARKRKSELKNGRLAMIGIISFLTGHNIPGSVPALDS---HF---------------- 225
Lhcf28_T.lutea          AKMKEIQLAELKNGRLAMIGIISFACAESIPGSVPFYPF--------------------- 198
Lhcf1_T.lutea           ETKKRKLNIERNNGRAAMMGILGMMTHNALGVDALFPIVGGN------------------ 199
Lhcf2_T.lutea           LGKTWKLNAERNNGRAAMMGITGMVIHEALTGNPVFPIGESL------------------ 270
Lhcf3_T.lutea           EVKTFKLNVERNNGRAAMLGITGMISHVALGQDALFPIVSN------------------- 239
Lhcf8_T.lutea           EVKKHKLNVEINNGRAAMMGSLGMLMHDHILGTWIPPGF--------------------- 202
Lhcf9_T.lutea           ETKAFKLNVERQNGRAAMMGILGCFVHELLGVDALYPTGGLGGAAP----PEIF------ 210
Lhcf10_T.lutea          ETKAFKLNVERQNGRAAMMGILGCFVHELLGVDALYPTGGLGGAAP----PEIF------ 489
Lhcf11_T.lutea          ------------------------------------------------------------ 113
Lhcf12_T.lutea          EELERRQLCELQHGRLAMLASLELLRHDSQNLV---VPGFDGLDNLITG----FPFLYN- 200
Lhcf13_T.lutea          ETKTFKLNVERQNGRAAMMGILGCFVHELLGVDALYPTGGLGGDAP----PTIF------ 84
Lhcf14_T.lutea          ETKTFKLNVERQNGRAAMMGILGCFVHELLGVDALYPTGGLGGDAP----PTIF------ 258
Lhcf15_T.lutea          ETKTFKLNVERQNGRAAMMGILGCFVHELLGVDALYPTGGLGGDAP----PTIF------ 213
Lhcf18_T.lutea          EVKAYKLNIERNNGRAAMMGITGCLVHELLGVDALYPTGGLGGAAP----PPIFS----- 233
Lhcf19_T.lutea          EVWDNYQLRELNNGRLAMFASIGMLTHAYITGKGPLELLDGSSIV----------F---- 194
Lhcf20_T.lutea          SGKEFKLNVERNNGRAAMMGIIGMMIHESLTGNPVWPIPVPPQPVL----EAALDDVEKG 199
Lhcf22_T.lutea          EKKADGLIKEINNGRAAMLGIMGFVAASKVPGSVPALNG---IITPYSG-EVMAPFSGSD 251
Lhcf25_T.lutea          EVFRNKQLKELKNGRLAMIAVLGELMAQQVSGMGTYEQLGTIVDSVTEATGIALPF---- 218
Lhcf26_T.lutea          EVKAFKLNVERQNGRAAMMGILGCFVHELLGVDALYPTGGLGGAAP----PEIF------ 211
Lhcf27_T.lutea          EVKAFKLNVERQNGRAAMMGILGCFVHELLGDQDSDEENDITDSNA----SFELSFHQ-- 229
```

Fig. S4 Evolution of the intracellular N:C ratio (purple curve), the Fx:C ratio (orange), the Chl *a*:C ratio (grey) and the sine light (green). Grey dashed line marks the transition from repletion to depletion in N.

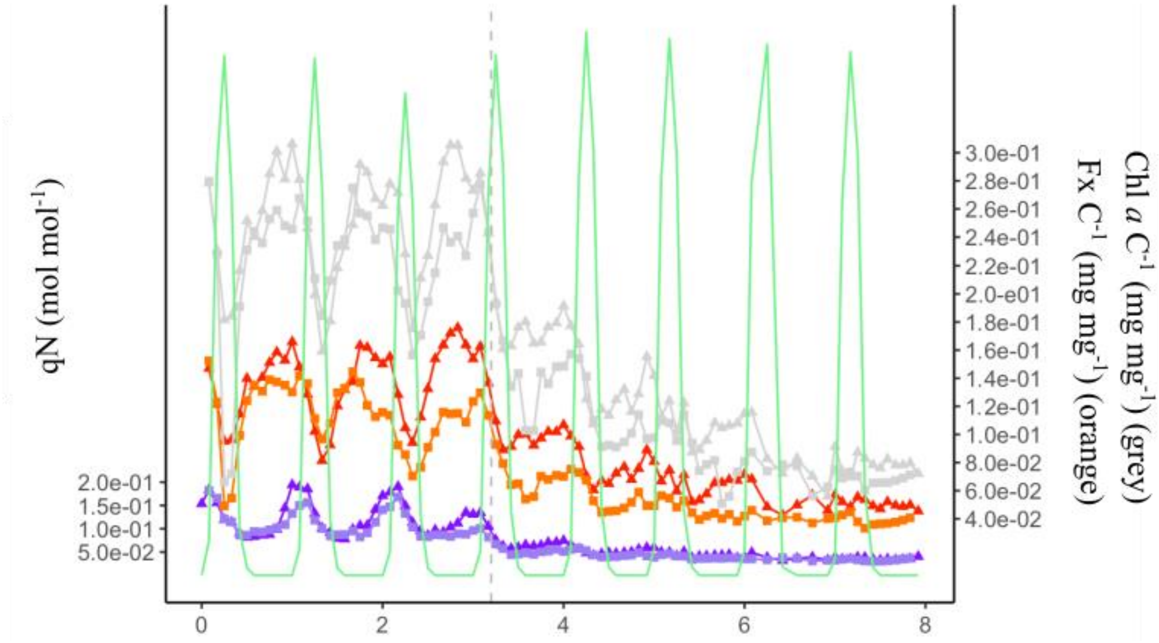

Constant light experiment:

Table S8 *lhcf* normalized counts in constant light experiment for limitation, repletion and depletion phases in NO_3

	Expression rate of *lhcf* genes for replicate 1 and 2 in NO_3 limitation (lim), repletion (rep) and depletion (dep)					
	**lim_replicate1**	**rep_replicate1**	**dep_replicate1**	**lim_replicate2**	**rep_replicate2**	**dep_replicate2**
lhcf1	2970.52	9904.49	1916.64	3409.13	7338.34	2747.63
lhcf2	4280.57	5999.78	1934.12	3171.96	5473.43	2915.70
lhcf3	4858.00	14205.26	3405.18	6010.68	12109.27	4832.48
lhcf4	3143.80	3384.57	1468.88	2146.67	3845.85	1876.25
lhcf5	5610.91	7185.87	3715.34	4957.41	9562.95	4651.30
lhcf6	6166.96	22892.63	5218.08	12004.33	19639.59	9036.77
lhcf7	6306.79	8695.64	3404.09	4176.58	5750.85	3722.71
lhcf8	2362.92	4433.13	1405.54	2067.61	4917.54	2006.18
lhcf9	9273.47	16488.52	5022.59	8410.33	13941.48	7861.43
lhcf10	2369.50	4087.75	1443.76	1088.54	3794.17	1064.48
lhcf11	59.77	93.07	24.03	17.03	53.80	11.92
lhcf12	3035.77	2409.42	1349.84	2605.19	2919.73	2686.83
lhcf13	12486.92	57891.31	14564.31	26518.98	42633.49	20322.90
lhcf14	519.31	1810.69	334.18	767.45	1456.70	544.76
lhcf15	837.91	1649.37	625.78	926.78	1811.12	598.40
lhcf16	3568.24	6601.61	4298.52	2836.28	7803.52	2393.59
lhcf17	5246.25	7517.82	3037.14	4236.18	8772.89	3664.30
lhcf18	4289.89	6086.64	2461.60	3321.56	6183.32	3064.71
lhcf19	4068.35	15080.10	2672.38	4743.35	11568.15	3731.05
lhcf20	6651.72	15095.61	4377.16	7627.07	17307.40	6733.77
lhcf21	2122.74	1211.95	1227.53	1651.66	4463.97	1925.13
lhcf22	2.19	5.17	2.18	6.08	6.33	2.38
lhcf23	279.67	166.49	151.80	116.76	138.18	126.36

lhcf24	10774.36	13180.48	5150.37	7395.98	14123.97	6568.08
lhcf25	4756.01	17000.40	2795.79	6060.54	15956.18	4726.39
lhcf26	1350.09	1862.39	2293.42	2638.03	4404.91	2163.53
lhcf27	2518.11	3103.30	1661.09	1638.28	5228.72	1518.64
lhcf28	3793.07	7935.59	2622.14	3742.38	8156.88	31<br>60.07

Table S9 *lhcr* normalized counts in constant light experiment for limitation, repletion and depletion phases in NO_3

	Expression rate of *lhcr* genes for replicate 1 and 2 in NO_3 limitation (lim), repletion (rep) and depletion (dep)					
	**lim_replicate1**	**rep_replicate1**	**dep_replicate1**	**lim_replicate2**	**rep_replicate2**	**dep_replicate2**
lhcr1	4390.24	6929.42	2266.12	4401.59	7332.02	3660.72
lhcr2	2949.13	9381.24	1767.03	3203.59	6543.01	2461.54
lhcr3	1589.17	10661.44	1913.37	4373.61	6481.83	3058.75
lhcr4	1904.49	9353.32	1639.25	3035.74	7325.69	2379.29
lhcr5	33.45	44.47	46.96	90.00	136.07	114.43
lhcr6	631.17	906.89	847.47	694.48	873.39	882.10
lhcr7	4453.86	9815.56	3186.76	5317.42	9539.74	4659.64
lhcr8	6410.44	8177.56	3262.12	4884.44	7800.35	4135.15
lhcr9	0.00	0.00	0.00	0.00	0.00	0.00
lhcr10	625.14	798.32	406.26	375.82	481.00	333.77
lhcr11	1806.88	3376.30	885.70	1071.51	2748.85	1027.53
lhcr12	1913.26	3196.36	1208.96	2059.10	3458.74	1939.43

Table S10 *lhcx* normalized counts in constant light experiment for limitation, repletion and depletion phases in NO_3

	**Expression rate of *lhcx* genes for replicate 1 and 2 in NO_3 limitation (lim), repletion (rep) and depletion (dep)**					
	**lim_replicate1**	**rep_replicate1**	**dep_replicate1**	**lim_replicate2**	**rep_replicate2**	**dep_replicate2**
lhcx1	997.48	1986.48	819.08	949.89	2003.09	920.25
lhcx2	2962.84	5679.21	5209.34	6218.65	10539.71	5913.65
lhcx3	1175.70	1088.89	1294.14	1356.11	1983.05	1710.56
lhcx4	662.43	632.86	575.54	470.69	593.86	498.27
lhcx5	2162.77	9130.99	2101.21	3563.59	6662.21	2791.73
lhcx6	3.29	1.03	0.00	2.43	3.16	1.19
lhcx7	9.32	6.20	22.93	15.81	20.04	7.15
lhcx8	171.64	376.41	148.53	193.38	378.68	164.50
lhcx9	76.77	191.31	83.00	102.16	321.72	134.70
lhcx10	229.77	216.12	346.20	313.79	495.76	283.70
lhcx11	83.90	84.80	70.99	37.70	84.39	52.45
lhcx12	100.35	82.73	90.64	62.03	139.24	104.90

Dynamic light experiment:

Table S11 *lhcf* normalized counts in dynamic light experiment for repletion and depletion phases in NO_3, replicate 1

	**Expression rate of *lhcf* genes for replicate 1 in NO_3 repletion (rep) and NO_3 depletion (dep) at 8:00 h, 12:00 h and 00:00 h**					
	**8_rep replicate1**	**12_rep replicate1**	**00_rep replicate1**	**8_dep replicate1**	**12_dep replicate1**	**00_dep replicate1**
Lhcf1	76262.95	83761.74	4968.35	3289.91	1890.47	2331.65
Lhcf2	64764.74	81762.74	2821.91	6105.84	5318.60	2197.84

Lhcf3	99425.51	121490.40	5774.44	1618.83	1106.00	1527.95
Lhcf4	13151.03	55143.82	1515.69	390.73	343.29	367.14
Lhcf5	146373.06	255069.20	14081.30	6880.01	8186.59	8636.64
Lhcf6	198834.20	257088.50	4211.68	4282.84	3964.47	1958.65
Lhcf7	35323.16	55681.48	5890.94	4990.16	4610.94	4307.87
Lhcf8	65572.22	90600.88	4359.96	2413.05	2160.34	3688.16
Lhcf9	199660.61	222541.20	11073.46	1332.01	1996.51	2739.77
Lhcf10	25658.60	48066.42	7643.16	624.68	2561.41	3470.71
Lhcf11	11397.28	26058.33	1046.15	45.58	83.61	115.41
Lhcf12	68085.09	152386.40	1656.90	1937.24	1880.27	1431.77
Lhcf13	480778.13	557008.40	56492.31	7004.58	10637.87	17696.46
Lhcf14	39736.98	71588.10	1583.94	711.58	489.44	568.70
Lhcf15	18893.83	36059.69	1979.34	122.14	247.44	674.91
Lhcf16	1038.79	3109.98	5687.36	1338.08	3986.23	12202.70
Lhcf17	33762.87	93110.78	2841.91	894.49	819.81	1926.04
Lhcf18	82502.02	186803.80	10170.88	7671.19	7396.68	5150.87
Lhcf19	179974.01	238761.20	11613.60	7704.61	6861.02	4225.91
Lhcf20	197501.01	217118.80	13612.95	9102.25	7619.65	2998.20
Lhcf21	22100.63	2523.91	2617.15	1757.37	1380.63	2003.81
Lhcf22	0.00	1.27	17.65	20.05	8.84	96.18
Lhcf23	700.24	848.52	349.50	1031.82	1150.19	667.38
Lhcf24	334190.59	458843.40	10179.11	670.86	558.10	1704.41
Lhcf25	362816.27	335793.50	21119.60	10039.88	5175.16	3139.53
Lhcf26	1299.54	7660.92	3562.10	52.26	366.40	360.45
Lhcf27	28064.23	41330.47	6813.53	1950.61	2163.74	3740.01
Lhcf28	35369.42	75639.61	2753.66	4234.83	4457.31	3350.28

Table S12 *lhcf* normalized counts in dynamic light experiment for repletion and depletion phases in NO_3, replicate 2

	Expression rate of *lhcf* genes for replicate 2 in NO_3 repletion (rep) and NO_3 depletion (dep) at 8:00 h, 12:00 h and 00:00 h					
	**8_rep replicate2**	**12_rep replicate2**	**00_rep replicate2**	**8_dep replicate2**	**12_dep replicate2**	**00_dep replicate2**
Lhcf1	53444.25	46217.67	4310.83	37520.73	32004.03	4698.05
Lhcf2	43596.03	38818.64	2456.68	32205.82	30254.42	2436.08
Lhcf3	79890.67	70149.45	5429.15	51124.89	43213.98	5104.98
Lhcf4	29370.36	43054.69	1403.03	14197.37	16279.44	1416.04
Lhcf5	222256.20	113490.50	13227.45	138149.10	71405.58	12768.25
Lhcf6	156744.40	162384.70	3649.14	87756.85	82501.03	3324.35
Lhcf7	39017.87	42370.57	5175.20	22513.68	22048.60	4560.14
Lhcf8	68556.19	35959.58	5458.33	41341.17	25554.56	5154.82
Lhcf9	204839.30	90942.02	11399.33	130956.70	63460.78	11869.05
Lhcf10	32318.59	8964.88	9150.06	28546.68	14035.60	7495.31
Lhcf11	12188.00	5175.32	983.46	14074.57	8120.78	951.08
Lhcf12	74226.60	79794.70	2127.81	52699.48	57081.19	2198.48
Lhcf13	497057.70	259498.80	50703.91	309816.30	184174.50	48578.87
Lhcf14	41897.44	15798.60	2124.66	46694.59	24707.54	1954.05
Lhcf15	24497.48	9216.27	2045.00	24852.16	7071.69	2915.37
Lhcf16	5565.83	1669.66	8796.74	3609.18	2780.18	8324.18
Lhcf17	59014.31	58934.59	2886.51	27291.58	25257.34	3084.02
Lhcf18	77988.21	78906.72	9278.62	59140.44	68115.17	8595.23
Lhcf19	150564.80	122316.60	10165.07	100987.40	94668.30	9540.17
Lhcf20	148432.10	131954.40	8620.08	87916.08	72741.78	8855.36
Lhcf21	62314.30	2605.17	2068.66	32687.67	1831.28	2041.44

Lhcf22	12.68	7.50	16.56	7.28	10.95	8.88
Lhcf23	544.01	536.54	589.13	765.96	929.53	555.08
Lhcf24	379815.00	188786.60	14486.15	221456.90	114875.50	12385.22
Lhcf25	261520.20	237195.40	12333.11	160553.50	126493.00	12695.19
Lhcf26	4807.38	8938.62	1511.87	2650.69	4859.00	1591.51
Lhcf27	75560.75	20172.23	5217.00	42498.44	11575.37	5915.41
Lhcf28	44438.99	41571.39	2925.15	27978.45	26188.56	2791.79

Table S13 *lhcr* normalized counts in dynamic light experiment for repletion and depletion phases in NO_3, replicate 1

	**Expression rate of *lhcr* genes for replicate 1 in NO_3 repletion (rep) and NO_3 depletion (dep) at 8:00 h, 12:00 h and 00:00 h**					
	**8_rep replicate1**	**12_rep replicate1**	**00_rep replicate1**	**8_dep replicate1**	**12_dep replicate1**	**00_dep replicate1**
Lhcr1	91035.26	105841.20	8943.50	3456.41	2970.64	4664.14
Lhcr2	64768.94	106403.00	6059.22	5477.51	5131.66	5305.59
Lhcr3	68373.18	166782.00	8022.08	1686.28	1364.32	2198.68
Lhcr4	77230.25	97733.07	3868.06	1396.42	1136.59	786.97
Lhcr5	35047.69	12851.43	1054.39	3864.16	1541.74	71.92
Lhcr6	94191.59	19954.31	162.40	15926.96	5241.78	71.09
Lhcr7	117211.16	132541.60	8857.59	6626.61	5217.99	5339.05
Lhcr8	128408.67	178881.70	14684.99	8475.13	8538.71	6409.53
Lhcr9	0.00	0.00	0.00	0.00	0.00	0.00
Lhcr10	5357.98	10383.58	1580.41	1517.34	1442.49	1976.22
Lhcr11	50261.59	110997.30	7276.01	546.29	698.81	710.03
Lhcr12	22485.45	37173.22	2846.62	1638.88	1330.33	1374.07

Table S14 *lhcr* normalized counts in dynamic light experiment for repletion and depletion phases in NO_3, replicate 2

	Expression rate of *lhcr* genes for replicate 2 in NO_3 repletion (rep) and NO_3 depletion (dep) at 8:00 h, 12:00 h and 00:00 h					
	**8_rep replicate2**	**12_rep replicate2**	**00_rep replicate2**	**8_dep replicate2**	**12_dep replicate2**	**00_dep replicate2**
Lhcr1	84206.86	79318.19	6101.09	45123.11	37638.47	6630.94
Lhcr2	72215.34	71457.66	6303.78	49057.00	42629.66	6676.68
Lhcr3	95183.24	69135.15	7153.17	63012.92	58681.77	6391.29
Lhcr4	26480.22	21996.98	3085.25	22739.52	31096.39	2519.37
Lhcr5	30258.74	14216.49	264.20	26401.78	5451.74	261.50
Lhcr6	75016.74	20043.41	98.58	97520.79	12234.63	115.39
Lhcr7	111290.30	102096.80	6315.61	54507.20	42495.78	6509.41
Lhcr8	154249.30	150572.00	10583.06	78495.57	65020.94	10928.21
Lhcr9	0.00	0.00	0.00	0.00	0.00	0.00
Lhcr10	3585.20	6817.46	1856.51	2708.97	4691.44	1698.70
Lhcr11	66486.83	59439.87	5805.35	40534.62	37256.22	6088.83
Lhcr12	21081.29	17421.99	2221.66	15221.43	13276.99	2380.09

Table S15 *lhcx* normalized counts in dynamic light experiment for repletion and depletion phases in NO_3, replicate 1

	Expression rate of *lhcx* genes for replicate 1 in NO_3 repletion (rep) and NO_3 depletion (dep) at 8:00 h, 12:00 h and 00:00 h

	8_rep replicate1	12_rep replicate1	00_rep replicate1	8_dep replicate1	12_dep replicate1	00_dep replicate1
Lhcx1	236060.41	126845.30	390.69	11676.93	4936.56	108.72
Lhcx2	1129.21	3487.10	15325.15	2371.73	2360.19	8277.86
Lhcx3	103067.59	4985.39	162.40	48386.11	19117.44	632.26
Lhcx4	81824.91	48602.80	192.99	15023.96	8546.19	272.64
Lhcx5	169205.47	176894.20	10267.37	6672.79	4421.97	3709.06
Lhcx6	0.00	0.00	0.00	0.00	0.68	12.54
Lhcx7	0.00	1.27	7.06	12.15	18.35	79.45
Lhcx8	230691.91	70000.62	175.34	10083.02	3897.18	40.98
Lhcx9	205842.90	29632.06	228.29	2148.71	740.28	112.07
Lhcx10	109777.69	25642.99	115.32	3291.13	1297.02	77.78
Lhcx11	2649.55	1968.42	756.67	3088.16	1775.58	360.45
Lhcx12	162154.72	9012.69	83.55	5126.28	1157.67	20.91

Table S16 *lhcx* normalized counts in dynamic light experiment for repletion and depletion phases in NO_3, replicate 2

	Expression rate of *lhcx* genes for replicate 2 in NO_3 repletion (rep) and NO_3 depletion (dep) at 8:00 h, 12:00 h and 00:00 h					
	**8_rep replicate2**	**12_rep replicate2**	**00_rep replicate2**	**8_dep replicate2**	**12_dep replicate2**	**00_dep replicate2**
Lhcx1	106710.00	114741.20	257.89	73596.95	52759.37	201.41
Lhcx2	2448.59	3927.14	17879.77	2828.65	3164.96	21336.85
Lhcx3	160344.40	11833.94	313.89	186482.80	10451.34	434.92
Lhcx4	39088.65	35029.07	164.04	33745.03	18974.58	155.67
Lhcx5	128518.00	111739.60	8451.31	96790.21	76227.53	8830.10
Lhcx6	0.00	0.00	0.79	0.00	0.84	0.00
Lhcx7	1.06	11.26	13.41	3.12	17.68	17.07
Lhcx8	96415.99	44973.24	110.41	74098.57	27392.57	81.93

Lhcx9	91584.31	14579.19	197.17	76105.06	9105.04	132.45
Lhcx10	68723.09	10640.79	82.02	88282.41	6310.55	104.46
Lhcx11	3475.35	1682.17	473.99	4144.11	1319.36	461.54
Lhcx12	82215.67	7893.05	40.22	61447.69	4059.13	32.09

Constant light experiment:

Table S17 p-value of the overexpression of FCP genes in NO_3 repletion compared to limitation, and underexpression in NO_3 depletion compared to repletion in constant light experiment. Blank rows mean the gene is not significantly overexpressed.

	Over-expression	Under-expression		Over-expression	Under-expression		Over-expression	Under-expression
lhcf	**lim vs rep**	**dep vs rep**	*lhcr*	**lim vs rep**	**dep vs rep**	*lhcx*	**lim vs rep**	**dep vs rep**
lhcf1		1.51E-05	*lhcr1*		7.93E-03	*lhcx1*	1.78E-02	2.28E-03
lhcf2		1.20E-02	*lhcr2*	6.14E-03	1.26E-05	*lhcx2*		
lhcf3	8.89E-03	9.32E-05	*lhcr3*		1.26E-02	*lhcx3*		
lhcf4		2.43E-02	*lhcr4*	6.99E-04	1.81E-05	*lhcx4*		
lhcf5		3.39E-02	*lhcr5*			*lhcx5*	8.41E-03	8.06E-04
lhcf6		8.43E-03	*lhcr6*			*lhcx6*		
lhcf7			*lhcr7*		3.83E-03	*lhcx7*		
lhcf8	3.25E-02	7.50E-04	*lhcr8*		1.39E-02	*lhcx8*	3.99E-02	5.23E-03
lhcf9		1.00E-02	*lhcr9*			*lhcx9*	2.51E-02	
lhcf10		4.27E-03	*lhcr10*			*lhcx10*		
lhcf11		5.05E-02	*lhcr11*		5.08E-04	*lhcx11*		

lhcf12			*lhcr12*		3.16E-02	*lhcx12*		
lhcf13	4.27E-02	1.15E-02						
lhcf14	2.20E-02	1.59E-04						
lhcf15	3.22E-02	7.51E-05						
lhcf16	5.23E-02	5.02E-02						
lhcf17		2.31E-03						
lhcf18		8.79E-03						
lhcf19	4.78E-04	8.29E-07						
lhcf20	2.20E-02	5.67E-04						
lhcf21								
lhcf22								
lhcf23								
lhcf24		9.52E-03						
lhcf25	1.46E-03	2.70E-06						
lhcf26								
lhcf27		1.09E-02						
lhcf28	1.05E-02	7.68E-05						

Fig. S5 MA-plot N limitation vs N repletion in constant light experiment. Upper graph red points: overexpressed genes in limitation. Lower graph red points: overexpressed genes in repletion. Grey points: no overexpression.

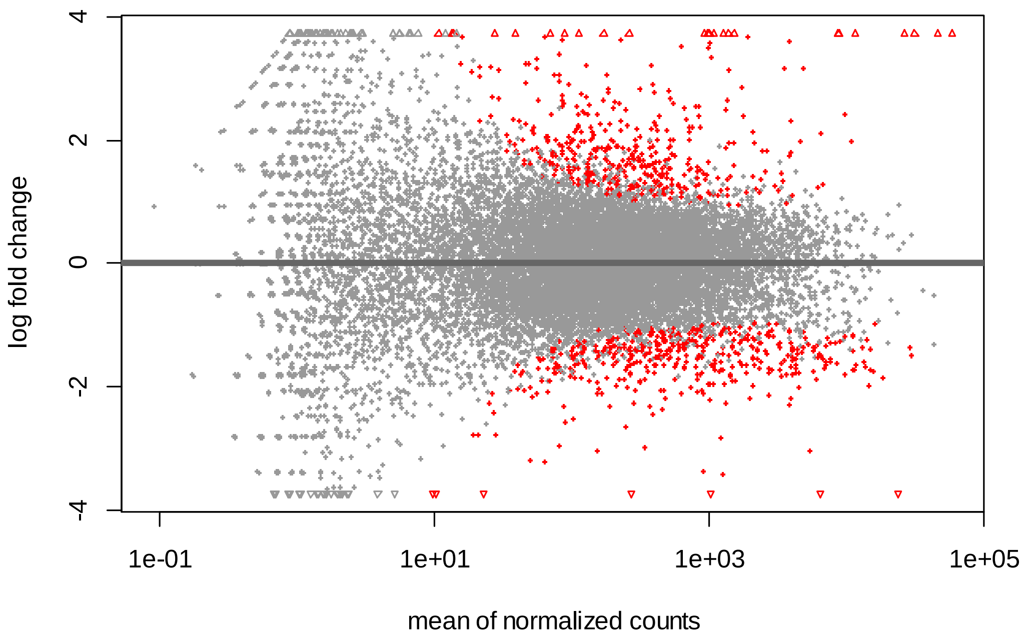

Fig. S6 MA-plot N limitation vs N depletion in constant light experiment. Absence of red points (overexpressed genes) in both conditions relative to one another.

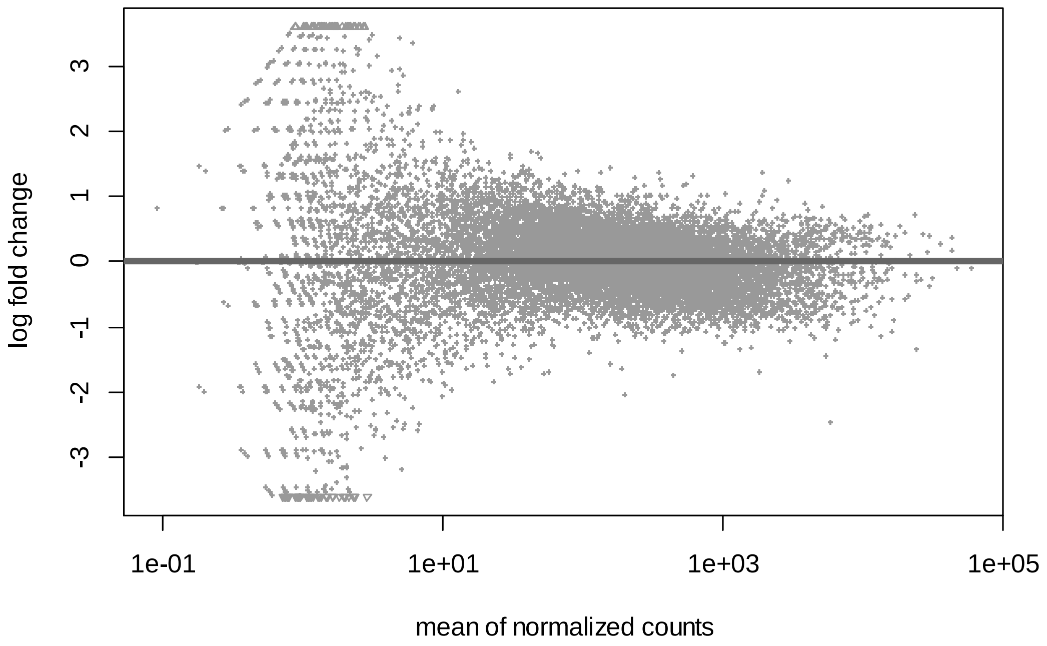

Fig. S7 MA-plot N repletion vs N depletion in constant light experiment. Upper graph red points: overexpressed genes in repletion. Lower graph red points: underexpressed genes in depletion. Grey points: no overexpression.

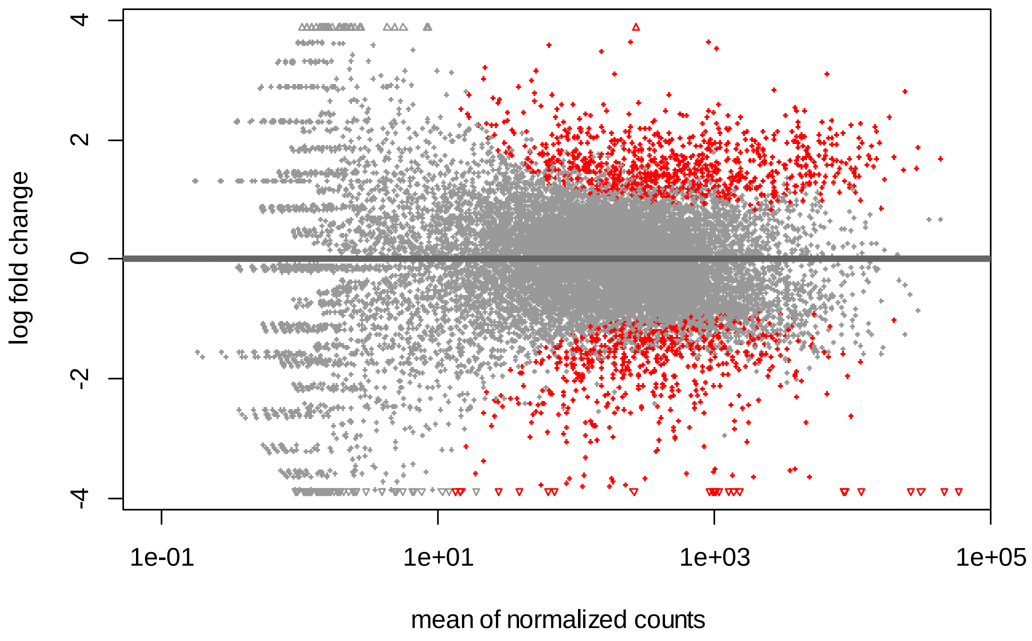

Dynamic light experiment:

Table S18 p-value of the overexpression of FCP genes at 8:00 h and 12:00 h compared to 00:00 h in the dynamic light experiment. Blank rows mean the gene is not significantly overexpressed.

	Overexpression at :			Overexpression at :			Overexpression at :	
lhcf	**8 vs 00**	**12 vs 00**	*lhcr*	**8 vs 00**	**12 vs 00**	*lhcx*	**8 vs 00**	**12 vs 00**
lhcf1	9.65E-04	2.05E-03	*lhcr1*	3.19E-03	5.11E-03	*lhcx1*	1.01E-22	6.93E-20
lhcf2	1.03E-06	8.30E-07	*lhcr2*	9.34E-04	5.07E-04	*lhcx2*	9.30E-13	3.70E-08
lhcf3	2.24E-03	3.47E-03	*lhcr3*	7.85E-03	4.36E-03	*lhcx3*	1.64E-50	1.96E-17
lhcf4	4.69E-03	2.88E-04	*lhcr4*	2.68E-03	2.02E-03	*lhcx4*	4.42E-39	5.49E-33
lhcf5	8.59E-04	3.10E-03	*lhcr5*	8.74E-11	5.09E-06	*lhcx5*	3.20E-04	8.90E-04
lhcf6	1.93E-06	1.18E-06	*lhcr6*	1.46E-57	1.53E-32	*lhcx6*		
lhcf7	3.70E-03	1.52E-03	*lhcr7*	3.71E-04	7.35E-04	*lhcx7*	3.27E-02	
lhcf8	2.00E-03	6.85E-03	*lhcr8*	1.16E-03	1.13E-03	*lhcx8*	2.99E-30	1.12E-21
lhcf9	2.85E-03	1.84E-02	*lhcr9*			*lhcx9*	2.87E-19	3.13E-09
lhcf10			*lhcr10*		1.28E-02	*lhcx10*	7.56E-27	6.20E-14
lhcf11	1.45E-02	2.09E-02	*lhcr11*	3.11E-02	2.13E-02	*lhcx11*	2.33E-20	3.28E-08
lhcf12	9.94E-06	7.96E-07	*lhcr12*	3.88E-03	2.98E-03	*lhcx12*	9.39E-38	1.18E-15
lhcf13	1.58E-02							
lhcf14	3.48E-04	1.16E-03						
lhcf15	2.20E-02							
lhcf16	1.20E-02	1.99E-02						
lhcf17	3.58E-03	9.16E-04						
lhcf18	2.65E-03	2.95E-04						
lhcf19	3.13E-04	3.75E-04						
lhcf20	2.49E-04	5.08E-04						

lhcf21	7.73E-07	
lhcf22		
lhcf23		
lhcf24	2.21E-03	7.57E-03
lhcf25	4.30E-04	1.38E-03
lhcf26		
lhcf27	6.98E-03	
lhcf28	9.88E-05	1.35E-05

Table S19 p-value of the overexpression of FCP genes at 8:00 h compared to 12:00 h in the dynamic light experiment. Missing genes mean they are not significantly overexpressed.

Overexpression at :					
lhcf	**8 vs 12**	*lhcr*	**8 vs 12**	*lhcx*	**8 vs 12**
lhcf21	7.16E-06	*lhcr6*	3.24E-03	*lhcx3*	1.80E-07
				lhcx11	1.59E-02
				lhcx12	3.09E-04

Fig. S8 Overexpression (red points) at 8:00 h vs 12:00 h in dynamic light experiment. Upper graph red points: overexpressed or underexpressed genes at 8:00 h. Lower graph red points: overexpressed or underexpressed genes at 12:00 h. Grey points: no overexpression.

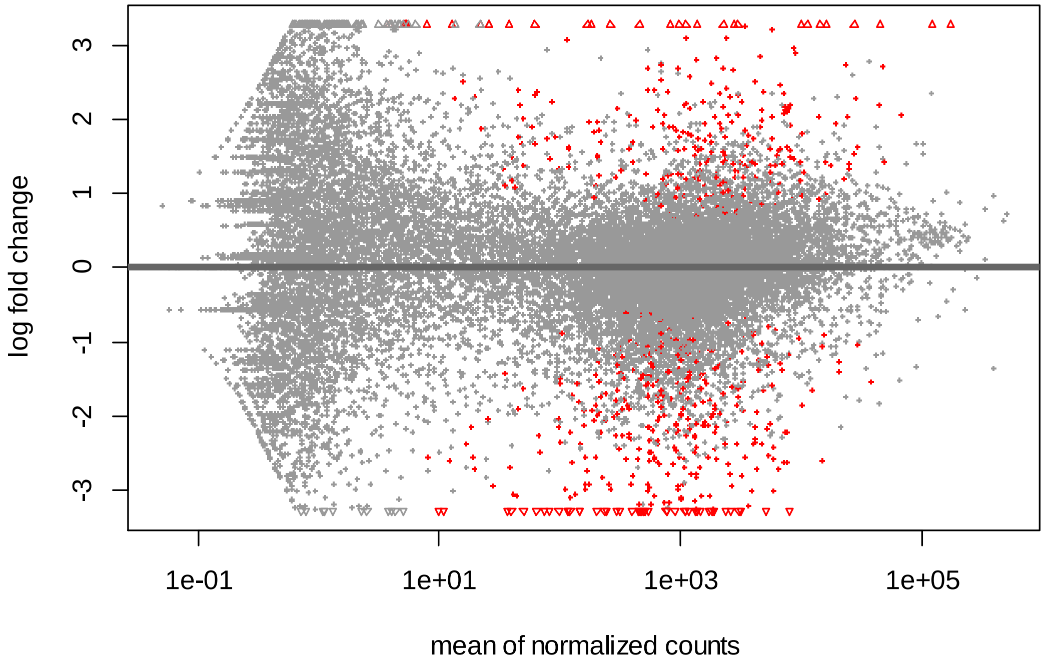

Fig. S9 Overexpression (red points) at 8:00 h vs 00:00 h in dynamic light experiment. Upper graph red points: overexpressed or underexpressed genes at 00:00 h. Lower graph red points: overexpressed or underexpressed genes at 00:00 h. Grey points: no overexpression.

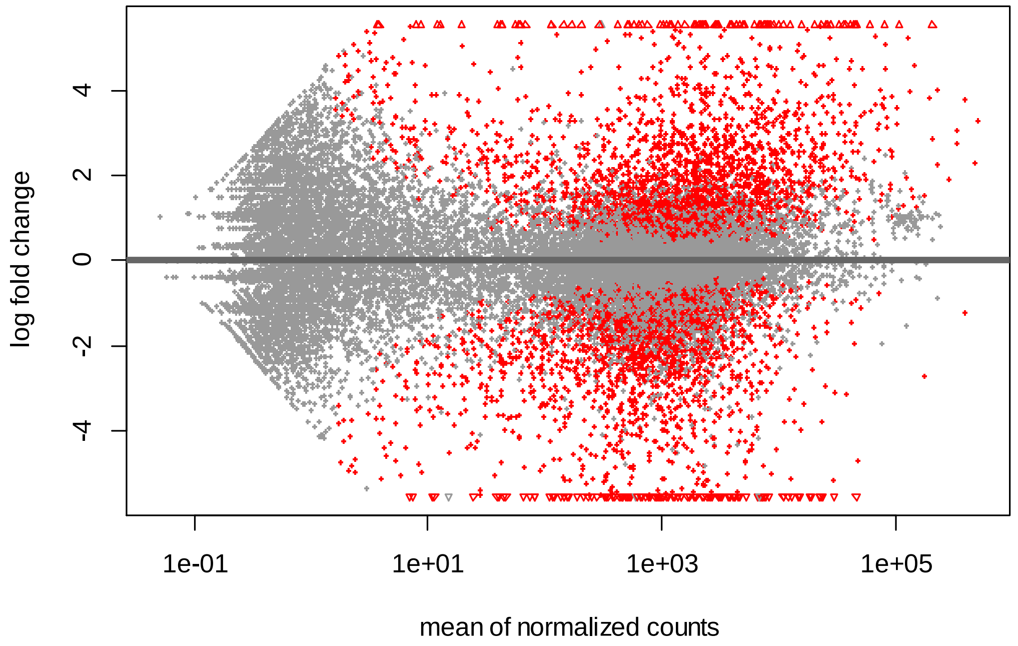

Fig. S10 Overexpression (red points) at 12:00 h vs 00:00 h in dynamic light experiment. Upper graph red points: overexpressed or underexpressed genes at 12:00 h. Lower graph red points: overexpressed or underexpressed genes at 00:00 h. Grey points: no overexpression.

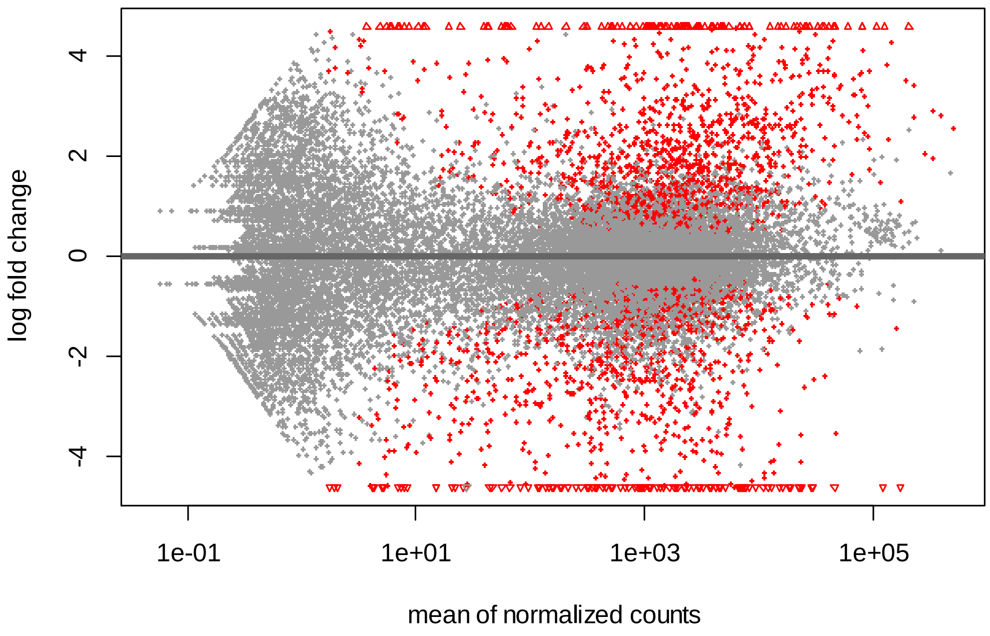

Supplement: Supplementary file 1 [file Data_Sheet_1.PDF]
